# Toward robust *N*-glycomics of various tissue samples that may contain glycans with unknown or unexpected structures

**DOI:** 10.1038/s41598-021-84668-x

**Published:** 2021-03-18

**Authors:** Noriko Suzuki, Tatsuya Abe, Ken Hanzawa, Shunji Natsuka

**Affiliations:** 1 Graduate School of Science and Technology, Niigata University, 8050 Ikarashi-nino-cho, Nishi-ku, Niigata, 950-2181 Japan; 2 Faculty of Science, Niigata University, 8050 Ikarashi-nino-cho, Nishi-ku, Niigata, 950-2181 Japan

**Keywords:** Glycobiology, Glycomics

## Abstract

Glycans in tissues are structurally diverse and usually include a large number of isomers that cannot be easily distinguished by mass spectrometry (MS). To address this issue, we developed a combined method that can efficiently separate and identify glycan isomers. First, we separated 2-aminopyridine (PA)-derivatized *N*-glycans from chicken colon by reversed-phase liquid chromatography (LC) and directly analyzed them by electrospray ionization (ESI)-MS and MS/MS to obtain an overview of the structural features of tissue glycans. Next, we deduced the structures of isomers based on their elution positions, full MS, and MS/MS data, before or after digestions with several exoglycosidases. In this method, the elution position differed greatly depending on the core structure and branching pattern, allowing multiantennary *N*-glycan structures to be easily distinguished. To further determine linkages of branch sequences, we modified PA-*N*-glycans with sialic acid linkage-specific alkylamidation and/or permethylation, and analyzed the products by LC–MS and multistage MS. We determined the relative abundances of core structures, branching patterns, and branch sequences of *N*-glycans from chicken colon, and confirmed presence of characteristic branch sequences such as Le^x^, sialyl Le^x^, sulfated LacNAc, LacNAc repeat, and LacdiNAc. The results demonstrated that our method is useful for comparing *N*-glycomes among various tissue samples.

## Introduction

*N*-Glycosylation on glycoproteins, one of the most prominent glycan modifications in eukaryotes, involves highly complex and varied structures. *N*-Glycans participate in diverse intracellular and extracellular processes, including protein quality control, folding, intracellular trafficking, protein stability, and cell–cell interactions; in addition, they serve as receptors for microbes and viruses^1^. Because the functions of glycans are related to their structures, determination of whole glycans in cells and tissues helps to elucidate biological phenomena in humans and many other organisms.

**Figure 1:**
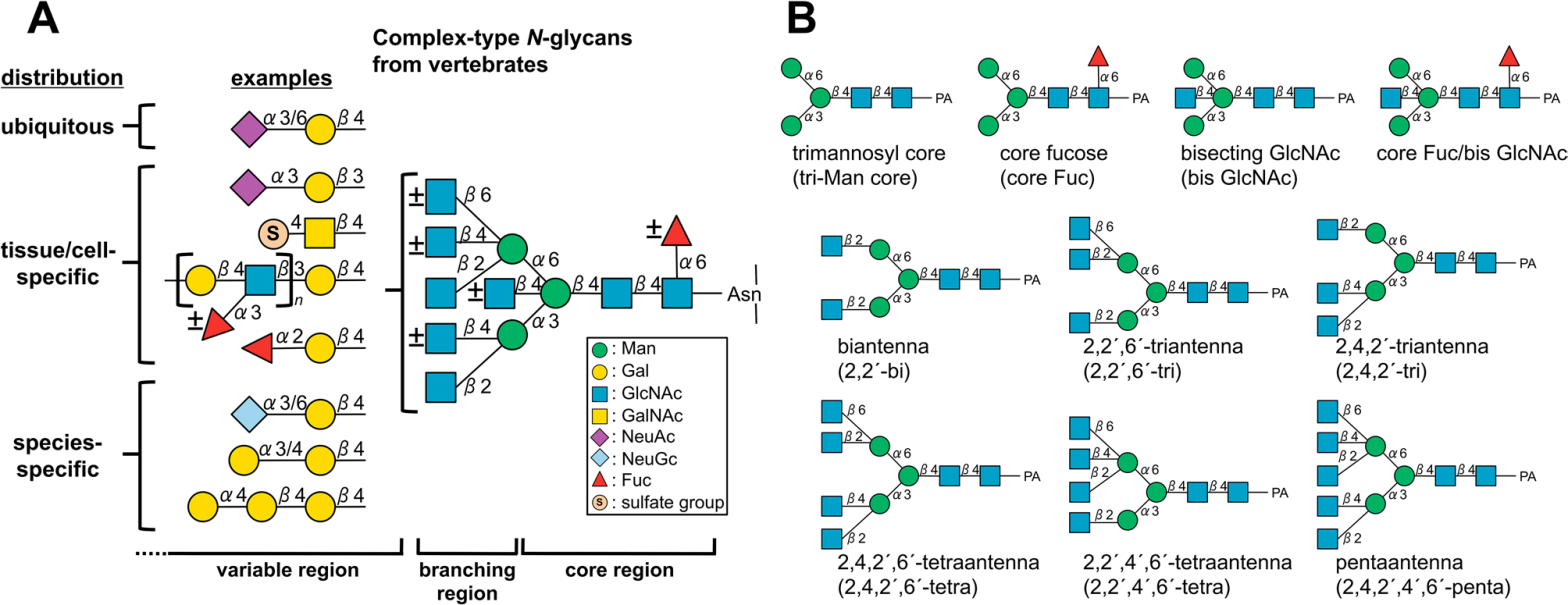
Constant and variable regions of *N*-glycan structures. (**A**) Structural features of complex-type *N*-glycans from vertebrates. In general, an *N*-glycan contains a trimannosyl core structure linked to an Asn residue of a protein; the diversity and microheterogeneity of glycans are generated by incomplete addition of branching GlcNAc, bisecting GlcNAc, core Fuc, and extended branch sequences with numerous variations. The distributions of individual structures vary: some are ubiquitous, whereas others are specific to particular cell types, tissues, or species. The standard Symbol Nomenclature for Glycan system was used for monosaccharide symbols^51^, except for sulfate groups. (**B**) Core and branching structures of PA-*N*-glycans used in this study.

There are three types of *N*-glycans: high mannose-, complex-, and hybrid-type glycans. Complex-type *N*-glycans, which include the most highly complicated structures, can be divided into three domains: core, branching, and variable regions (Fig. 1A). In vertebrate *N*-glycans, the core region has a fundamental trimannosyl core structure covalently attached to an Asn residue of the protein, and is often modified by α1,6-fucosylation on the innermost chitobiose (core Fuc) and/or a bisecting GlcNAc attached to β-Man of the trimannosyl core. Up to five GlcNAc-containing branches linked to α-Man of the trimannosyl core have been detected in vertebrate *N*-glycans (Fig. 1B). Although pentaantennary structures are rare in mammals, they have been reported in some birds^2–4^ and fishes^5,6^. These core structures and branching patterns are thought to influence protein stability and activity^7^. On the other hand, branch sequences extending to non-reducing termini are highly varied, and often differ in a cell-, tissue-, or species-specific manner^8^. These outer parts of glycans are often utilized as receptors by intrinsic and extrinsic carbohydrate-binding proteins^9^. The combinations of core structures with or without modifications, numbers of branches, and various branch sequences, including variable glycosidic linkages accompanied by incomplete extension due to the action of several glycosyltransferases, generate tremendous microheterogeneity. Because glycans consist mainly of several kinds of monosaccharides connected by different linkages, mixtures of glycans from natural sources usually contain several isomers with the same mass values. The presence of diverse isomers makes it difficult to perform complete glycomics by mass spectrometry (MS) alone.

To solve the isomer problem, glycans are often separated by liquid chromatography (LC) using several types of columns^10^, e.g., hydrophilic interaction liquid chromatography (HILIC)^11–13^, reversed-phase chromatography^14–18^, and porous graphitized carbon (PGC) chromatography^19–22^. Glycobioinformatics tools have been developed based on these forms of glycan analysis^23,24^. For HILIC separation, the reducing termini of glycans are often labeled with 2-aminobenzamide (2-AB), 2-aminobenzoic acid (2-AA), or some other fluorescent compound^25^. Separation with a HILIC column depends largely on the number of hydroxyl groups on each glycan, with larger glycans generally eluting later. PGC chromatography in combination with electrospray ionization (ESI)-MS/MS is used to separate and analyze non-labeled or permethylated^21^ glycan mixtures. PGC columns can separate closely related unlabeled glycan isomers that differ only in arm position and sialyl linkages. PGC chips equipped for nanoLC-MS have also been developed for analysis of small amounts of glycan samples^26^. However, evaluating the ability of PGC to separate glycan isomers from various kinds of tissue samples will require accumulation of additional data, as it remains ambiguous in terms of discriminations of branching patterns of multiantennary structures or the number of LacNAc repeats.

Glycans labeled with 2-aminopyridine (PA) can be successfully separated by reversed-phase HPLC using a C18 column^14–16^. Because PA is less hydrophobic than 2-AB or 2-AA, interaction of PA-glycans with a silica-based C18 column are highly dependent on fine differences in glycan structures^17,27^. One of the big differences between isomeric separation of PA-*N*-glycans on a C18 column and other LC methods lies in the relationship between elution rules and the branching patterns of *N*-glycans. In HILIC and PGC separation, galactosylated asialo-*N*-glycans elute in the order bi-, tri-, and tetra-antennary^13,22,28^. Although the two types of triantennary structures, i.e., 2,2′,6′-tri and 2,4,2′-tri (Fig. 1B), are separated clearly on a PGC column^22,28^, both types of fully galactosylated triantennary structures elute between their cognate bi- and tetra-antennary structures. Consequently, it can be difficult to discriminate the branching patterns of these triantennary structures based on their elution positions when they possess several sialic acids. By contrast, in the case of PA-*N*-glycans on a C18 column, 2,2′,6′-tri and 2,4,2′-tri elute much earlier and later, respectively, than the 2,2′-biantennary structure^15,16^. These differences are attributable to the contribution of branching GlcNAc linked to either the C-4 position of α3-Man (β4-GlcNAc) or the C-6 position of α6-Man (β6′-GlcNAc). Given that β6′-GlcNAc and β4-GlcNAc exert opposing effects, 2,4,2′,6′-tetraantennary PA-*N*-glycans that contain both β6′-GlcNAc and β4-GlcNAc elute at positions similar to those of 2,2′-biantennary PA-*N*-glycans. Information about these distinct elution positions based on the branching pattern can be used to determine glycan isomers with different branching structures, even without specific cleavages in MS/MS analysis. Moreover, based on the accumulated data, it is possible to predict glycan structures based on the empirical additivity rule of unit contribution, according to which monosaccharide on PA-glycans contributes either positively or negatively to retention on C18 columns, depending on the type of monosaccharide, position, and glycosidic linkages^16,29^. For instance, addition of core Fuc or bisecting GlcNAc largely contributes positively, and glycans with both core Fuc and bisecting GlcNAc are eluted much later than the non-modified counterparts, in accordance with the combination of positive contributions. Consequently, it is easy to discriminate the core structures based on the elution positions of PA-*N*-glycans.

Despite the extensive advantages of separation and analysis of PA-*N*-glycans on reversed-phase LC, only a few attempts to analyze PA-*N*-glycans from isolated glycoproteins by online LC–MS and MS/MS using a C18 column have been reported^30^. Traditionally, each peak of PA-*N*-glycans separated with a C18 column is detected with a fluorescence detector and fractionated for further analysis with an amide-HILIC column or matrix-assisted laser desorption/ionization (MALDI) time-of-flight (TOF)-MS^31,32^. However, this approach risks overlooking minor glycans, especially for mixtures of glycans prepared from animal tissues.

**Figure 2:**
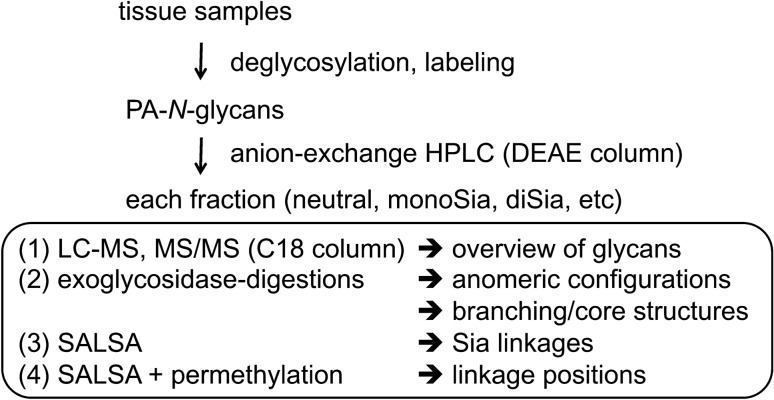
Workflow of procedural steps for structural analysis of *N*-glycans derived from animal tissues. *N*-Glycans are released either chemically or enzymatically, and then labeled with PA. After partial fractionation of PA-*N*-glycans based on their negative charge on an anion-exchange column, each fraction is subjected to LC–MS and MS/MS analysis using a reversed-phase column to obtain an overview of the structural features of tissue glycans. If necessary, glycan structures, including anomeric configurations, are confirmed by a combination of exoglycosidase digestions. The branching/core structures of *N*-glycans can be confirmed based on their elution positions, as well as LC–MS and MS/MS analysis, after exoglycosidase digestions. Linkages of sialic acids on sialylated glycans are determined and quantified by SALSA, followed by LC–MS and MS/MS analysis. Permethylation of alkylamidated glycans followed by LC–MS, MS/MS, and MS^n^ analysis accompanied by cross-ring cleavages is useful for further determination of glycosidic linkage positions in some glycans.

In this study, we first established a method that enables MS analysis of PA-glycans without fractionation using online LC–ESI–MS and MS/MS with a suitable C18 column, which is designed to achieve strong retention of highly polar compounds even in water-rich mobile phases^33^. Using this method, we analyzed *N*-glycans from chicken colon, which are preferentially infected by avian influenza viruses^34^, with the goal of identifying the structures of potential glycan receptors. We confirmed that the unique separation of PA-*N*-glycans was reproducible with an online LC–MS and MS/MS system, and could easily determine core structures and branching pattern of each PA-*N*-glycan. We also showed that the established method could clearly separate glycan isomers with multiantennary structures and/or LacNAc repeats depending on their structures. Taking advantage of these observations, we discovered that the chicken colon express 2,4,2′,4′,6′-pentaantennary structures and 2,2′,4′,6′-tetraantennary structures (Fig. 1B), which are rarely found in mammals. We also used methods for chemical modifications of glycans, such as sialic acid linkage-specific alkylamidation (SALSA) and permethylation, as well as exoglycosidase digestion, to confirm ambiguous branch sequences (Fig. 2). The results revealed that the combined method is a robust approach for analysis of not only glycans from mammals, but also those from other animals, which may contain unknown or unexpected structures.

## Results

The full results, including a detailed structural analysis of glycans from chicken colon are described in *Supplementary Results* along with the Supplementary Figures and Tables. The key results are summarized in this section.

### Overview of *N*-glycans detected by LC–MS and MS/MS analysis

PA-derivatized *N*-glycans from chicken colon were separated into 11 fractions by HPLC using an anion-exchange DEAE column, based on the negative charge of the glycans (Supplementary Fig. S1A). Each fraction was analyzed by LC–MS and MS/MS using a C18 reversed-phase LC column, and PA-glycans were simultaneously monitored with a fluorescence detector (FLD). As reference standards, PA-*N*-glycans from human γ-globulin, bovine fetuin, human α1-acid glycoprotein (α1-AGP), human transferrin, and their enzymatic derivatives (Supplementary Fig. S2) were also analyzed under the same LC–MS and MS/MS conditions. Ten of the eleven fractions (fr. 1, 3–11) from the DEAE column contained PA-*N*-glycans (Supplementary Fig. S1B). Based on the results of full MS and MS/MS analysis, we deduced the monosaccharide compositions and approximate branch sequences of each glycan (Supplementary Table S1), although detailed structural features such as glycosidic linkages and branching patterns remained ambiguous during this initial step. For convenience, deoxyhexoses were indicated as Fuc, unless otherwise noted.

Some complex-type structures were discriminated from their isomeric hybrid-type structures by comparing intensities of B ion fragments at *m/z* 366 (Hex_1_HexNAc_1_) (Supplementary Fig. S3-1). MS and MS/MS analysis also indicated that some PA-*N*-glycans contain Fuc residues on branch positions characterized by B ion fragments at *m/z* 512 (Hex_1_HexNAc_1_Fuc_1_) and *m/z* 803 (Hex_1_HexNAc_1_Fuc_1_NeuAc_1_), LacdiNAc (GalNAc-GlcNAc) sequences characterized by B ion fragments at *m/z* 407 (HexNAc_2_), and LacNAc repeats characterized by B ion fragments at *m/z* 731 (Hex_2_HexNAc_2_) (Supplementary Table S1). The estimated monosaccharide compositions suggested the presence of highly branched structures, such as tri-, tetra-, or pentaantennary *N*-glycans, and these assumptions were confirmed as described in the following sections.

### Determination of anomeric configurations and branching/core structures by exoglycosidase digestions

#### Exoglycosidase digestions

**Figure 3:**
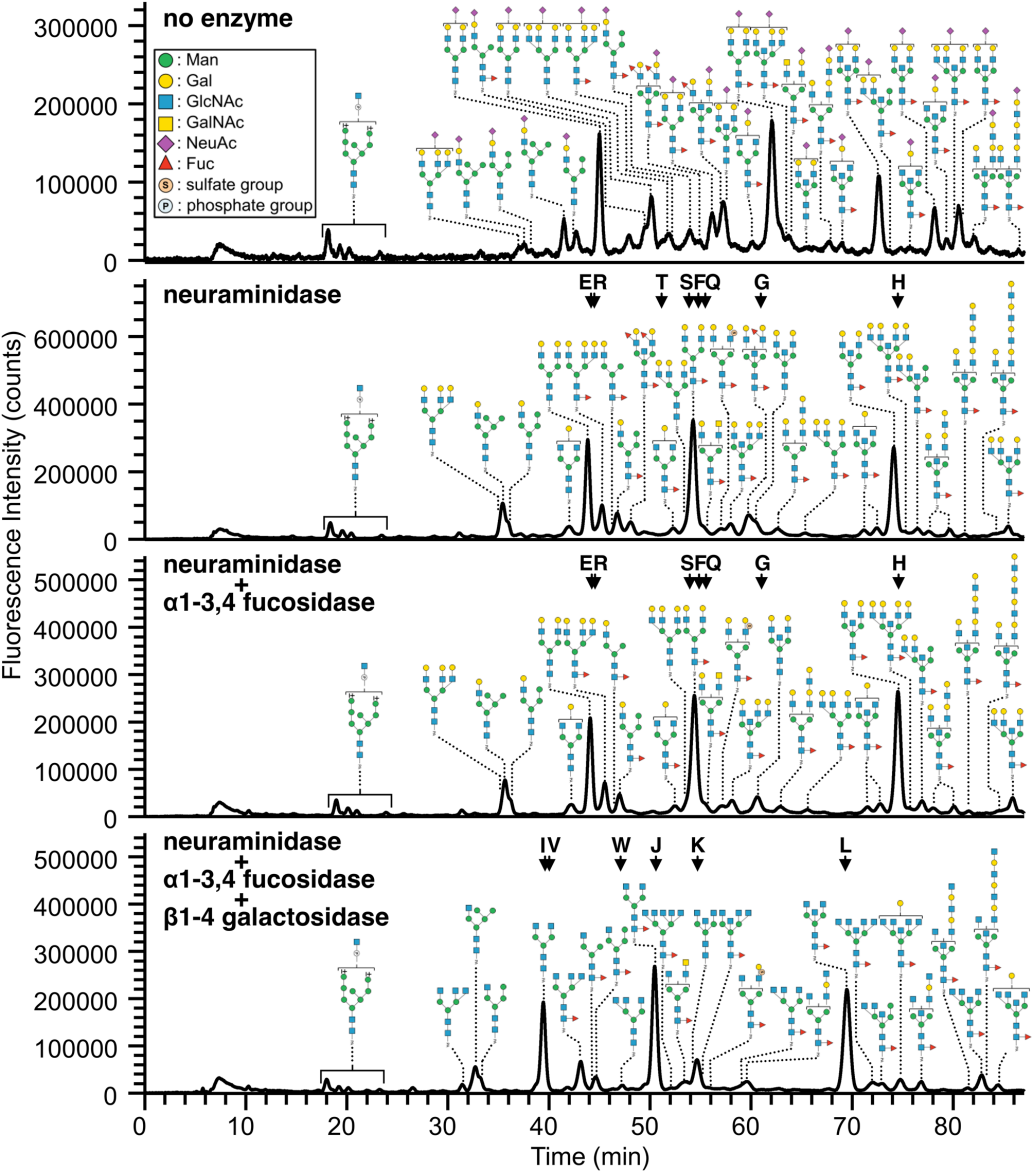
Elution profiles of PA-*N*-glycans from chicken colon after sequential digestions with exoglycosidases. A portion of fr.3 from the DEAE column (monosialylated PA-*N*-glycans, see Supplementary Fig. S2A) was sequentially digested with neuraminidase, α1-3,4 fucosidase, and β1-4 galactosidase, and each digest was subjected to LC–MS and MS/MS analysis. Arrows with alphabetical characters indicate the elution positions of the standard PA-*N*-glycans (Supplementary Fig. S1). Some representative PA-*N*-glycans, but not all the structures detected by MS/MS, are shown in this figure. The standard Symbol Nomenclature for Glycan system was used for monosaccharide symbols^51^, except for sulfate and phosphate groups.

To clarify the sequences of branches and branching patterns of *N*-glycans from chicken colon, each fraction of PA-*N*-glycans was subjected to sequential exoglycosidase digestions using neuraminidase, α1-3,4 fucosidase, and β1-4 galactosidase. After each enzymatic treatment, reaction mixtures were analyzed by LC–MS and MS/MS, similarly to the non-digested samples. Figure 3 shows examples of elution profiles after exoglycosidase digestion for PA-*N*-glycans in fr. 3, which contained monosialylated or monosulfated glycans. The results of LC–MS and MS/MS revealed that NeuAc was removed completely after neuraminidase digestion, and the elution profile of PA-*N*-glycans in fr. 3 was dramatically changed by this treatment. By contrast, the elution profiles after α1-3,4 fucosidase digestion were mostly unchanged, although some peaks shifted by loss of one or two Fuc resides, suggesting the presence of α3/4-Fuc on *N*-glycans. After β1-4 galactosidase digestion, the elution profile changed dramatically again, suggesting that the majority of complex/hybrid-type *N*-glycans possess type II LacNAc (Galβ1-4GlcNAc). However, some minor PA-*N*-glycans retained one LacNAc sequence even after treatment with a sufficient amount of β1-4 galactosidase, implying the presence of type I LacNAc (Galβ1-3GlcNAc) as a minor component.

Next, to find elution rules that would facilitate deduction of glycan structures, we compared the elution positions of some PA-*N*-glycans using LC–MS and MS/MS data as follows:

#### Asialo-biantennary structures with or without core Fuc and/or bisecting GlcNAc

**Figure 4:**
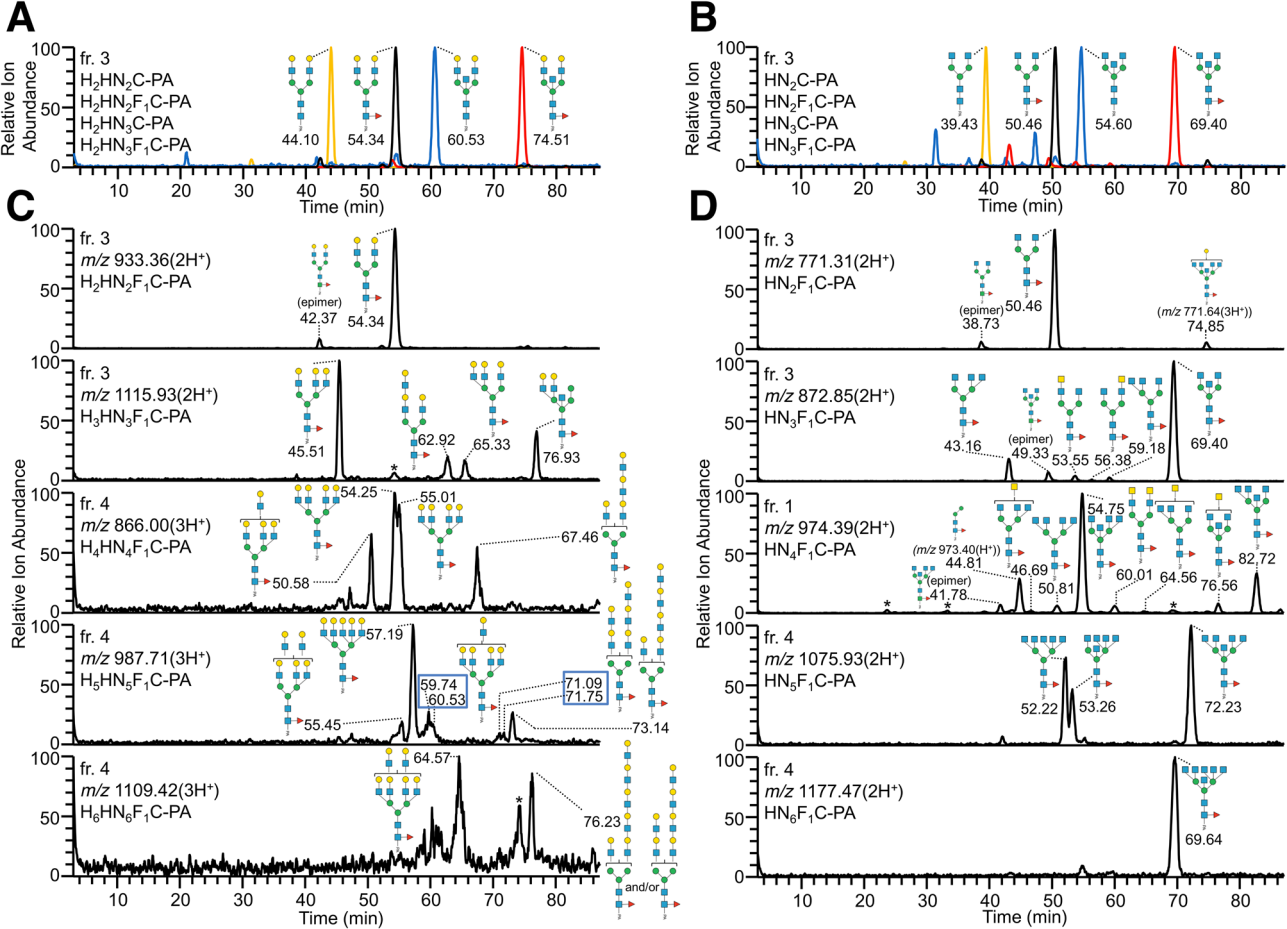
Distinct elution positions of PA-*N*-glycans on reversed-phase LC based on their different core structures and branching patterns. (**A**) EICs at *m/z* 860.33 [Hex_2_HexNAc_2_C-PA(2H^+^), orange line], 933.36 [Hex_2_HexNAc_2_Fuc_1_C-PA(2H^+^), black line], 961.87 [Hex_2_HexNAc_3_C-PA(2H^+^), blue line], and 1034.90 [Hex_2_HexNAc_3_Fuc_1_C-PA(2H^+^), red line] of biantennary PA-*N*-glycans in neuraminidase/α1-3,4 fucosidase–treated fr. 3 from chicken colon. (**B**) EICs at *m/z* 698.28 [HexNAc_2_C-PA(2H^+^), orange line], 771.31 [HexNAc_2_Fuc_1_C-PA(2H^+^), black line], 799.82 [HexNAc_3_C-PA(2H^+^), blue line], and 872.85 [HexNAc_3_Fuc_1_C-PA(2H^+^), red line] of biantennary PA-*N*-glycans in neuraminidase/α1-3,4 fucosidase/β1-4 galactosidase–treated fr. 3. (**C**) EICs at *m/z* 933.36, 1115.93, 866.00, 987.71, and 1109.42 of PA-*N*-glycans in neuraminidase/α1-3,4 fucosidase–treated fr. 3 or fr.4. The peaks indicated by an asterisk (*) are probably artificial ion signals derived from large amounts of PA-*N*-glycans eluted around the corresponding times. (**D**) EICs at *m/z* 771.31, 872.85, 974.39, 1075.93, and 1177.47 of PA-*N*-glycans in neuraminidase/α1-3,4 fucosidase/β1-4 galactosidase–treated fr. 1, 3 or 4.

Elution positions of asialo-biantennary structures with the composition Hex_2_HexNAc_2–3_Fuc_0–1_C-PA (C is the trimannosyl core structure; Man_3_GlcNAc_2_) were detected in extracted ion chromatograms (EICs) of neuraminidase/α1-3,4 fucosidase–treated fr. 3 (Fig. 4A, Supplementary Fig. S3-2A). Comparison of the elution positions of these biantennary PA-*N*-glycans on reversed-phase LC suggested that addition of core Fuc, bisecting GlcNAc, and core Fuc/bisecting GlcNAc made a strong positive contribution to the retention in the ranges 10–11 min, 16–17 min, and 30–31 min, respectively. Similar results were obtained from the EICs of agalactosyl biantennary structures with the composition HexNAc_2–3_Fuc_0–1_C-PA from neuraminidase/α1-3,4 fucosidase/β1-4 galactosidase–treated fr. 3 (Fig. 4B, Supplementary Fig. S3-6A). These empirical additivity rules are consistent with previous reports describing separation of PA-labeled glycans with C18 columns^16,29^.

#### Monosialylated biantennary structures

Elution positions of monosialylated biantennary structures with the composition Hex_2_HexNAc_2–3_Fuc_0–1_NeuAc_1_C-PA, were detected in EICs of fr. 3 without exoglycosidase digestions (Supplementary Fig. S3-2B). Linkages and positions of NeuAc on biantennary branches were deduced by comparing the elution positions, full MS, and MS/MS spectra of these PA-*N*-glycans with those of monosialylated standards (Standard a–h in Supplementary Fig. S2). Some of the PA-*N*-glycans from chicken colon did not match any standards we used in terms of these three criteria. In those cases, we deduced their structures based on the three criteria, as well as the results of MS analysis with SALSA (Supplementary Table S2), as described in the next section. The results indicated that the addition of α2,3-NeuAc contributed positively in the range of 6–8 min (without bisecting GlcNAc, with or without core Fuc), 4–10 min (with bisecting GlcNAc, without core Fuc), or 3–6 min (with bisecting GlcNAc, with core Fuc), whereas the addition of α2,6-NeuAc attached to LacNAc on α3-Man made a smaller contribution to retention: less positive (0–2 min, without bisecting GlcNAc, with or without core Fuc) or even negative (1–4 min, with bisecting GlcNAc, with or without core Fuc).

#### Fucosylated biantennary structures

Using EICs of neuraminidase–treated fr. 3, we compared the elution positions of fucosylated biantennary structures with bisecting GlcNAc assigned as Hex_2_HexNAc_3_Fuc_1–3_C-PA (Supplementary Fig. S3-3A). The results indicated that the addition of one core Fuc made a strong positive contribution to retention (13–14 min). By contrast, the addition of one α1,3-Fuc residue on a branch made a strong negative contribution to retention (10–15 min).

#### Sulfated biantennary structures

Several minor PA-*N*-glycans from chicken colon possessed one or two sulfate groups. For example, sulfated biantennary PA-*N*-glycans, assigned as Hex_2_HexNAc_2_Fuc_1_NeuAc_0–2_(SO_3_)_1_C-PA, were detected in fr. 3, 6, 7, and 10. EICs of these sulfated PA-*N*-glycans (Supplementary Fig. S3-4A) indicated the presence of several isomers that differed in terms of the linkages and positions of NeuAc. The linkages and positions of NeuAc, as well as the position of the sulfate group, were determined by SALSA and permethylation, as described below. In the case of non-sialylated biantennary PA-*N*-glycans with core Fuc without bisecting GlcNAc, the sulfate group positively contributed to retention in the range of 2–3 min. By contrast, the group contributed negatively for the glycans with one α2,6-NeuAc or α2,3-NeuAc (0–4 min), two α2,6-NeuAc (2–3 min), or two α2,3-NeuAc (6–7 min). These observations indicate that the positive contribution of addition of NeuAc to biantennary PA-*N*-glycans without bisecting GlcNAc was abolished by the addition of a sulfate group.

#### Triantennary structures

EICs at *m/z* 1042.90, 1115.93, 1144.44, and 1217.47 of PA-*N*-glycans in neuraminidase/α1-3,4 fucosidase–treated fr. 3, assigned as Hex_3_HexNAc_3–4_Fuc_0–1_C-PA, exhibited several isomer peaks (Supplementary Fig. S3-5A). According to the empirical rules governing the elution positions of PA-*N*-glycans on reversed-phase LC with a C18 column, PA-*N*-glycans with a 2,2′,6′-triantennary structure and a 2,4,2′-triantennary structure (Fig. 1B) generally elute earlier and later, respectively, than those with a cognate 2,2′-biantennary structure^16,29^. As described in the *Introduction*, these differences are attributable to the contribution of branching GlcNAc linked to either the C-4 position of α3-Man or the C-6 position of α6-Man. We found that this empirical rule was also applicable to our new system (Fig. 4C). Notably, PA-*N*-glycans with the composition Hex_3_HexNAc_3_Fuc_1_C-PA that eluted around 62.92 min and those with the composition Hex_3_HexNAc_4_Fuc_1_C-PA that eluted around 79.85 min had a LacNAc repeat sequence (Gal-GlcNAc-Gal-GlcNAc, LacNAc_2_). The MS/MS spectra of these glycans contained strong signals of the hallmark B ion fragments at *m/z* 731 (Hex_2_HexNAc_2_) and the Y ion fragments of [precursor−Hex_2_HexNAc_2_](H^+^) (Supplementary Fig. S3-5B).

#### LacdiNAc and branching patterns

To compare the elution positions among PA-*N*-glycans with different branching patterns, we used EICs of chicken colon PA-*N*-glycans with several compositions (HexNAc_2–6_Fuc_0–1_C-PA) prepared by neuraminidase/α1-3,4 fucosidase/β1-4 galactosidase digestions (Fig. 4D, Supplementary Figs. S3-6A, S3-7A, S3-8A). The structures of glycan isomers at each peak were deduced based on the elution position, full MS, and MS/MS as described in *Supplementary Results*. The presence of LacdiNAc (GalNAc-GlcNAc) was confirmed by the hallmark B ion fragments at *m/z* 407 (Supplementary Figs. S3-6B, S3-7B). The data imply that addition of the second HexNAc (most likely GalNAc) to the first HexNAc (most likely GlcNAc) of HexdiNAc, resulted in a positive contribution to retention in the range of 3–6 min, and that this contribution differed slightly depending on the arm where the HexNAc was added. This moderate contribution of GalNAc is similar to the effect of addition of β4-GalNAc to GlcNAc on LacdiNAc branches as previously reported^29,35^.

Comparison of the reversed-phase LC elution positions of agalactosyl 2,2′,6′- and 2,4,2′-triantennary PA-*N*-glycans to those of cognate biantennary PA-*N*-glycans with or without core Fuc but lacking bisecting GlcNAc indicated that addition of β6′-GlcNAc or β4-GlcNAc made a negative (7–8 min) or positive (7–9 min) contribution to retention, respectively (Fig. 4D, Supplementary Fig. S3-6A), similar to those of fully galactosylated asialo triantennary PA-*N*-glycans (Fig. 4C). Given that β6′-GlcNAc and β4-GlcNAc exerted opposing effects, 2,4,2′,6′-tetraantennary PA-*N*-glycans, that contain both β6′-GlcNAc and β4-GlcNAc eluted at positions similar to those of 2,2′-biantennary PA-*N*-glycans. These empirical additivity rules were consistent among PA-labeled *N*-glycans separated with C18 columns^16,29^. In PA-*N*-glycans with bisecting GlcNAc, the negative and positive contributions of β6′-GlcNAc and β4-GlcNAc were similar but stronger (11–15 min and 13–18 min, respectively) than those of PA-*N*-glycans without bisecting GlcNAc (Fig. 4D, Supplementary Fig. S3-6A).

We found that PA-*N*-glycans with the composition HexNAc_5_Fuc_1_C-PA that eluted around 52.22, 53.26, or 72.23 min (Fig. 4D, Supplementary Fig. S3-8A) could have been 2,4,2′,4′,6′-penta with core Fuc, 2,2′,4′,6′-tetra with core Fuc and bisecting GlcNAc, or 2,4,2′,6′-tetra with core Fuc and bisecting GlcNAc, respectively, as described in *Supplementary Results*. Similarly, the PA-*N*-glycan with composition HexNAc_6_Fuc_1_C-PA that eluted around 69.64 min could have been 2,4,2′,4′,6′-penta with bisecting GlcNAc and core Fuc. It should be noted that both 2,4,2′,4′,6′-pentaantennary structures and 2,2′,4′,6′-tetraantennary structures are rarely found in mammals.

#### LacNAc repeats and multiantennary structures with LacNAc

Compositions of the complex-type PA-*N*-glycans from chicken colon were mainly Hex_*n*_HexNAc_*n*_Fuc_0–1_NeuAc_0–5_C-PA or Hex_*n*_HexNAc_(*n*+1)_Fuc_0–1_NeuAc_0–4_C-PA (*n* = 2–5), with some exceptions, suggesting the presence of multiantennary structures, extended LacNAc repeat sequences, or both. To confirm the presence of these structures, we examined the EICs of PA-*N*-glycans with the compositions of Hex_*n*_HexNAc_*n*_Fuc_0–1_C-PA(3H^+^) or Hex_*n*_HexNAc_(*n*+1)_Fuc_0–1_C-PA(3H^+^) (*n* = 4–6) using data from LC–MS and MS/MS analysis of each neuraminidase/α1-3,4 fucosidase–digested fraction (Fig. 4C, Supplementary Figs. S3-9, S3-11, S3-13, S3-15). We also examined the EICs of their β1-4 galactosidase–digested products (Supplementary Figs. S3-10, S3-12, S3-14, S-15) to confirm the number of remaining Hex (Gal) residues. The results indicated that PA-*N*-glycans with multiantennary structures (including pentaantennarry structures) and/or LacNAc repeats could be successfully separated based on the branching patterns and the number of LacNAc repeats. For example, EICs at *m/z* 987.71 [Hex_5_HexNAc_5_Fuc_1_C-PA(3H^+^)] of PA-*N*-glycans revealed that PA-*N*-glycans with this composition were clearly separated into fully galactosylated 2,2′-bi-, 2,2′,6′-tri-, and 2,4,2′,4′-tetraantennary structures with LacNAc repeats as well as a 2,4,2′,4′,6′-pentaantennary structure (Fig. 4C, Supplementary Fig. S3-13). Notably, we detected both 2,4,2′,6′-tetra with four type II LacNAc (Galβ1-4GlcNAc), that eluted slightly earlier than the other, and 2,4,2′,6′-tetra with three type II LacNAc and one type I LacNAc (Galβ1-3GlcNAc) as shown in Fig. 4C, Supplementary Figs. S3-9, S3-11. Moreover, we newly identified 2,2′,4′,6′-tetra with four type II LacNAc with bisecting GlcNAc and core Fuc, which are rarely found in mammals (Supplementary Fig. S3-11).

### Semi-quantitative analysis of Sia-linkages by SALSA

**Figure 5:**
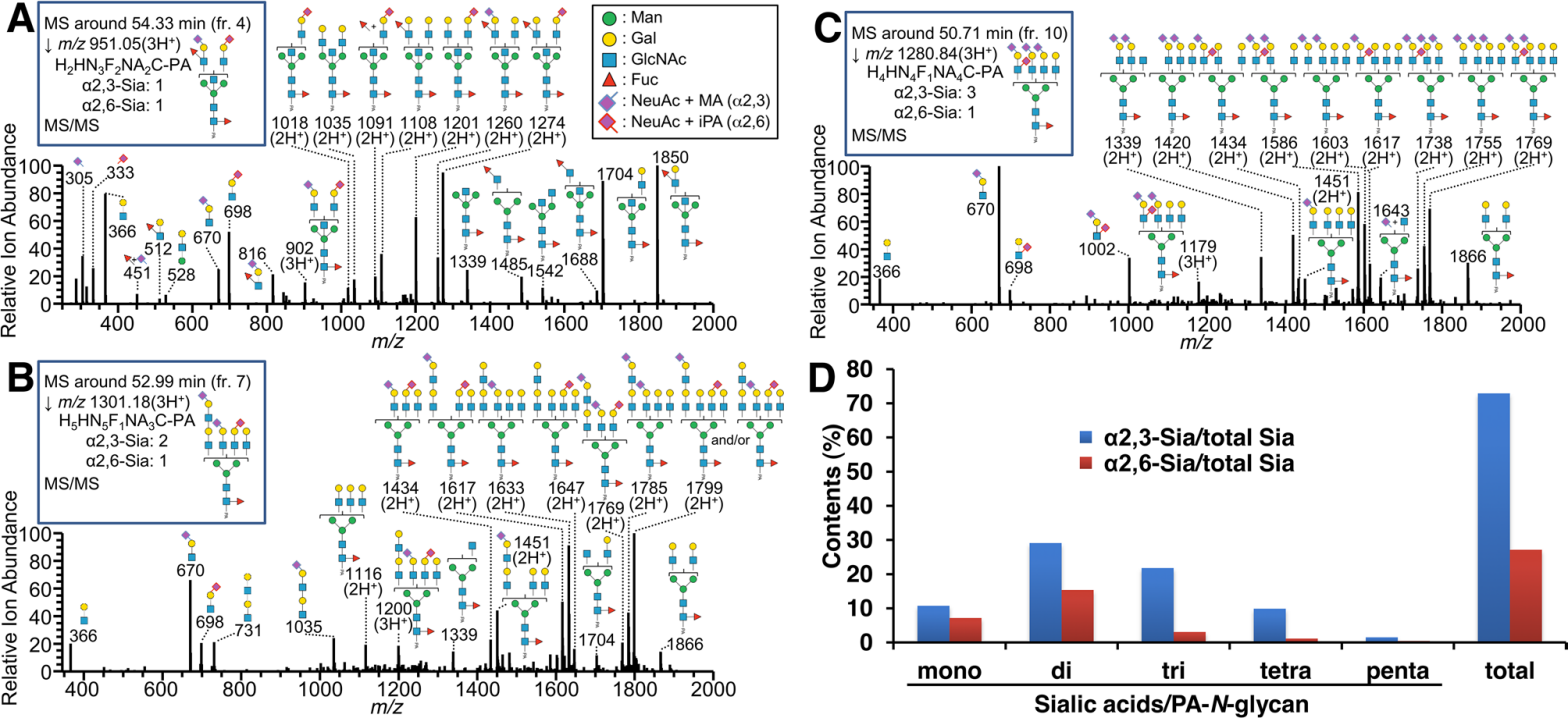
Analysis of Sia-linkages of PA-*N*-glycans from chicken colon using the SALSA method. (**A**–**C**) Examples of MS/MS spectra of alkylamidated PA-*N*-glycans from chicken colon. Structures of fragments shown in the figures are representative examples, and other isomeric ions can also be generated. (**D**) Relative content of α2,3- and α2,6-Sia in sialylated *N*-glycans from chicken colon, estimated using the SALSA method. Proportions of α2,3- and α2,6-Sia in mono-, di-, tri-, tetra-, or penta-sialylated glycans, as well as in total glycans from the tissue, were calculated based on the results of LC–MS and MS/MS analysis of PA-*N*-glycans after SALSA, as indicated in Supplementary Table S2.

To discriminate α2,3- or α2,6-Sia in PA-*N*-glycans from chicken colon, we chemically modified a portion of each fraction containing sialylated PA-*N*-glycans, i.e., fr. 3–11, by SALSA, and then analyzed the sample by LC–MS and MS/MS. The elution profiles of each fraction are shown in Supplementary Fig. S4. Based on the results of full MS and MS/MS analyses, we deduced the monosaccharide compositions and Sia-linkages of each PA-*N*-glycan detected by FLD (Supplementary Table S2). Using the SALSA method, α2,3-Sia and α2,6-Sia were alkylamidated by methylamine (MA, + 13.032) and isopropylamine (iPA, + 41.063), respectively, resulting in a mass difference (Δ = 28.031)^36^. The MS/MS spectra of the alkylamidated PA-*N*-glycans revealed structural features of sialylated branches via their characteristic B ion signals. For instance, MS/MS spectra at *m/z* 951.05, whose composition is Hex_2_HexNAc_3_Fuc_2_(NeuAc + MA)_1_(NeuAc + iPA)_1_C-PA(3H^+^), contained B ion fragments at *m/z* 670 [(NeuAc + MA)_1_Hex_1_HexNAc_1_], 698 [(NeuAc + iPA)_1_Hex_1_HexNAc_1_], and 816 [(NeuAc + MA)_1_Hex_1_HexNAc_1_Fuc_1_], suggesting that fucosylated branches possess α2,3-Sia (Fig. 5A) such as sialyl Le^x^ [sLe^x^, NeuAcα2-3Galβ1-4(Fucα1-3)GlcNAc] or sialyl Le^a^ [sLe^a^, NeuAcα2-3Galβ1-3(Fucα1-4)GlcNAc]. Some PA-*N*-glycans from chicken colon possess sialylated LacNAc repeat structures. B ion fragments of this branch sequence were　detected at *m/z* 1035 [(NeuAc + MA)_1_Hex_2_HexNAc_2_], suggesting that the LacNAc repeats were α2,3-sialylated (Fig. 5B). We also found that some PA-*N*-glycans generated B ion fragments at *m/z* 1002 [(NeuAc + MA)_1_(NeuAc + iPA)_1_Hex_1_HexNAc_1_], suggesting the presence of one α2,3-Sia and one α2,6-Sia on the same LacNAc branch (Fig. 5C). This sequence is presumably NeuAcα2-3Galβ1-3(NeuAcα2-6)GlcNAc, as found in bovine fetuin *N*-glycans, although we could not confirm the structure. We detected the compositions Hex_2_HexNAc_2_Fuc_1_NeuAc_0–1_NeuGc_1_C-PA (pk. 3–14-1 and pk. 5–5-2 in Supplementary Table S2), which are likely to be PA-*N*-glycans with one NeuGc. Because we detected only trace amounts of NeuGc-containing glycans, these NeuGc residues may be derived from the diet but not biosynthesized by the chickens, as suggested previously^37^.

We estimated the proportions of α2,3- and α2,6-Sia at non-reducing termini of PA-*N*-glycans from chicken colon using the peak area of each PA-*N*-glycan derivatized by the SALSA method (Fig. 5D). The results revealed that the proportions of α2,3- and α2,6-Sia in sialylated branches of PA-*N*-glycans were 72.9% and 27.1%, respectively. It should be noted that the proportions of α2,3-Sia on mono- (10.6%), di- (29.1%), tri- (21.8%), tetra- (9.9%), and penta- (1.5%) sialylated PA-*N*-glycans in chicken colon were always higher than the corresponding proportions of α2,6-Sia, regardless of the number of sialylations per PA-*N*-glycan.

### Determination of linkage positions by SALSA/permethylation

**Figure 6:**
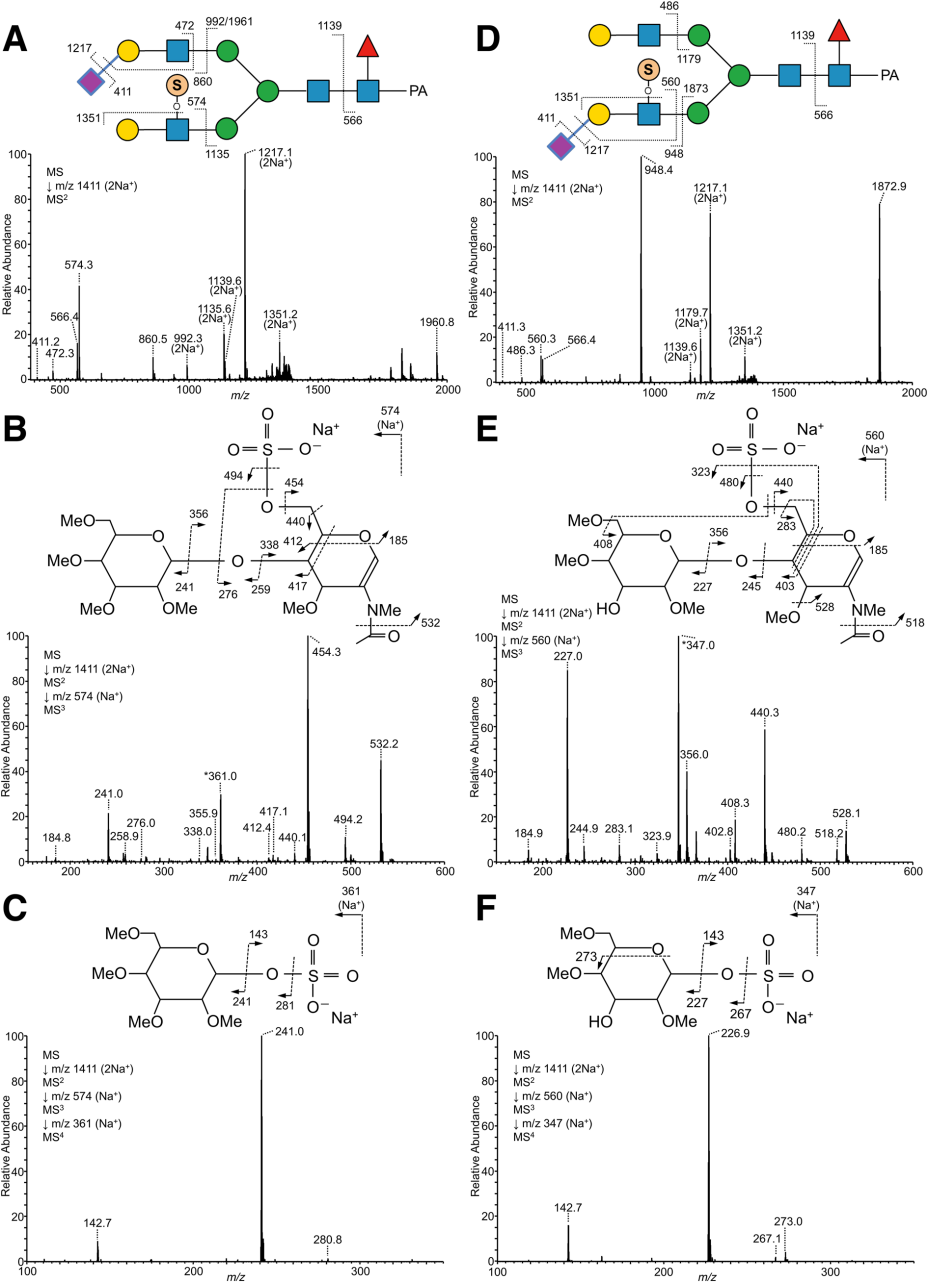
MS^n^ analysis of alkylamidated and permethylated PA-*N*-glycans with sulfation derived from chicken colon. (**A**) MS^2^ spectrum of α2,3-sialylated PA-*N*-glycan with sulfated LacNAc from doubly sodiated precursor ion at *m/z* 1411, corresponding to Hex_2_HexNAc_2_Fuc_1_(NeuAc + MA)_1_(SO_3_-H + Na)_1_C-PA, eluted around 42.01 min on reversed-phase LC. (**B**) MS^3^ spectrum of B ion fragments Hex_1_HexNAc_1_(SO_3_-H + Na)_1_ at *m/z* 574. The peak at *m/z* 361 indicated by the asterisk (*) was probably generated by rearrangement of sodium sulfate (–SO_4_Na) on HexNAc to the B ion fragments at *m/z* 241, derived from Hex on the non-reducing end, according to the result of MS^4^ analysis at *m/z* 361 (**C**). (**D**) The MS^2^ spectrum of PA-*N*-glycan with sulfated α2,3-sialyl LacNAc from doubly sodiated precursor ion at *m/z* 1411, corresponding to Hex_2_HexNAc_2_Fuc_1_(NeuAc+ MA)_1_(SO_3_-H + Na)_1_C-PA, eluted around 43.61 min on reversed-phase LC. (**E**) MS^3^ spectrum of the B/Y ion fragments Hex_1_HexNAc_1_(SO_3_–H + Na)_1_ at *m/z* 560. The peak at *m/z* 347 indicated by the asterisk (*) is probably generated by rearrangement of sodium sulfate (–SO_4_Na) on HexNAc to the B/Y ion fragments at *m/z* 227 derived from Hex on the non-reducing end, according to the result of MS^4^ analysis at *m/z* 347 (**F**). Although the sulfate groups on the glycan structures are represented as if they were located on a branch linked to α3-Man (**A**, **D**), the actual branch positions have not been determined.

Although α2,3- and α2,6-Sia-linkages on glycans can be discriminated by the SALSA method, and the major glycosidic linkages of PA-*N*-glycans from chicken colon could be determined by exoglycosidase digestions as described above, the accurate glycan sequences on branches of some glycans remained to be determined. The B ion fragments generated in MS/MS analysis are useful for deducing branch compositions, but ion rearrangements of saccharides and functional groups often yield misleading results^38^. To solve this problem, permethylation of glycans is preferable, as this derivatization can suppress rearrangements on MS^n^ analysis and is useful for determining glycosidic linkages by cross-ring cleavages. We recently established a combined method in which the glycans are permethylated after SALSA^39,40^. In this study, a portion of each fraction of chicken colon PA-*N*-glycans separated on a DEAE column was subjected to SALSA/permethylation and analyzed by LC–MS, MS/MS, and MS^n^. Using this method, α2,3-/α2,6-Sia-linkages and positions of other glycan modifications can be determined simultaneously. For example, we detected three isomers of PA-*N*-glycans with the composition Hex_2_HexNAc_2_Fuc_1_NeuAc_1_(SO_3_)_1_C-PA, which possess one sulfate group and one Sia, as determined by LC–MS and MS/MS analysis (pk. 6–9-1, pk. 7–3-1, and pk. 7–5-1 in Supplementary Table S1; at *m/z* 1118.89 of fr. 6 and fr. 7 in Supplementary Fig. S3-4A, B). It was initially unclear whether the sulfate group and Sia are on the same or different branches, as the sulfate group is easily transferred to different positions under ionization conditions. By contrast, using the combined SALSA/permethylation method and MS^n^ analysis, we could determine the linkages of Sia and the position of the sulfate group as described in *Supplementary Results* (Fig. 6). We also confirmed the branch sequences of Le^x^ (Supplementary Fig. S5-1), sLe^x^ (Supplementary Fig. S5-2), LacdiNAc (Supplementary Fig. S5-3), and sLacNAc repeat (Supplementary Fig. S5-4, S5-5) using this combined method.

### Summary of the structural features of *N*-glycans in chicken colon

**Figure 7:**
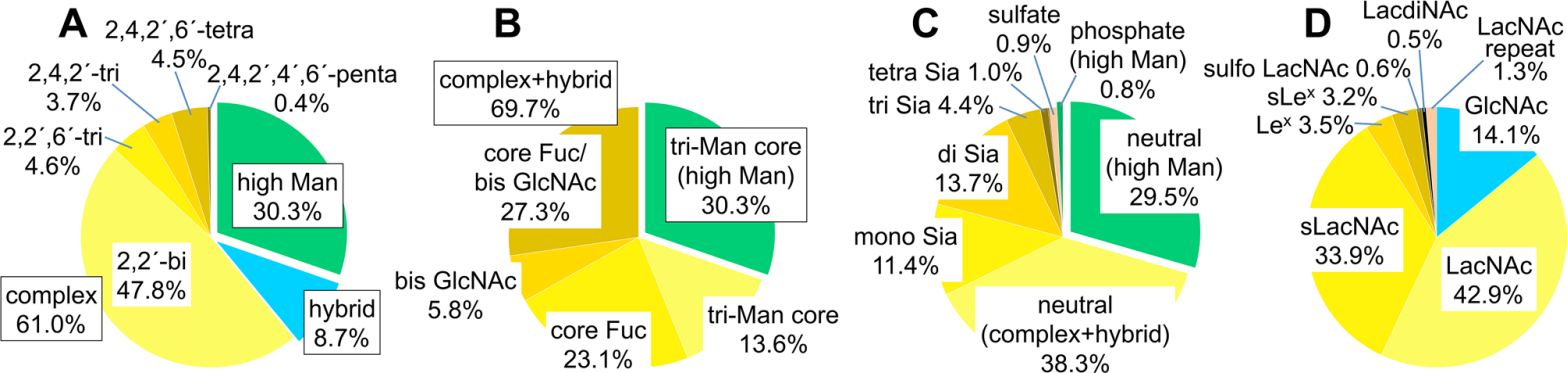
Summary of structural features of *N*-glycans from chicken colon. (**A**) Relative abundances of high mannose, hybrid, and complex-type *N*-glycans categorized by branching patterns (Fig. 1B). (**B**) Relative abundances of *N*-glycans, categorized by core structures (Fig. 1B). (**C**) Relative abundances of neutral, sialylated, sulfated, and phosphorylated *N*-glycans in chicken colon. The sulfated glycan group includes those with simultaneously sialylated glycans, whereas the sialylated glycan groups do not include any sulfated glycans. Each value (%) in *A*–*C* are relative to the total amount of all PA-*N*-glycans (= 100%) in Supplementary Table S1 (**D**) Relative abundances of characteristic branch sequences on complex or hybrid-type glycans, relative to the total amount of GlcNAc/LacNAc/LacdiNAc-containing branches (= 100%) in all PA-*N*-glycans in Supplementary Table S1. Each group of GlcNAc (excluding bisecting GlcNAc), LacNAc, sialyl LacNAc (sLacNAc), Le^x^, and sLe^x^ includes the corresponding branches located at the non-reducing termini of complex and hybrid-type *N*-glycans. Each group of sulfated LacNAc (sulfo LacNAc), LacdiNAc, and LacNAc repeats includes both sialylated and non-sialylated branch sequences.

Based on the results of LC–MS, MS/MS, exoglycosidase digestions, SALSA, and SALSA/permethylation, we deduced the structures of almost all major PA-*N*-glycans from chicken colon, including the core structures, branching patterns, and branch sequences, with the exception of ambiguous positions of asymmetric branches, Sia-linkages, and LacNAc linkages (i.e., type I or type II) on each glycan. The deduced structures are summarized in Supplementary Table S1, along with the relative amounts calculated from the area of each peak detected by fluorescence and full MS. Using the data sets, we calculated the contents of categorized glycan structures (Fig. 7). The details are described in *Supplementary Results*. To quantify the structural features of branch sequences, we calculated the amounts of each GlcNAc/LacNAc/LacdiNAc-containing branch on complex and hybrid-type *N*-glycans (Fig. 7D). Although more than 90% of the branches were simple GlcNAc, LacNAc, or sialyl LacNAc (sLacNAc) sequences, some minor sequences such as Le^x^, sLe^x^, sulfated LacNAc, LacdiNAc, and LacNAc repeat were characteristic structures of chicken colon *N*-glycans.

## Discussion

In this study, we first confirmed that PA-*N*-glycans could be clearly separated based on their core structures and branching patterns using a C18 column adopted for water-rich mobile phases. This system allowed the eluted PA-glycans to be directly analyzed with an online LC–MS and MS/MS system. The resultant elution patterns were similar to those obtained by conventional methods, using mobile phases containing 1-butanol^15,16^, regardless of differences in end capping of the columns. We used the water–acetonitrile–formic acid-based mobile phase, because it is well known to have excellent ionization efficiency, and is suitable for use in ESI–MS analysis. The addition of 0.2% formic acid in the mobile phase enhanced the ionization of PA-labeled glycans. Thus, it became possible to avoid further time-consuming fractionation of each glycan from a tissue sample using several different types of columns for further analyses. For example, a similar approach with PA-labeled *N*-glycans has been used to study mouse cerebral cortex^41^, but not using LC–MS, MS/MS. In their study, the mixtures of PA-*N*-glycans from the tissue were separated with three kinds of columns, i.e., an anion-exchange, normal phase, and reversed-phase columns, prior to analyze with MALDI-TOF–MS. Moreover, they analyzed Sia-linkages quantitatively using an α2,3-sialidase and affinity chromatography with SNA lectin, which binds to α2,6-Sia. By contrast, because we used SALSA and SALSA/permethylation methods to determine Sia-linkages and the positions of Sia on branches, the results are chemically clearer.

Because of the unique unit contribution of PA-*N*-glycans on a C18 column, it is easy to discriminate between two types of triantennary structures, 2,2′,6′-tri and 2,4,2′-tri (Fig. 1B, Fig. 4, Supplementary Figs. S3-5, S3-6) based on their elution positions. This distinct separation pattern has not been reported for PGC or HILIC columns^13,22,28^. Although these two types of triantennary structures are usually not carefully discriminated by MS analysis, they are biosynthesized by the action of different enzymes, i.e., *N*-acetylglucosaminyltransferases V and IV, and are likely to have differently effects on biological phenomena. For example, rabbit asialoglycoprotein receptor preferentially binds to terminal β4-Gal residues on 2,4,2′-tri rather than on 2,2′,6′-tri^42,43^. Because the conformations of these two types of triantennary structures are different, they may confer different effects on the stability of glycoproteins to which they are attached. Therefore, discrimination of these structures is important for accurate determination of tissue glycomes.

**Figure 8:**
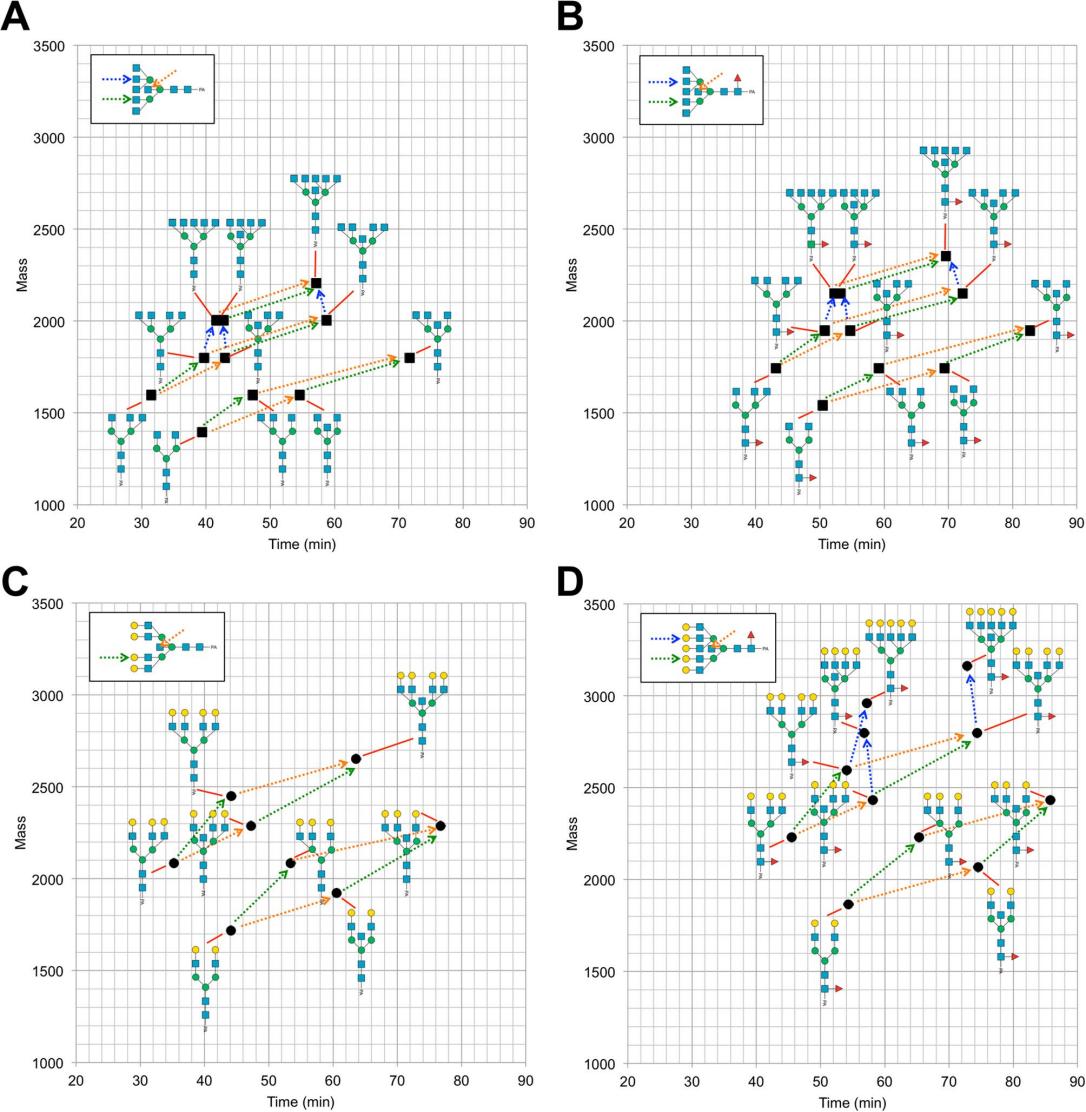
Two-dimensional (2D) maps of PA-*N*-glycans with different branching patterns and/or core structures. The elution time (*x*-axis) and the mass value (*y*-axis) of each PA-*N*-glycan were plotted. (**A**, **B**) 2D maps of agalacto-PA-*N*-glycans (solid squares) with (**B**) or without (**A**) core Fuc. (**C**, **D**) 2D maps of asialo-PA-*N*-glycans (solid circles) with type II LacNAc branches with (**D**) or without (**C**) core Fuc. Dotted line arrows indicate shifts of the elution positions and mass values of PA-*N*-glycans by the addition of branching GlcNAc/LacNAc on 4- (green line) or 4′-position (blue line) of core α-Man residues, or by the addition of bisecting GlcNAc (orange line).

The elution rules of agalacto- or asialo-PA-*N*-glycans separated on the C18 column were summarized on two-dimensional (2D) maps (Fig. 8), which shows plots of elution times (*x*-axis) and mass values (*y*-axis) of several PA-*N*-glycans with different branching patterns and core structures. The figure indicates differences of elution times by the addition of branches and bisecting GlcNAc. Comparing two panels (A and B, or C and D), it is obvious the parallel shifts of each PA-*N*-glycans by the addition of core Fuc. Additional 2D maps based on different branch sequences will be available in the future when more data are accumulated.

Once the elution positions of each simple PA-*N*-glycan with only a core region and branching GlcNAc were determined, the unit contributions of each monosaccharide on extended branches could be estimated by comparing the elution positions of standard PA-*N*-glycans. Based on the unit contributions, branch sequences could be deduced by both MS/MS spectra and elution positions. Because MS/MS analysis alone may cause misidentification due to rearrangements of fragment ions, structures can be more reliably determined using both factors. If it is necessary to determine branch sequences in more detail, including glycosidic linkages, one can chemically modify the PA-*N*-glycans using the SALSA/permethylation method. Although this process is optional, chemical modification and MS^n^ analysis are useful for confirmation of glycan structures derived from animal tissue samples that may contain unknown and unexpected structures.

Avian influenza viruses bind α2,3-Sia–containing glycans on host cells via the spike membrane glycoprotein hemagglutinin (HA)^44^. To identify the actual glycan structures used as HA receptors, we initially analyzed *N*-glycan structures from several chicken tissues. We expected that chickens would express simpler glycans than other avian species, because unlike many other birds, chickens do not express both Galα1-4Gal and Galβ1-4Gal at non-reducing termini^8,45,46^. Although we confirmed the absence of these glycan epitopes in chicken colon, determination of glycan structures was still complicated by the presence of various isomers. Nevertheless, using the advantages of unique separations of PA-*N*-glycans on a C18 column, we could clearly demonstrate that the complexity was generated by the presence of various multiantennary structures, including pentaantennary structures and LacNAc repeat–containing structures, as well as Le^x^, sLe^x^, sulfo sialyl LacNAc, and LacdiNAc. Because both multiantennary structures and extended LacNAc repeat–containing structures with α2,3-Sia could influence interactions with avian influenza viruses, our data will be useful for determining the target structures on avian tissues.

## Materials and methods

### Materials

Tissues from 3-week-old male chickens were a kind gift from Dr. Toshie Sugiyama of Niigata University. Trypsin and chymotrypsin were purchased from Sigma-Aldrich (St. Louis, MO, USA), and guanidine hydrochloride, iodoacetamide, and dithiothreitol were purchased from Fujifilm Wako Pure Chemical Co. (Osaka, Japan). Recombinant glycoamidase F from *Flavobacterium meningosepticum* (GAF, aka *N*-glycosidase F, PNGase F) was purchased from Roche (Mannheim, Germany). Neuraminidase (α2-3,6,8,9 neuraminidase) from *Arthrobacter ureafacience* was purchased from Nacalai Tesque (Kyoto, Japan). α1-3,4 Fucosidase from the sweet almond tree and β1-4 galactosidase S from *Streptococcus pneumonia* were purchased from New England BioLabs (Ipswich, MA, USA). Other materials including reagents, columns for LC, and PA*-N*-glycans from human γ-globulin, α1-AGP, and bovine fetuin were obtained as described previously^39,40^. α2,6-Monosialylated PA-*N*-glycans were prepared from human transferrin and human γ-globulin, and α2,3-monosialylated PA-*N*-glycans were prepared by treatment of asialo-biantennary PA-*N*-glycans with recombinant α2,3-sialyltransferase from *Photobacterium phosphoreum*^39^.

### Preparation of PA-N-glycans from tissue samples

Isolated chicken colon/rectum were washed several times with PBS, immediately frozen in liquid nitrogen, and kept at − 70 °C until use. Tissues (100–200 mg, wet weight) were homogenized with a Polytron homogenizer, and suspended with 4 volumes of cold water (estimated using the wet weight of the tissue). To extract lipids, 2.67 volumes of methanol and then 1.33 volumes of chloroform were mixed into the suspension. After centrifugation, the supernatant was removed. The residual pellets were air-dried and resuspended in 4 volumes of 6 M guanidine-HCl in 50 mM Tris–HCl (pH 8.4). The tissue suspensions were mixed with 0.5 volumes of 240 mM dithiothreitol in 6 M guanidine-HCl and incubated at 37 °C for 1 h. For alkylation of thiols, the suspensions were mixed with 0.5 volumes of 600 mM iodoacetamide in 6 M guanidine-HCl, incubated at room temperature for 1 h in the dark, and then dialyzed against deionized water. The reaction mixtures were suspended with 50 mM NH_4_HCO_3_ (pH 8.4) and digested overnight with trypsin and chymotrypsin at 37 °C. After inactivating the enzymes at 100 °C for 10 min, the pH of the mixture was adjusted below 7 by addition of acetic acid. To remove hydrophilic compounds such as free oligosaccharides, the digests were loaded onto a Sep-Pak C18 column (Waters Co., Milford, MA, USA). After washing with 5% acetic acid, the mixture of peptides and glycopeptides was eluted by stepwise addition of 20%, 40%, and 60% 2-propanol in 5% acetic acid, and then lyophilized. *N*-Glycans were released by GAF treatment in 10 mM NH_4_HCO_3_ (pH 7.8) at 37 °C overnight^47^. The reaction mixtures were adjusted to around pH 5.0 by adding acetic acid and incubated at 37 °C for 30 min. The mixtures were loaded onto 1 ml Dowex 50 W × 2 (H^+^ form, 200–400 mesh) packed in an Econo-column (Bio-Rad, Hercules, CA, USA). The column was washed with 5 ml of water, and the collected effluents were lyophilized. The released *N*-glycans were derivatized with PA as described previously^48^. Mixtures of PA-*N*-glycans were separated by HPLC using a TSKgel DEAE-5PW column as described previously^39,49^. PA-glycans were detected using an FLD with an excitation wavelength of 310 nm and an emission wavelength of 380 nm.

### Linkage-specific derivatization of sialic acids and permethylation

To determine the linkages of sialic acids on non-reducing termini, portions of sialylated PA-*N*-glycans were derivatized with linkage-specific alkylamidation as described previously^39^. To decrease the risk of misconversion, reagents were removed from the reaction mixture with cotton HILIC tips after the first alkylamidation with iPA, and then the second alkylamidation was performed with MA. For permethylation of alkylamidated PA-*N*-glycans, sialylated glycans were alkylamidated and permethylated sequentially as described previously^40^. For permethylation of sulfated PA-*N*-glycans, the reaction mixtures were incubated at 4 °C for 3 h, as described previously^50^. After permethylation, samples were desalted by solid-phase extraction on OASIS PRiME HLB (30 mg, Waters Co., Milford, MA, USA).

### Exoglycosidase digestions

Sialylated PA-*N*-glycans were digested with neuraminidase. Neutral or desialylated PA-*N*-glycans were digested with α1-3,4 fucosidase, and then with β1-4 galactosidase. These enzymatic reactions were performed in 50 mM sodium acetate (pH 5.5) with 5 mM CaCl_2_ at 37 °C for 16–48 h. After each glycosidase digestion, the products were analyzed by LC–MS and MS/MS as described below.

### Online LC–MS, MS/MS, and MS^n^ analysis of glycans

MS analysis of PA-*N*-glycans was performed by ESI–MS on an LTQ XL linear ion trap mass spectrometer coupled to a Dionex U3000 HPLC system and an ESI-probe (H-ESI-II, Thermo Fisher Scientific, Waltham, MA, USA). MS data were recorded and analyzed using the Xcalibur 2.2 software (Thermo Fisher Scientific). For general use, MS and MS/MS data were collected in data-dependent mode, and the top five full MS peaks in each scan event were selected for MS/MS analysis. MS^n^ experiments were performed by selected precursor ion.

PA-*N*-glycans with or without exoglycosidase digestions were separated by reversed-phase LC using an InertSustain AQ-C18 column (2.1 × 150 mm) at a flow rate of 0.2 ml/min at 30 °C. Elution was performed using Eluent A [0.2% (v/v) formic acid] and Eluent B (0.2% formic acid in 20% acetonitrile). The column was equilibrated with Eluent A, and 3 min after sample injection, the proportion of Eluent B was increased linearly from A:B = 100:0 to 80:20 over 80 min, and then to 0:100 over 5 min.

For alkylamidated PA-*N*-glycans, at 3 min after sample injection, the proportion of Eluent B was increased linearly from A:B = 100:0 to 50:50 over 80 min, and then to 0:100 over 5 min.

Permethylated PA-*N*-glycans with or without alkylamidation were separated by reversed-phase LC using an InertSustain AQ-C18 column (2.1 × 250 mm) at a flow rate of 0.2 ml/min at 50 °C. Elution was performed using Eluent A′ (water) and Eluent B′ (acetonitrile). For permethylated neutral PA-*N*-glycans, the column was equilibrated with 10% Eluent B′, and the proportion of Eluent B′ was increased linearly from A′:B′ = 90:10 to 52:48 over 42 min, and maintained for 58 min. The proportion of Eluent B′ was increased linearly to 10:90 over 38 min, and maintained for 9 min. For permethylated sialylated and/or sulfated PA-*N*-glycans with or without alkylamidation, the column was equilibrated with 10% Eluent B′, and the proportion of Eluent B′ was increased linearly from A:B = 90:10 to 50:50 over 44 min, and maintained for 56 min. Next, the proportion of Eluent B′ was increased linearly to 10:90 over 38 min, and maintained for 9 min.

Eluents were separated evenly (1:1) using an ASI flow splitter 600-PO-10–06 (Analytical Scientific Instruments, Richmond, CA, USA) and directed to the MS and FLD, respectively. PA-glycans were detected by MS, as described below, and simultaneously detected using an FLD with an excitation wavelength of 315 nm and an emission wavelength of 400 nm. For permethylated PA-*N*-glycans, the eluate for MS was mixed with 4 mM NaOH in 50% acetonitrile from a post-column syringe pump at a flow rate of 1.5 μl/min, resulting in the formation of sodium adducts.

MS conditions were as follows: (1) for PA-*N*-glycans with or without SALSA: spray voltage = 4 kV, auxiliary gas flow rate = 2 arb, sheath gas flow rate = 30 arb, heated capillary temperature = 250 °C, heated capillary voltage = 40 V, and tube lens voltage = 75 V; (2) for PA-*N*-glycans with permethylation: spray voltage = 4 kV, auxiliary gas flow rate = 2 arb, sheath gas flow rate = 20 arb, heated capillary temperature = 250 °C, heated capillary voltage = 40 V, and tube lens voltage = 100 V.

Glycan structures were deduced based on the results of MS, MS/MS, and MS^n^ as well as known biosynthetic pathways of vertebrate glycans. The standard Symbol Nomenclature for Glycan system was used for monosaccharide symbols^51^, except for sulfate and phosphate groups. The relative amount of each PA-*N*-glycan was quantified based on the integration of fluorescence signals after LC separation. When fluorescence intensity peaks included more than two kinds of PA-glycans with different mass values, their proportions were estimated using the ratios of integrated ion intensities for each *m/z* value detected at the corresponding times.

## Supplementary Information

Supplementary Information 1.

Supplementary Information 2.

Supplementary Information 3.

Supplementary Information 4.
