# Supplementary material for: Toward robust N-glycomics of various tissue samples that may contain glycans with unknown or unexpected structures
Source: Sci Rep. 2021 Mar 18;11:6334. doi: 10.1038/s41598-021-84668-x (PMC7973440; doi:10.1038/s41598-021-84668-x)
Supplement: Supplementary file 1 — Supplementary Information 1. [file 41598_2021_84668_MOESM1_ESM.pdf]

Supporting information

Toward robust *N*-glycomics of various tissue samples that may contain glycans with unknown or unexpected structures

Noriko Suzuki[1,*], Tatsuya Abe[1], Ken Hanzawa[1], and Shunji Natsuka[1,2]

[1]Graduate School of Science and Technology and [2]Faculty of Science, Niigata University, 5050 Ikarashi-nino-cho, Nishi-ku, Niigata 950-2181, Japan

*To whom correspondence should be addressed: E-mail: nrsuzuki@gs.niigata-u.ac.jp

Supplementary Results

Supplementary Figures S1, S2, S3-1–S3-15, S4, and S5-1–S5-5

Supplementary Table S1

Supplementary Table S2

Supplementary Results

Overview of *N*-glycans detected by LC-MS and MS/MS analysis

PA-derivatized *N*-glycans from chicken colon were separated into 11 fractions by HPLC using an anion-exchange DEAE column, based on the negative charge of the glycans (Supplementary Fig. S1A). Each fraction was analyzed by LC-MS and MS/MS using a C18 reversed-phase LC column, and PA-glycans were simultaneously monitored with a fluorescence detector (FLD). As reference standards, PA-*N*-glycans from human γ-globulin, bovine fetuin, human α1-acid glycoprotein (α1-AGP), human transferrin, and their enzymatic derivatives (Supplementary Fig. S2) were also analyzed under the same LC-MS and MS/MS conditions. Ten of the eleven fractions (fr. 1, 3–11) from the DEAE column contained PA-*N*-glycans (Supplementary Fig. S1B), and the exception (fr. 2) may have contained non-glycan fluorescence contaminants, as often observed in PA-derivatized glycan mixtures (data not shown). Based on the results of full MS and MS/MS analysis, we deduced the monosaccharide compositions and approximate branch sequences of each glycan (Supplementary Table S1), although detailed structural features such as glycosidic linkages and branching patterns remained ambiguous during this initial step. For convenience, deoxyhexoses were indicated as Fuc, unless otherwise noted.

The deduced monosaccharide compositions of PA-*N*-glycans indicated the presence of negatively charged functional groups, such as phosphate and sulfate moieties, as well as Sia residues on PA-*N*-glycans, which were separated from neutral glycans on the DEAE column. Because addition of a phosphate or sulfate group to a

glycan results in a similar increment in mass (~80 mass units per group), these modifications could not be distinguished from each other using our MS instrument, which had a relatively low resolution. Thus, we tentatively assigned glycan structures containing these groups based on the known biosynthetic pathways of *N*-glycans, i.e., phosphate groups on high mannose-type glycans (fr. 3, 6–8 in Supplementary Table S1) and sulfate groups on complex/hybrid-type glycans (fr. 3, 6, 7, 10, 11 in Supplementary Table S1). The deduced monosaccharide compositions also revealed that PA-*N*-glycans from chicken colon could be separated into neutral (fr. 1), monosialyl (fr. 3), disialyl (fr. 4–6), trisialyl (fr. 7, 8), tetrasialyl (fr. 9, 10), and pentasialyl (fr. 11) structures.

In the neutral fraction (fr. 1), most high mannose-type oligosaccharides eluted from the C18 column earlier than complex/hybrid-type oligosaccharides, as reported previously[1,2]. The structures of these high mannose-type glycans were deduced based on their elution positions relative to the previous reports. The most abundant *N*-glycan in the chicken colon was $Man_5GlcNAc_2$-PA, and we calculated the relative amount of each *N*-glycan in the tissue (Supplementary Table S1). Among the complex/hybrid-type oligosaccharides in the neutral fraction, the proportion of digalactosyl biantennary structures whose compositions were assigned as $Hex_2HexNAc_{2–3}Fuc_{0–1}C$-PA (C is the trimannosyl core structure; $Man_3GlcNAc_2$), was much higher than others. LC-MS data indicated that some PA-*N*-glycans with the same composition were eluted at different times on reversed-phase LC, suggesting the presence of some isomers (Supplementary Fig. S3-1A, Table S1). For instance, the extracted ion chromatogram (EIC) at *m/z* 860.33 [$Hex_2HexNAc_2C$-PA($2H^+$)] indicated three major peaks at 38.98, 43.80, and

45.87 min (Supplementary Fig. S3-1A). MS/MS data of the peak around 43.80 min were obviously different from those of the other two peaks (Supplementary Fig. S3-1B), and exhibited large peaks of B ion fragments at *m/z* 366, a hallmark of $Hex_1HexNAc_1$, and Y ion fragments at *m/z* 1355, generated by loss of $Hex_1HexNAc_1$, suggesting the presence of the LacNAc (Gal-GlcNAc) sequence on the branches. Among the three isomers, the PA-*N*-glycan eluted around 43.80 min was identical to Standard E (Supplementary Fig. S2) in terms of elution time, composition, and MS/MS spectrum. By contrast, the two isomers that eluted at 38.98 and 45.87 min had MS/MS spectra similar to each other (Supplementary Fig. S3-1B). The major peak of the Y ion fragments at *m/z* 1517 [$Hex_2HexNAc_1$C-PA, (H^+)] was generated by loss of one HexNAc, suggesting the presence of HexNAc at non-reducing termini. Relatively low signals at *m/z* 366 and 1355 implied the absence of the LacNAc sequence on branches. Taking together with the presence of small peaks at *m/z* 1151 [$Hex_4HexNAc_2$-PA, (H^+)] and 1314 [$Hex_5HexNAc_2$-PA, (H^+)], these observations imply that these structures are most likely to be non-galactosylated hybrid-type glycans. Thus, we proposed two candidate structures with the composition $GlcNAc_2Man_5GlcNAc_2$-PA (= $Man_2GlcNAc_2$C-PA). Of the two, the one with a bisecting GlcNAc probably eluted around 45.87 min, as in the case of biantennary glycans with bisecting GlcNAc (e.g., Standard K in Supplementary Fig. S2), which tends to elute later on C18 columns than triantennary structures with the same composition (e.g., Standard W)[2,3]. Two minor peaks on EIC at *m/z* 860.33 [$Hex_2HexNAc_2$C-PA($2H^+$)] that eluted around 26.26 and 30.88 min (Supplementary Fig. S3-1A) were most likely artificial by-products

generated by C-2 epimerization of reducing termini, i.e., conversion from GlcNAc to ManNAc, under conditions (pH 7–8) that promote release of *N*-glycans from glycopeptides. Small amounts of epimers with ManNAc are generated from released *N*-glycans, and their PA-derivatives are eluted earlier on reversed-phase HPLC than the corresponding non-epimerized PA-*N*-glycans[2]. Because the glycan structures of the non-reducing sides are the same, the fingerprints of MS/MS spectra of C-2 epimers are almost the same as those of the original glycans. EIC at *m/z* 933.36 [$Hex_2HexNAc_2Fuc_1C$-PA($2H^+$)] exhibited four major peaks at 42.04, 48.59, 54.26, and 59.40 min (Supplementary Fig. S3-1A). Among them, based on the elution position, full MS, and MS/MS data (Supplementary Fig. S3-1B), the PA-*N*-glycan that eluted around 54.26 min is most likely structurally identical to Standard F (Supplementary Fig. S2), which possesses core Fuc. The PA-*N*-glycan that eluted around 42.04 min is most likely the epimer of the one that eluted around 54.26 min, as the MS/MS fingerprints were almost the same (data not shown). Two other peaks of PA-*N*-glycans are most likely non-galactosylated hybrid-type glycans with core Fuc, similar to the non-fucosylated counterparts described above, as evidenced by the analogous fragmentation patterns in the MS/MS analysis of these isomers, e.g., preferential loss of one HexNAc. These hybrid-type glycans, with or without core Fuc, showed resistance to β1-4 galactosidase digestion and maintained consistent elution positions on reversed-phase LC (data not shown), confirming the absence of β1,4-galactose residues on the non-reducing termini. Based on the results of full MS, MS/MS, and their elution positions (Supplementary Fig. S3-1A), PA-*N*-glycans of the major peaks on EICs at *m/z* 961.87

[$Hex_2HexNAc_3C$-PA($2H^+$)], which eluted around 60.55 min, and at *m/z* 1034.90 [$Hex_2HexNAc_3Fuc_1C$-PA($2H^+$)], which eluted around 74.12 min, were identical to Standard G and Standard H, respectively. Comparison of the elution positions of these biantennary PA-*N*-glycans on reversed-phase LC suggested that addition of core Fuc, bisecting GlcNAc, or core Fuc/bisecting GlcNAc made a strong positive contribution to the retention in the range of 10–11 min, 16–17 min, or 30–31 min, respectively. These empirical additivity rules are consistent with previous reports describing separation of PA-labeled glycans with C18 columns[2,3].

MS and MS/MS analysis also indicated that some PA-*N*-glycans contain Fuc residues on branch positions characterized by B ion fragments at *m/z* 512 ($Hex_1HexNAc_1Fuc_1$) and *m/z* 803 ($Hex_1HexNAc_1Fuc_1NeuAc_1$), LacdiNAc (GalNAc-GlcNAc) sequences characterized by B ion fragments at *m/z* 407 ($HexNAc_2$), and LacNAc repeats characterized by B ion fragments at *m/z* 731 ($Hex_2HexNAc_2$) (Supplementary Table S1). The estimated monosaccharide compositions suggested the presence of highly branched structures, such as tri-, tetra-, or pentaantennary *N*-glycans, and these assumptions were confirmed as described in the following sections.

Determination of anomeric configurations and branching/core structures by exoglycosidase digestions

Exoglycosidase digestions---To clarify the sequences of branches and branching patterns of *N*-glycans from chicken colon, each fraction of PA-*N*-glycans was subjected to sequential exoglycosidase digestions using neuraminidase, α1-3,4 fucosidase, and

β1-4 galactosidase. After each enzymatic treatment, reaction mixtures were analyzed by LC-MS and MS/MS, similarly to the non-digested samples. Fig. 3 shows examples of elution profiles after exoglycosidase digestion for PA-*N*-glycans in fr. 3, which contained monosialylated or monosulfated glycans. The results of LC-MS and MS/MS revealed that NeuAc was removed completely after neuraminidase digestion, and the elution profile of PA-*N*-glycans in fr. 3 was dramatically changed by this treatment. By contrast, the elution profiles after α1-3,4 fucosidase digestion were mostly unchanged, although some peaks shifted by loss of one or two Fuc resides, suggesting the presence of α3/4-Fuc on *N*-glycans. After β1-4 galactosidase digestion, the elution profile changed dramatically again, suggesting that the majority of complex/hybrid-type *N*-glycans possess type II LacNAc (Galβ1-4GlcNAc). However, some minor PA-*N*-glycans retained one LacNAc sequence even after treatment with a sufficient amount of β1-4 galactosidase, implying the presence of type I LacNAc (Galβ1-3GlcNAc) as a minor component.

Next, to find elution rules that would facilitate deduction of glycan structures, we compared the elution positions of some PA-*N*-glycans using LC-MS and MS/MS data as follows:

Asialo-biantennary structures with or without core Fuc and/or bisecting GlcNAc---Elution positions of asialo-biantennary structures with the composition $Hex_2HexNAc_{2–3}Fuc_{0–1}$C-PA were detected in EICs of neuraminidase/α1-3,4 fucosidase–treated fr. 3 (Fig. 4A, Supplementary Fig. S3-2A), similar to those of PA-*N*-glycans in

fr. 1 (Supplementary Fig. S3-1A). Comparison of the elution positions of these biantennary PA-*N*-glycans on reversed-phase LC suggested that addition of core Fuc, bisecting GlcNAc, and core Fuc/bisecting GlcNAc made a strong positive contribution to the retention in the ranges of 10–11 min, 16–17 min, and 30–31 min, respectively. Similar results were obtained from the EICs of agalactosyl biantennary structures with the composition $HexNAc_{2–3}Fuc_{0–1}C$-PA from neuraminidase/α1-3,4 fucosidase/β1-4 galactosidase–treated fr. 3 (Fig. 4B, Supplementary Fig. S3-6A).

Monosialylated biantennary structures---Elution positions of monosialylated biantennary structures with the composition $Hex_2HexNAc_{2–3}Fuc_{0–1}NeuAc_1C$-PA, were also detected in EICs of fr. 3 without exoglycosidase digestions (Supplementary Fig. S3-2B). Linkages and positions of NeuAc on biantennary branches were deduced by comparing the elution positions, full MS, and MS/MS spectra of these PA-*N*-glycans with those of monosialylated standards (Standard a–h in Supplementary Fig. S2). Some of the PA-*N*-glycans from chicken colon did not match any standards we used in terms of these three criteria. In those cases, we deduced their structures based on the three criteria, as well as the results of MS analysis with sialic acid linkage-specific alkylamidation (SALSA, Supplementary Table S2), as described in the next section. MS/MS spectra of asymmetric biantennary glycan structures, e.g., those with one of two branches occupied with Sia, differed from those of the symmetric counterparts (Supplementary Fig. S3-2C and D). Comparison of the relative intensities of the B ion fragments at *m/z* 366 ($Hex_1HexNAc_1$) versus *m/z* 657 ($Hex_1HexNAc_1NeuAc_1$) or those

of the Y ion fragments of [precursor - $Hex_1HexNAc_1$](H^+) versus [precursor - $Hex_1HexNAc_1NeuAc_1$](H^+) suggested that a ±Sia-LacNAc branch linked to α3-Man of the trimannosyl core was more preferentially to be fragmented by collision-induced dissociation (CID) than the branch linked to α6-Man, regardless of the presence or absence of NeuAc. This empirical rule in our system was consistent at least for divalent protonated precursor ions, but may not be applicable to trivalent precursor ions (Supplementary Fig. S3-2D). Moreover, comparison of the elution positions of biantennary PA-*N*-glycans with or without monosialylation on reversed-phase LC (Supplementary Fig. S3-2A, B) revealed that addition of one NeuAc at the non-reducing terminus resulted in different contributions to the retention depending on linkage type (α2,3 or α2,6-Sia) and the positions on branches. The addition of α2,3-NeuAc contributed positively in the range of 6–8 min (without bisecting GlcNAc, with or without core Fuc), 4–10 min (with bisecting GlcNAc, without core Fuc), or 3–6 min (with bisecting GlcNAc, with core Fuc), whereas the addition of α2,6-NeuAc attached to LacNAc on α3-Man made a smaller contribution to retention: less positive (0–2 min, without bisecting GlcNAc, with or without core Fuc) or even negative (1–4 min, with bisecting GlcNAc, with or without core Fuc).

Fucosylated biantennary structures---Next, using EICs of neuraminidase–treated fr. 3, we compared the elution positions of fucosylated biantennary structures with bisecting GlcNAc assigned as $Hex_2HexNAc_3Fuc_{1-3}$C-PA (Supplementary Fig. S3-3A). EICs at *m/z* 1034.90 [$Hex_2HexNAc_3Fuc_1$C-PA($2H^+$)] exhibited one major peak and two minor

peaks. The elution time, full MS, and MS/MS spectrum of the PA-*N*-glycan in the major peak that eluted around 74.07 min were substantively similar to those of Standard H (Supplementary Fig. S2), which possesses core Fuc and bisecting GlcNAc. One of the minor peaks eluted around 53.63 min is probably a C-2 epimer of the PA-*N*-glycan that eluted around 74.07 min, as their MS/MS spectra were analogous (data not shown). By contrast, the PA-*N*-glycan in the other minor peak that eluted around 47.14 min more readily lost Fuc residues, as revealed by MS/MS analysis (Supplementary Fig. S3-3B), than PA-*N*-glycans with core Fuc (Standard H, PA-*N*-glycans eluted around 74.07 min), suggesting that Fuc was present on a branch. The presence of the hallmark B ion fragments at *m/z* 512 ($Hex_1HexNAc_1Fuc_1$) supported this assumption. In comparison with the elution position of the non-fucosylated biantennary PA-*N*-glycan with bisecting GlcNAc [*m/z* 961.87, $Hex_2HexNAc_3C$-PA($2H^+$), identical to Standard G], which eluted around 60.41 min (Supplementary Fig. S3-3A), the addition of one core Fuc made a strong positive contribution to retention (13–14 min). By contrast, the addition of one Fuc residue on a branch made a strong negative contribution to retention (10–15 min).

EICs at *m/z* 1107.93 [$Hex_2HexNAc_3Fuc_2C$-PA($2H^+$)] exhibited one major peak and three minor peaks, except some unrelated artificial minor peaks. The minor peak that eluted around 44.70 min is most likely a C-2 epimer of the PA-*N*-glycan that eluted around 59.62 min, as the MS/MS spectra of the two peaks were almost identical (data not shown). The MS/MS spectra of the PA-*N*-glycans at *m/z* 1107.93 that eluted around 37.15, 59.62, or 61.68 min were similar but slightly different each other (Supplementary

Fig. S3-3B). The presence of the B ion fragments at *m/z* 512 ($Hex_1HexNAc_1Fuc_1$) and several Y ion fragments suggested the presence of Fuc on one or two branches. However, these MS/MS spectra did not provide strong evidence regarding the position of Fuc, as Fuc on branches can easily transfer to different positions by rearrangement[4]. Instead, we compared the elution positions of these three isomers. As described above, the addition of one core Fuc made a strong positive contribution to retention (13–14 min), whereas the addition of one Fuc residue on a branch made a strong negative contribution to retention (10–15 min). Therefore, we assumed that the PA-*N*-glycan that eluted around 37.17 min possesses two Fuc residues on branches, whereas those that eluted around 59.62 and 61.68 min possess one core Fuc and one Fuc on a branch. The later two PA-*N*-glycans may differ in terms of the position of Fuc on one of the two LacNAc branches, i.e., fucolylated LacNAc may be linked to either the α3-Man or α6-Man arm. By analogy, the PA-*N*-glycans at *m/z* 1180.96 [$Hex_2HexNAc_3Fuc_3C$-PA($2H^+$)] that eluted around 48.01 min (Supplementary Fig. S3-3A) most likely has one core Fuc and two Fuc residues on branches. All the Fuc residues on branches of PA-*N*-glycans from chicken colon were removed by α1-3,4 fucosidase digestion (Fig. 3), whereas core Fuc was retained on the glycans. The positions of Fuc residues on the LacNAc branch and their linkages were confirmed by permethylation, as described below.

Sulfated biantennary structures---Several minor PA-*N*-glycans from chicken colon possessed one or two sulfate groups. For example, sulfated biantennary PA-*N*-glycans,

assigned as $Hex_2HexNAc_2Fuc_1NeuAc_{0–2}(SO_3)_1C$-PA, were detected in fr. 3, 6, 7, and 10. EICs of these sulfated PA-*N*-glycans (Supplementary Fig. S3-4A) indicated the presence of several isomers that differed in terms of the linkages and positions of NeuAc. These isomers eluted at the same position after neuraminidase digestion. The MS/MS spectra of these isomers supported the presence of a sulfate group (Supplementary Fig. S3-4B). However, we could not determine the position of the sulfate group from these MS/MS spectra alone, because sulfate groups can easily transfer to different positions by rearrangement. The linkages and positions of NeuAc, as well as the position of the sulfate group, were determined by SALSA and permethylation, as described below. The addition of a sulfate group to PA-*N*-glycans influenced the elution positions on reversed-phase LC, but the contribution was complex, and differed depending on the presence or absence of NeuAc. In the case of non-sialylated biantennary PA-*N*-glycans with core Fuc without bisecting GlcNAc, the sulfate group positively contributed to retention in the range of 2–3 min. By contrast, the group contributed negatively for the glycans with one α2,6-NeuAc or α2,3-NeuAc (0–4 min), two α2,6-NeuAc (2–3 min), or two α2,3-NeuAc (6–7 min). These observations indicate that the positive contribution of addition of NeuAc to biantennary PA-*N*-glycans without bisecting GlcNAc was abolished by the addition of a sulfate group.

Triantennary structures---EICs at *m/z* 1042.90, 1115.93, 1144.44, and 1217.47 of PA-*N*-glycans in neuraminidase/α1-3,4 fucosidase–treated fr. 3, assigned as

$Hex_3HexNAc_{3–4}Fuc_{0–1}C$-PA, exhibited several isomer peaks (Fig. 4C, Supplementary Fig. S3-5A). The PA-*N*-glycan corresponding to the peak at 53.37 min in EIC at *m/z* 1042.90 [$Hex_3HexNAc_3C$-PA($2H^+$)] was identical to Standard S (Supplementary Fig. S2) in terms of elution time, composition, and MS/MS spectrum. The structure of Standard S has one of the two major types of triantennary *N*-glycans (2,4,2′-tri in Fig. 1B) found to date in vertebrates. According to the empirical rules governing the elution positions of PA-*N*-glycans on reversed-phase LC with a C18 column, PA-*N*-glycans with a 2,4,2′-triantennary structure generally elute later than those with a cognate 2,2′-biantennary structure, e.g., Structure S versus Structure E[2,3]. By contrast, PA-*N*-glycans with a 2,2′,6′-triantennary structure (Fig. 1B) generally elute earlier than those with a cognate 2,2′-biantennary structure. These differences are attributable to the contribution of branching GlcNAc linked to either the C-4 position of α3-Man or the C-6 position of α6-Man. If this empirical rule is also applicable to our system, then the PA-*N*-glycan at *m/z* 1042.90 [$Hex_3HexNAc_3C$-PA($2H^+$)] that eluted around 35.24 min (Supplementary Fig. S3-5A) could be a 2,2′,6′-triantennary *N*-glycan with LacNAc branches. The MS/MS spectra of these peaks supported this assumption (Supplementary Fig. S3-5B). The PA-*N*-glycan with the same composition that eluted around 64.09 min was assigned as a hybrid-type *N*-glycan with a bisecting GlcNAc and two LacNAc branches, because it eluted later than Standard S and preferentially lost one HexNAc in MS/MS analysis (Supplementary Fig. S3-5B). As in the case of biantennary structures, bisecting GlcNAc made a strong positive contribution to retention.

Similar relationships of triantennary structures were also observed in

PA-*N*-glycans with core Fuc and/or bisecting GlcNAc (Fig. 4C, Supplementary Fig. S3-5A). Notably, PA-*N*-glycans with the composition $Hex_3HexNAc_3Fuc_1$C-PA that eluted around 62.92 min and those with the composition $Hex_3HexNAc_4Fuc_1$C-PA that eluted around 79.85 min had a LacNAc repeat sequence (Gal-GlcNAc-Gal-GlcNAc, $LacNAc_2$). The MS/MS spectra of these glycans contained strong signals of the hallmark B ion fragments at *m/z* 731 ($Hex_2HexNAc_2$) and the Y ion fragments of [precursor - $Hex_2HexNAc_2$](H^+) (Supplementary Fig. S3-5B). The presence of LacNAc repeats was also confirmed by permethylation, as described below. Among the PA-*N*-glycans with the compositions $Hex_3HexNAc_3Fuc_1$C-PA, triantennary structures lost three β4-Gal residues at non-reducing termini, whereas the biantennary structures with a $LacNAc_2$ or the hybrid-type *N*-glycan lost two β4-Gal residues upon β1-4 galactosidase digestion (EICs and MS/MS data at *m/z* 872.85 and *m/z* 953.87 in Supplementary Fig. S3-6A, B). This observation supports the deduced structures.

LacdiNAc and branching patterns---To compare the elution positions among PA-*N*-glycans with different branching patterns, we used EICs of chicken colon PA-*N*-glycans with several compositions ($HexNAc_{2–6}Fuc_{0–1}$C-PA) prepared by neuraminidase/α1-3,4 fucosidase/β1-4 galactosidase digestions. EICs of agalactosyl biantennary PA-*N*-glycans at *m/z* 698.28 [$HexNAc_2$C-PA($2H^+$)] and 771.31 [$HexNAc_2Fuc_1$C-PA($2H^+$)] in fr. 3 exhibited a single peak except its C-2 epimer. By contrast, EICs at *m/z* 799.82 [$HexNAc_3$C-PA($2H^+$)] and 872.85 [$HexNAc_3Fuc_1$C-PA($2H^+$)] exhibited more peaks than we expected (Fig. 4D,

Supplementary Fig. S3-6A). Among them, the MS/MS spectra in Supplementary Fig. S3-6B suggested the presence of HexdiNAc, most likely LacdiNAc (GalNAc-GlcNAc), on one of the two arms of the PA-*N*-glycans that eluted around 42.51 min ($HexNAc_3C$-PA), 53.55 min ($HexNAc_3Fuc_1C$-PA), and 56.38 min ($HexNAc_3Fuc_1C$-PA), with the hallmark B ion fragments at *m/z* 407 ($HexNAc_2$). These PA-*N*-glycans also tended to lose two HexNAc residues, resulting in the large Y ion fragments of [precursor - $HexNAc_2$](H^+). The data imply that addition of the second HexNAc (most likely GalNAc) to the first HexNAc (most likely GlcNAc) of HexdiNAc, resulted in a positive contribution to retention in the range of 3–6 min, and that this contribution differed slightly depending on the arm where the HexNAc was added. This moderate contribution of GalNAc is similar to the effect of addition of β4-GalNAc to GlcNAc on LacdiNAc branches as previously reported[3,5]. MS/MS spectra of other peaks on EICs at *m/z* 799.82 or 872.85, which do not possess LacdiNAc, were similar to each other, and exhibited large Y ion fragments of [precursor - $HexNAc_1$](H^+). Thus, we deduced structures of these PA-*N*-glycans based on their elution positions on reversed-phase LC. First, the PA-*N*-glycans that eluted around 47.27 min (at *m/z* 799.82), 54.60 min (at *m/z* 799.82), and 69.40 min (at *m/z* 872.85) were identical to Standards W, K, and L, respectively, in terms of elution time, composition, and MS/MS spectrum. The small peaks that eluted around 36.75 min (at *m/z* 799.82) and 49.33 min (at *m/z* 872.85) were most likely the C-2 epimers of biantennary PA-*N*-glycans with bisecting GlcNAc, as their MS/MS spectra were almost identical (Supplementary Fig. S3-6B). The PA-*N*-glycan that eluted around 31.46 min (at *m/z* 799.82, $HexNAc_3C$-PA)

could be a 2,2′,6′-triantennary structure (Fig. 1B), because it was eluted earlier than agalactosyl biantennary glycan (at *m/z* 698.28, $HexNAc_2C$-PA, eluted around 39.43 min), as described above. Similarly, the PA-*N*-glycans (at *m/z* 872.85, $HexNAc_3Fuc_1C$-PA) that eluted around 43.16 and 59.18 min could be 2,2′,6′-triantennary and 2,4,2′-triantennary structures, respectively, based on their elution positions relative to that of agalactosyl biantennary glycan (at *m/z* 771.31, $HexNAc_2Fuc_1C$-PA, eluted around 50.46 min). EICs of neuraminidase/α1-3,4 fucosidase/β1-4 galactosidase–digested PA-*N*-glycan from chicken colon fr. 3 at *m/z* 901.36 [$HexNAc_4C$-PA($2H^+$)] exhibited three major peaks (Supplementary Fig. S3-6A). The PA-*N*-glycan that eluted around 39.72 min was identical to Standard V in terms of elution time, composition, and MS/MS spectrum. PA-*N*-glycans that eluted around 42.94 and 71.71 min ($HexNAc_4C$-PA) could be 2,2′,6′-triantennary and 2,4,2′-triantennary structures with bisecting GlcNAc, respectively, based on their elution positions relative to that of agalactosyl biantennary glycan with bisecting GlcNAc (at *m/z* 799.82, $HexNAc_3C$-PA, eluted around 54.60 min). Similarly, the PA-*N*-glycans (at *m/z* 974.39, $HexNAc_4Fuc_1C$-PA) that eluted around 50.60, 54.75, and 82.76 min could be 2,4,2′,6′-tetraantennary structures, 2,2′,6′-triantennary structures with bisecting GlcNAc, and 2,4,2′-triantennary structures with bisecting GlcNAc, respectively, with core Fuc. The MS/MS spectrum of a small peak that eluted around 46.38 min (at *m/z* 974.39) indicated the hallmark B ion fragments at *m/z* 407 ($HexNAc_2$) (data not shown, but refer to the MS/MS data in Supplementary Fig. S3-7B for the same structure found in fr. 1), suggesting that this PA-*N*-glycan contains a LacdiNAc

sequence. This LacdiNAc is probably on a 2,2′,6′-triantennary glycan, as the elution position of this PA-*N*-glycan was close to that of agalactosyl 2,2′,6′-triantennary glycan ($HexNAc_3Fuc_1C$-PA, eluted around 43.16 min). Comparison of the reversed-phase LC elution positions of 2,2′,6′- and 2,4,2′-triantennary PA-*N*-glycans to those of cognate biantennary PA-*N*-glycans with or without core Fuc but lacking bisecting GlcNAc indicated that addition of β6′-GlcNAc or β4-GlcNAc made a negative (7–8 min) or positive (7–9 min) contribution to retention, respectively. Given that β6′-GlcNAc and β4-GlcNAc exerted opposing effects, 2,4,2′,6′-tetraantennary PA-*N*-glycans, that contain both β6′-GlcNAc and β4-GlcNAc eluted at positions similar to those of 2,2′-biantennary PA-*N*-glycans. These empirical additivity rules were consistent among PA-labeled *N*-glycans separated with C18 columns, as reported previously[2,3]. In PA-*N*-glycans with bisecting GlcNAc, the negative and positive contributions of β6′-GlcNAc and β4-GlcNAc were similar but stronger (11–15 min and 13–18 min, respectively) than those of PA-*N*-glycans without bisecting GlcNAc.

We further compared EICs at *m/z* 974.39 [$HexNAc_4Fuc_1C$-PA($2H^+$)] of neuraminidase/α1-3,4 fucosidase/β1-4 galactosidase–digested PA-*N*-glycan from chicken colon fr. 1 and 3–11. Among them, EICs of fr. 1, 3–5, 7, 8, and 10 exhibited at least one PA-*N*-glycan peak (Fig. 4D, Supplementary Fig. S3-7A). The MS/MS spectra of each peak at *m/z* 974.39 in the glycosidase–digested fr. 1 and fr. 4 are shown in Supplementary Fig. S3-7B. The PA-*N*-glycans that eluted around 50.81, 54.75, and 82.72 min (fr. 1) should be the same as those detected in the glycosidase–digested fr. 3 described above. The PA-*N*-glycan that eluted around 41.78 min (fr. 1) was most likely

the C-2 epimer of the one that eluted around 54.75 min, as their MS/MS spectra were substantively similar (Supplementary Fig. S3-7B). We assumed that the remaining four peaks in fr. 1 contained one or two LacdiNAc branches, because they exhibited the hallmark B ion fragments at *m/z* 407 ($HexNAc_2$). Considering the rule that the addition of one HexNAc (probably β4-GalNAc) to GlcNAc on an arm contributes positively to retention in the range of 3–6 min, as described above, the core structures of PA-*N*-glycans with core Fuc that eluted around 46.69, 60.01, 64.56, or 76.56 min were most likely 2,2′,6′-tri, 2,2′-bi, 2,4,2′-tri, or 2,2′-bi with bisecting GlcNAc, respectively. The deduced structures of the PA-*N*-glycan that eluted around 60.01 min, which contains two LacdiNAc branches, was supported by the MS/MS data (Supplementary Fig. S3-7B), which contained relatively high signals of the B ion fragments at *m/z* 407 ($HexNAc_2$) and the Y ion fragments at *m/z* 1542 [precursor - $HexNAc_2$](H^+).

The deduced monosaccharide compositions of PA-*N*-glycans from chicken colon (Supplementary Table S1) suggested the presence of a pentaantennary structure. This is not surprising, as chicken egg white glycoproteins such as ovomucoid contain pentaantennary *N*-glycans[6]. To confirm the presence of pentaantennary *N*-glycans, we examined the EICs of PA-*N*-glycans with compositions of $HexNAc_{5–6}Fuc_{0–1}$C-PA($2H^+$) using data from the LC-MS and MS/MS analysis of each neuraminidase/α1-3,4 fucosidase/β1-4 galactosidase–digested each fraction. As shown in Supplementary Fig. S3-8A, some PA-*N*-glycans with such compositions were detected in several fractions, although their relative levels were low. For example, the EIC at *m/z* 1002.90 [$HexNAc_5$C-PA($2H^+$)] of glycosidase–digested fr. 4 contained three major peaks. The

MS/MS spectra of these three peaks (Supplementary Fig. S3-8B) were similar but not identical. The lack of a strong signal at *m/z* 407 ($HexNAc_2$) suggested the absence of a LacdiNAc sequence. Unfortunately, we could not compare these MS/MS spectra with those of appropriate standards; therefore, we deduced the structures by comparing their relative elution positions with those of PA-*N*-glycans whose compositions were assigned as $HexNAc_4C$-PA($2H^+$) (EIC at *m/z* 901.36 in Supplementary Fig. S3-6A). For example, the PA-*N*-glycan with composition $HexNAc_4C$-PA that eluted around 71.71 min contained a 2,4,2′-triantennary structure with bisecting GlcNAc, as described above. As also mentioned above, addition of β6′-GlcNAc to α6-Man on PA-*N*-glycans with bisecting GlcNAc contributed negatively to retention (11–15 min). Thus, the PA-*N*-glycan with the composition $HexNAc_5C$-PA that eluted around 58.76 min probably had a 2,4,2′,6′-tetraantennary structure with bisecting GlcNAc. On the other hand, the PA-*N*-glycans with compositions assigned as $HexNAc_4C$-PA that eluted around 39.72 or 42.94 min could have been a 2,4,2′,6′-tetraantennary structure or 2,2′,6′-triantennary structure with bisecting GlcNAc, respectively, as described above (EIC at *m/z* 901.36 in Supplementary Fig. S3-6A). We assumed that addition of β4′-GlcNAc to α6-Man on PA-*N*-glycans with or without bisecting GlcNAc would contribute negatively (0–3 min) or positively (1–2 min) to retention, respectively. If so, PA-*N*-glycans with composition $HexNAc_5C$-PA that eluted around 41.61 min and 42.73 min could have been a 2,4,2′,4′,6′-pentaantennary structure and 2,2′,4′,6′-tetraantennary structure with bisecting GlcNAc, respectively. By analogy, PA-*N*-glycans with the composition $HexNAc_5Fuc_1C$-PA that eluted around 52.22, 53.26,

or 72.23 min (Fig. 4D, Supplementary Fig. S3-8A) could have been a 2,4,2′,4′,6′-pentaantennary structure with core Fuc, a 2,2′,4′,6′-tetraantennary structure with core Fuc and bisecting GlcNAc, or a 2,4,2′,6′-tetraantennary structure with core Fuc and bisecting GlcNAc, respectively. Moreover, if the assumption that the addition of β4′-GlcNAc to PA-*N*-glycans with bisecting GlcNAc contributes negatively (0–3 min) is correct, then the PA-*N*-glycan with composition $HexNAc_6$C-PA that eluted around 57.17 min could have been 2,4,2′,4′,6′-pentaantennary structures with bisecting GlcNAc without core Fuc. Similarly, the PA-*N*-glycan with composition $HexNAc_6Fuc_1$C-PA that eluted around 69.64 min (Fig. 4D, Supplementary Fig. S3-8A) could have been a 2,4,2′,4′,6′-pentaantennary structure with bisecting GlcNAc and core Fuc. It should be noted that both 2,4,2′,4′,6′-pentaantennary structures and 2,2′,4′,6′-tetraantennary structures are rarely found in mammals.

LacNAc repeats and multiantennary structures with LacNAc---Compositions of the complex-type PA-*N*-glycans from chicken colon were mainly $Hex_nHexNAc_nFuc_{0–1}NeuAc_{0–5}$C-PA or $Hex_nHexNAc_{(n+1)}Fuc_{0–1}NeuAc_{0–4}$C-PA ($n$ = 2–5), with some exceptions, suggesting the presence of multiantennary structures, extended LacNAc repeat sequences, or both. To confirm the presence of these structures, we examined the EICs of PA-*N*-glycans with the compositions of $Hex_nHexNAc_nFuc_{0–1}$C-PA($3H^+$) or $Hex_nHexNAc_{(n+1)}Fuc_{0–1}$C-PA($3H^+$) ($n$ = 4–6) using data from LC-MS and MS/MS analysis of each neuraminidase/α1-3,4 fucosidase–digested fraction. For example, two major peaks of PA-*N*-glycans that eluted at 44.13 min (fr. 4) and 45.12 min (fr. 4) were

detected in EICs at *m/z* 817.31 [$Hex_4HexNAc_4C$-PA($3H^+$)] in several glycosidase–digested fractions, including fr. 4, 8, and 10 (Supplementary Fig. S3-9A). The MS/MS spectra of these two peaks (Supplementary Fig. S3-9B) were similar to each other, and the PA-*N*-glycan that eluted around 44.13 min (fr. 4) was identical to Standard R (2,4,2',6'-tetraantennary structure with four type II LacNAc branches, Supplementary Fig. S2) in terms of the elution time, composition, and MS/MS spectrum. This Standard R possesses β4-Gal at all four non-reducing termini, and was converted to Standard V by the action of β1-4 galactosidase. We also confirmed the removal of all four β4-Gal residues from the PA-*N*-glycan from chicken colon (Supplementary Fig. S3-10A, EIC at *m/z* 901.36 [$HexNAc_4C$-PA($2H^+$)]), but we also detected a PA-*N*-glycan with the composition $Hex_1HexNAc_4C$-PA in several fractions (fr. 4, 8, and 10) treated with neuraminidase/α1-3,4 fucosidase/β1-4 galactosidase (Supplementary Fig. S3-10A; EIC of fr. 4 at *m/z* 982.39($2H^+$) eluted around 43.04 min). By contrast, EICs at *m/z* 1063.41, 1144.44, or 1225.46 [$Hex_{2\text{–}4}HexNAc_4C$-PA($2H^+$)] of neuraminidase/α1-3,4 fucosidase/β1-4 galactosidase–treated fr. 4, 8 and 10 contained no significant ion peaks (data not shown), suggesting that PA-*N*-glycans with the composition $Hex_4HexNAc_4C$-PA lost three or four β4-Gal residues following digestion with β1-4 galactosidase. MS/MS data of the PA-*N*-glycan with composition $Hex_1HexNAc_4C$-PA that eluted at 43.04 min indicated the presence of one LacNAc branch, represented by B ion fragments at *m/z* 366, which was resistant to β1-4 galactosidase (Supplementary Fig. S3-10B). We assumed that this LacNAc branch is type I (Galβ1-3GlcNAc) rather than type II (Galβ1-4GlcNAc), and is derived from one of the two tetraantennary

PA-*N*-glycans ($Hex_4HexNAc_4C$-PA) in the neuraminidase/α1-3,4 fucosidase–digested fractions that eluted around 45.12 min (Supplementary Fig. S3-9A). This assumption was supported by the differences in ion intensities of PA-*N*-glycans. In the EICs at *m/z* 817.31 [$Hex_4HexNAc_4C$-PA($3H^+$)] of neuraminidase/α1-3,4 fucosidase–digested fr. 4 (Supplementary Fig. S3-9A), the maximum ion intensity of the PA-*N*-glycan that eluted around 44.13 min (2,4,2′,6′-tetraantennary structure with all four type II LacNAc residues, as described above) was about 2-fold higher than that of the PA-*N*-glycan that eluted around 45.12 min. Meanwhile, as shown in Supplementary Fig. S3-10A, the maximum ion intensity of the PA-*N*-glycan that eluted around 40.01 min (2,4,2′,6′-tetraantennary structure with no LacNAc sequences) in neuraminidase/α1-3,4 fucosidase/β1-4 galactosidase–treated fr. 4 was about 2-fold higher than that of the PA-*N*-glycan that eluted around 43.04 min (2,4,2′,6′-tetraantennary structure with one LacNAc). These data supported our assumption that the PA-*N*-glycan ($Hex_1HexNAc_4C$-PA) that eluted around 43.04 min was derived from the PA-*N*-glycan ($Hex_4HexNAc_4C$-PA) that eluted around 45.12 min. These two types of 2,4,2′,6′-tetraantennary structure, each with four LacNAc residues, were also detected among PA-*N*-glycans with core Fuc as shown in EICs at *m/z* 866.00 [$Hex_4HexNAc_4Fuc_1C$-PA($3H^+$)] of neuraminidase/α1-3,4 fucosidase–digested fractions including fr. 3, 4, 8, 10, and 11 (Fig. 4C, Supplementary Fig. S3-9A). For example, four peaks with the composition $Hex_4HexNAc_4Fuc_1C$-PA were detected in fr. 3 and fr. 4; the MS/MS data of these four peaks are shown in Supplementary Fig. S3-9B. Among them, PA-*N*-glycans eluted around 50.58 min and 67.46 min (fr. 4) generated B ion fragments

at *m/z* 731 ($Hex_2HexNAc_2$), suggesting the presence of LacNAc repeats. Considering its elution position, the PA-*N*-glycan that eluted around 50.58 min is probably a 2,2′,6′-triantennary structure with one LacNAc repeat. The PA-*N*-glycan that eluted around 67.46 min generated B ion fragments at *m/z* 1096 ($Hex_3HexNAc_3$) and *m/z* 731 ($Hex_2HexNAc_2$). These B ion fragments were clearly detected in MS/MS at both *m/z* 866.00 ($3H^+$) and 1298.49 ($2H^+$), suggesting the possibility of a biantennary structure with a branch containing three LacNAc units connected in tandem ($LacNAc_3$). The remaining two peaks that eluted around 54.25 and 55.01 min (fr. 4) were also found in fr. 3, 8, and 10, but only one of the two peaks was detected in fr. 11. The MS/MS spectra of these two peaks were similar to each other (Supplementary Fig. S3-9B), suggesting tetraantennary structures lacking LacNAc repeats. Considering the strong positive contribution to retention (10–11 min) made by addition of core Fuc to PA-*N*-glycans without bisecting GlcNAc, as described above, these two peaks were most likely 2,4,2′,6′-tetraantennary structures with four LacNAc residues and a core Fuc. Similar to the non-core Fuc counterparts, the one that eluted slightly earlier than the other may possess all four type II LacNAc residues, whereas the one that eluted later may possess one type I LacNAc and three type II LacNAc residues. This assumption was confirmed by LC-MS and MS/MS data from each neuraminidase/α1-3,4 fucosidase/β1-4 galactosidase–treated fraction (Supplementary Fig. S3-10A, B), as with the non-core Fuc counterparts. It should be noted that fr. 11 contained a 2,4,2′,6′-tetraantennary structure with one type I LacNAc and three type II LacNAc residues, but not all four type II LacNAc residues. Because the intact fr. 11 contains

pentasialylated tetraantennary structures with the composition $Hex_4HexNAc_4Fuc_1NeuAc_5$C-PA (Supplementary Table S1), at least one of the branches must contain two NeuAc residues. We presumed the presence of the branch sequence NeuAcα2-3Galβ1-3(NeuAcα2-6)GlcNAc, as detected in bovine fetuin *N*-glycans (e.g., Structure M in Supplementary Fig. S2), but we could not confirm this structure because too little sample was available.

The twin pairs of 2,4,2′,6′-tetraantennary structures with four LacNAc branches were also present in PA-*N*-glycans with bisecting GlcNAc (EICs at *m/z* 885.01 and 933.69 with compositions $Hex_4HexNAc_5Fuc_{0–1}$C-PA($3H^+$) after neuraminidase/α1-3,4 fucosidase digestions; Supplementary Fig. S3-11A, B). Their structures were deduced in the same way as for PA-*N*-glycans without bisecting GlcNAc, as described above. After treatment with β1-4 galactosidase, one of the tetraantennary structures lost all four β4-Gal residues ($HexNAc_5Fuc_{0–1}$C-PA in Supplementary Fig. S3-12A), whereas the other one lost three β4-Gal residues ($Hex_1HexNAc_5Fuc_{0–1}$C-PA). PA-*N*-glycans possessing 2,4,2′,6′-tetraantennary structures with one type I LacNAc and three type II LacNAc branches eluted 0.5–2.5 min later than counterparts with four type II LacNAc branches. The presence of another tetraantennary structure with bisecting GlcNAc and four LacNAc branches but a different branching pattern, which eluted around 57.19 min (fr. 4, Supplementary Fig. S3-11A, B) was suggested by EICs at *m/z* 933.69 [$Hex_4HexNAc_5Fuc_1$C-PA($3H^+$)] of the PA-*N*-glycans in neuraminidase/α1-3,4 fucosidase–treated fr. 3, 4, and 7–9. This PA-*N*-glycan lost four β4-Gal residues following digestion with β1-4 galactosidase, and eluted around 53.26 min (fr. 4, EICs at

m/z 1075.93 with the composition $HexNAc_5Fuc_1C$-PA($3H^+$) in Supplementary Fig.s S3-8A, B, and S3-12A). As described above, we deduced that this agalactosyl glycan had a 2,2′,4′,6′-tetraantennary structure with bisecting GlcNAc and core Fuc. It should be noted that this branching structure was absent from fr. 10 both before (Supplementary Fig. S3-11A) and after (Supplementary Fig. S3-12A) β1-4 galactosidase digestion. EICs at *m/z* 933.69 [$Hex_4HexNAc_5Fuc_1C$-PA($3H^+$)] of PA-*N*-glycans in neuraminidase/α1-3,4 fucosidase–treated fr. 3 and 4 also suggested the presence of several isomers with biantennary structures with LacNAc repeats (Supplementary Fig. S3-11A, B). MS/MS data indicated B ion fragments at *m/z* 731 ($Hex_2HexNAc_2$) and 1096 ($Hex_3HexNAc_3$), suggesting the possibility of biantennary structures with branches containing two or three LacNAc units connected in tandem. The presence of this tandem structure with three LacNAc was confirmed by permethylation as described below. However, due to the low ion signals in MS/MS analysis, we could not determine the accurate numbers and positions of LacNAc repeats on each branch of the isomers.

EICs of LC-MS and MS/MS analysis at *m/z* 987.71 [$Hex_5HexNAc_5Fuc_1C$-PA($3H^+$)] of PA-*N*-glycans in neuraminidase/α1-3,4 fucosidase–treated fractions suggested the presence of pentaantennary structures with LacNAc branches that eluted around 57.19 min (fr. 4) in several fractions, including fr. 3, 4, and 7–11 (Fig. 4C, Supplementary Fig. S3-13A, B). This PA-*N*-glycan should be a 2,4,2′,4′,6′-pentaantennary structure with five type II LacNAc branches, as it became the non-galactosylated 2,4,2′,4′,6′-pentaantennary PA-*N*-glycan that eluted around

52.22 min [*m/z* at 1075.93, $HexNAc_5Fuc_1C$-PA($2H^+$) in Supplementary Fig.s S3-8A, S2-12A] after β1-4 galactosidase digestion. EICs at *m/z* 987.71 of neuraminidase/α1-3,4 fucosidase–treated fr. 3, 4, 7–9, and 11 also suggested the presence of PA-*N*-glycans other than the 2,4,2′,4′,6′-pentaantennary structure, but with the same composition ($Hex_5HexNAc_5Fuc_1C$-PA), although their levels were very low. Because of the poor signal-to-noise ratio of the full MS and MS/MS spectra, it was difficult to unambiguously determine the structures of these PA-*N*-glycans. Nevertheless, we considered several putative structures based on the detection of characteristic B ion fragments of PA-*N*-glycans, as well as their relative elution positions. All PA-*N*-glycans detected in the EICs at *m/z* 987.71, except the one with a 2,4,2′,4′,6′-pentaantennary structure, generated characteristic B ion fragments at *m/z* 731, suggesting that they possess LacNAc repeat structures (Supplementary Fig. S3-13B). In the previous data, we observed positive contributions of the LacNAc repeat in the reversed-phase LC, although the ranges of contributions varied depending on the core structures and branch positions where the LacNAc repeat was added (4–9 min for PA-*N*-glycans without bisecting GlcNAc and 1.5–6 min for PA-*N*-glycans with bisecting GlcNAc). Comparison of EICs at *m/z* 987.71 (Fig. 4C, Supplementary Fig. S3-13A) and 866.00 [$Hex_4HexNAc_4Fuc_1C$-PA($3H^+$), Supplementary Fig. S3-9A] revealed that major PA-*N*-glycans with the composition $Hex_5HexNAc_5Fuc_1C$-PA, except for the one with a 2,4,2′,4′,6′-pentaantennary structure, eluted 4–6.5 min later than those with the composition $Hex_4HexNAc_4Fuc_1C$-PA. Given that we had already deduced the putative structures of PA-*N*-glycans with the composition $Hex_4HexNAc_4Fuc_1C$-PA

(Supplementary Fig. S3-9A), we deduced the structures using $Hex_5HexNAc_5Fuc_1$C-PA, which has one additional LacNAc unit in tandem. For example, the PA-*N*-glycan that eluted around 55.45 min (fr. 4, at *m/z* 987.71, $Hex_5HexNAc_5Fuc_1$C-PA) was deduced to have a 2,2′,6′-triannenaary structure with core Fuc and two LacNAc repeats, as the 2,2′,6′-triannenaary structure with core Fuc and one LacNAc repeat eluted around 50.58 min (fr. 4, at *m/z* 866.00, $Hex_4HexNAc_4Fuc_1$C-PA). The higher intensity of the signal at *m/z* 731 relative to the one at *m/z* 366 in the MS/MS analysis (fr. 4, eluted around 55.45 min in Supplementary Fig. S3-13B) suggested the presence of more than one LacNAc repeat. Given the absence of a signal at *m/z* 1096 ($Hex_3HexNAc_3$), we tentatively assumed the presence of two LacNAc repeats on different branches, but could not totally exclude the presence of isomers with a branch containing three LacNAc units connected in tandem. The PA-*N*-glycans that eluted around 59.74 min and 60.53 min (fr. 4, at *m/z* 987.71) were deduced to be 2,4,2′,6′-tetraantennary structures with core Fuc and one LacNAc repeat, as the signal at *m/z* 731 was lower than that at *m/z* 366. Because the peaks were broad, we deduced the presence of several isomers with 2,4,2′,6′-tetraantennary structures and core Fuc that differed in terms of the positions of the LacNAc repeat or the presence of type I LacNAc. The PA-*N*-glycans that eluted around 71.09, 71.75, or 73.14 min (fr. 4, at *m/z* 987.71) were probably biantennary structures with core Fuc and multiple LacNAc repeats, based on their elution position as well as the stronger signal at *m/z* 731 relative to *m/z* 366 in the MS/MS analysis (Supplementary Fig. S3-13B). Several noise signals in the MS/MS data disturbed the unambiguous structural analysis, but signals at *m/z* 1096 suggested

the presence of a branch containing three or four LacNAc units connected in tandem. We assumed that the PA-*N*-glycan that eluted around 73.14 min contained a branch with four LacNAc units connected in tandem, because the signal at *m/z* 1501, which was generated by the loss of $Hex_4HexNAc_4$, was strongest. Unfortunately, we could not confirm this assumption due to the low level of this PA-*N*-glycan.

We have already detected a 2,4,2′,4′,6′-pentaantennary structure with core Fuc and bisecting GlcNAc [*m/z* at 1177.47, $HexNAc_6Fuc_1$C-PA(2H$^+$) that eluted around 69.64 min in Supplementary Fig. S3-8A, B] in several neuraminidase/α1-3,4 fucosidase/β1-4 galactosidase–treated fractions. Therefore, we also expected the presence of five LacNAc branches to be present on the pentaantennary structure with core Fuc and bisecting GlcNAc. This was confirmed by EICs at *m/z* 1055.40 [$Hex_5HexNAc_6Fuc_1$C-PA(3H$^+$), eluted around 72.80 min (fr. 4)] of PA-*N*-glycans in several neuraminidase/α1-3,4 fucosidase–treated fractions (Supplementary Fig. S3-14A, B). The EICs at *m/z* 1055.40 also indicated the presence of isomers. Given that we had deduced the structures of isomers with the composition $Hex_5HexNAc_5Fuc_1$C-PA, as described above, we also deduced the structures of $Hex_5HexNAc_6Fuc_1$C-PA based on their elution positions and MS/MS data. All PA-*N*-glycans detected in EICs at *m/z* 1055.40, except the 2,4,2′,4′,6′-pentaantennary structure, generated characteristic B ion fragments at *m/z* 731, suggesting that they possess LacNAc repeat structures (Supplementary Fig. S3-14B). Comparing the EICs at *m/z* 1055.40 (Supplementary Fig. S3-14A) and 933.69 [$Hex_4HexNAc_5Fuc_1$C-PA(3H$^+$), Supplementary Fig. S3-11A], and considering the positive contributions of the LacNAc repeat to retention time in

reversed-phase LC, (1.5–6 min for PA-*N*-glycans with bisecting GlcNAc), the PA-*N*-glycan that eluted around 77.24, 78.38, and 79.24 min (fr. 4) were deduced to be 2,4,2′,6′-tetraantennary structures with core Fuc/bisecting GlcNAc and one LacNAc repeat. The presence of multiple peaks was probably due to the existence of several isomers of the 2,4,2′,6′-tetraantennary structure with core Fuc/bisecting GlcNAc that differed in terms of the position of the LacNAc repeat or the presence of type I LacNAc. This assumption was supported by the results of β1-4 galactosidase digestion (fr. 9, Supplementary Fig. S3-15A, B). PA-*N*-glycans possessing a 2,4,2′,6′-tetraantennary structure with core Fuc/bisecting GlcNAc and one LacNAc repeat lost four [$Hex_1HexNAc_6Fuc_1$C-PA($3H^+$) at *m/z* 839.33, eluted around 72.50 min] or three [$Hex_2HexNAc_6Fuc_1$C-PA($3H^+$) at *m/z* 893.35, eluted around 74.99 min] of the five Gal residues after the enzymatic treatment. Based on their elution position as well as the larger signals at *m/z* 731 and 1096 by MS/MS analysis (Supplementary Fig. S3-14B), the remaining two peaks in the EIC at *m/z* 1055.40 of neuraminidase/α1-3,4 fucosidase–treated fr. 4 (Supplementary Fig. S3-14A), which eluted around 83.34 and 84.88 min, were most likely biantennary structures with core Fuc/bisecting GlcNAc and multiple LacNAc repeats. These PA-*N*-glycans could be predicted to possess a branch with three or four LacNAc units connected in tandem, but we could not accurately determine the number of repetitive structures.

PA-*N*-glycans with the composition $Hex_6HexNAc_6Fuc_1$C-PA were detected in EICs at *m/z* 1109.42 ($3H^+$) of several neuraminidase/α1-3,4 fucosidase–treated fractions, including fr. 4, 7 and 9 (Fig. 4C, Supplementary Fig. S3-15A). One of the peaks, which

eluted around 76.23 min (fr. 4), was most likely a biantennary structure with a branch containing four or five LacNAc units connected in tandem, as B ion fragments at *m/z* 1462 ($Hex_4HexNAc_4$) was detected (Supplementary Fig. S3-15B). Another peak with a broad shape that eluted around 64.70 min (fr. 9) most likely represented PA-*N*-glycans possessing 2,4,2′,6′-tetraantennary structures with core Fuc and two LacNAc repeats, as they eluted 4–5.5 min later than the corresponding PA-*N*-glycans with one LacNAc repeat (Supplementary Fig. S3-13A). The breadth of the peak is probably due to the presence of several isomers, that differed in terms of the position of LacNAc repeats or the presence of type I LacNAc. This assumption is supported by the results of β1-4 galactosidase digestion (fr. 9, Supplementary Fig. S3-15A, B). The PA-*N*-glycans possessing a 2,4,2′,6′-tetraantennary structure with core Fuc and two LacNAc repeats lost four [$Hex_2HexNAc_6Fuc_1C$-PA($3H^+$) at *m/z* 893.35, eluted around 59.98 min] or three [$Hex_3HexNAc_6Fuc_1C$-PA($3H^+$) at *m/z* 947.37, eluted around 62.11 min] of the six β4-Gal residues after the enzymatic treatment.

Semi-quantitative analysis of Sia-linkages by SALSA

To discriminate α2,3- or α2,6-Sia in PA-*N*-glycans from chicken colon, we chemically modified a portion of each fraction containing sialylated PA-*N*-glycans, i.e., fr. 3–11, by SALSA, and then analyzed the sample by LC-MS and MS/MS. The elution profiles of each fraction are shown in Supplementary Fig. S4. Based on the results of full MS and MS/MS analyses, we deduced the monosaccharide compositions and Sia-linkages of each PA-*N*-glycan detected by FLD (Supplementary Table S2). Using the SALSA

method, α2,3-Sia and α2,6-Sia were alkylamidated by methylamine (MA, +13.032) and isopropylamine (iPA, +41.063), respectively, resulting in a mass difference (Δ = 28.031)[7]. The MS/MS spectra of the alkylamidated PA-*N*-glycans revealed structural features of sialylated branches via their characteristic B ion signals. For instance, MS/MS spectra at *m/z* 951.05, whose composition is $Hex_2HexNAc_3Fuc_2(NeuAc+MA)_1(NeuAc+iPA)_1C\text{-}PA(3H^+)$, contained B ion fragments at *m/z* 670 [$(NeuAc+MA)_1Hex_1HexNAc_1$], 698 [$(NeuAc+iPA)_1Hex_1HexNAc_1$], and 816 [$(NeuAc+MA)_1Hex_1HexNAc_1Fuc_1$], suggesting that fucosylated branches possess α2,3-Sia (Fig. 5A) such as sialyl Le^x [sLe^x, NeuAcα2-3Galβ1-4(Fucα1-3)GlcNAc] or sialyl Le^a [sLe^a, NeuAcα2-3Galβ1-3(Fucα1-4)GlcNAc]. Some PA-*N*-glycans from chicken colon possess sialylated LacNAc repeat structures. B ion fragments of this branch sequence were detected at *m/z* 1035 [$(NeuAc+MA)_1Hex_2HexNAc_2$], suggesting that the LacNAc repeats were α2,3-sialylated (Fig. 5B). We also found that some PA-*N*-glycans generated B ion fragments at *m/z* 1002 [$(NeuAc+MA)_1(NeuAc+iPA)_1Hex_1HexNAc_1$], suggesting the presence of one α2,3-Sia and one α2,6-Sia on the same LacNAc branch (Fig. 5C). This sequence is presumably NeuAcα2-3Galβ1-3(NeuAcα2-6)GlcNAc, as found in bovine fetuin *N*-glycans, although we could not confirm the structure.

As shown in Table S2, some of the peaks detected by the SALSA method had compositions that are not listed in Table S1. This is because larger quantities of samples were used for LC-MS with SALSA than for the analysis without SALSA. For example, we detected the compositions $Hex_{7–8}HexNAc_{7–8}Fuc_{1–2}NeuAc_{3–4}C\text{-}PA$ (pk. 6-12-1, pk.

6-12-2, pk. 9-2-1, pk. 10-8-1, pk. 10-8-2 and pk. 10-20-1) and $Hex_2HexNAc_2Fuc_1NeuAc_{0-1}NeuGc_1C$-PA (pk. 3-14-1 and pk. 5-5-2). The former are probably multiantennary structures with LacNAc repeats, and the later are likely to be PA-*N*-glycans with one NeuGc. Because we detected only trace amounts of NeuGc-containing glycans, these NeuGc residues may be derived from the diet but not biosynthesized by the chickens, as suggested previously[8].

We estimated the proportions of α2,3- and α2,6-Sia at non-reducing termini of PA-*N*-glycans from chicken colon using the peak area of each PA-*N*-glycan derivatized by the SALSA method (Fig. 5D). The results revealed that the proportions of α2,3- and α2,6-Sia in sialylated branches of PA-*N*-glycans were 72.9% and 27.1%, respectively. It should be noted that the proportions of α2,3-Sia on mono- (10.6%), di- (29.1%), tri- (21.8%), tetra- (9.9%), and penta- (1.5%) sialylated PA-*N*-glycans in chicken colon were always higher than the corresponding proportions of α2,6-Sia, regardless of the number of sialylations per PA-*N*-glycan.

Determination of linkage positions by SALSA/permethylation

Although α2,3- and α2,6-Sia-linkages on glycans can be discriminated by the SALSA method, and the major glycosidic linkages of PA-*N*-glycans from chicken colon could be determined by exoglycosidase digestions as described above, the accurate glycan sequences on branches of some glycans remained to be determined. Although the B ion fragments generated in MS/MS analysis are useful for deducing branch compositions, ion rearrangements of saccharides and functional groups often yield misleading results[9].

To solve this problem, permethylation of glycans is preferable, as this derivatization can suppress rearrangements on MS^n analysis and is useful for determining glycosidic linkages by cross-ring cleavages. We recently established a combined method in which the glycans are permethylated after SALSA[10,11]. In this study, a portion of each fraction of chicken colon PA-*N*-glycans separated on a DEAE column was subjected to SALSA/permethylation and analyzed by LC-MS, MS/MS, and MS^n. Using this method, α2,3-/α2,6-Sia-linkages and positions of other glycan modifications can be determined simultaneously. For example, we detected three isomers of PA-*N*-glycans with the composition $Hex_2HexNAc_2Fuc_1NeuAc_1(SO_3)_1$C-PA, which possess one sulfate group and one Sia, as determined by LC-MS and MS/MS analysis (pk. 6-9-1, pk. 7-3-1, and pk. 7-5-1 in Supplementary Table S1; at *m/z* 1118.89 of fr. 6 and fr. 7 in Supplementary Fig. S3-4A, B). It was initially unclear whether the sulfate group and Sia are on the same or different branches, as the sulfate group is easily transferred to different positions under ionization conditions. When we analyzed these PA-*N*-glycans by the SALSA method, we found that pk. 6-9-1 and pk. 7-5-1 in Supplementary Table S1 possessed α2,3-Sia (converted to pk. 6-5-1 and pk. 7-13-1 in Supplementary Table S2, respectively), whereas pk. 7-3-1 in Supplementary Table S1 possessed α2,6-Sia (converted to pk. 7-19-1 in Supplementary Table S2). After derivatization with SALSA/permethylation, the two PA-*N*-glycans with one α2,3-Sia and one sulfate group, whose compositions were $Hex_2HexNAc_2Fuc_1(NeuAc+MA)_1(SO_3\text{-}H+Na)_1$C-PA [at *m/z* 1410.66 ($2Na^+$)], yielded distinct MS^n spectra attributable mainly to the positions of the Sia and the sulfate group (Fig. 6). One of them exhibited B ion fragments at *m/z* 574

[$Hex_1HexNAc_1(SO_3$-H+Na$)_1(Na^+)$] and 860 [$Hex_1HexNAc_1$(NeuAc+MA$)_1(Na^+)$], suggesting that the sulfate group and Sia were located on different branches (Fig. 6A). The MS^3 spectra of the B ion fragments at *m/z* 574 exhibited B ion fragments at *m/z* 241 [$Hex_1(Na^+)$], C ion fragments at *m/z* 259 [$Hex_1(Na^+)$], B/Y ion fragments at *m/z* 356 [$HexNAc_1(SO_3$-H+Na$)_1(Na^+)$], and B/Z ion fragments at *m/z* 338 [$HexNAc_1(SO_3$-H+Na$)_1(Na^+)$], suggesting that the sulfate group was linked to GlcNAc but not Gal. Moreover, cross-ring cleavages on HexNAc at *m/z* 185, 412, and 417 (Fig. 6B) suggested that the sulfate group was linked to 6-OH of GlcNAc. One large signal at *m/z* 361 was probably generated by rearrangement of sodium sulfate (-SO_4Na) to a B ion fragments derived from Gal, as the MS^4 spectra at *m/z* 361 indicated ion fragments of Hex [*m/z* at 241 (Na^+)] and SO_4Na [*m/z* 143 (Na^+)] (Fig. 6C). By contrast, the other PA-*N*-glycans with the composition $Hex_2HexNAc_2Fuc_1$(NeuAc+MA$)_1(SO_3$-H+Na$)_1$C-PA [at *m/z* 1410.66 ($2Na^+$)] had B ion fragments at *m/z* 948 [$Hex_1HexNAc_1$(NeuAc+MA$)_1(SO_3$-H+Na$)_1(Na^+)$] and 486 [$Hex_1HexNAc_1(Na^+)$] and B/Y ion fragments at *m/z* 560 [$Hex_1HexNAc_1(SO_3$-H+Na$)_1(Na^+)$], suggesting that the sulfate group and Sia were located on the same branch (Fig. 6D). MS^3 spectra of the B/Y ion fragments at *m/z* 560 exhibited B/Y ion fragments at *m/z* 227 [$Hex_1(Na^+)$], C/Y ion fragments at *m/z* 245 [$Hex_1(Na^+)$], B/Y ion fragments at *m/z* 356 [$HexNAc_1(SO_3$-H+Na$)_1(Na^+)$], and cross-ring cleavages on HexNAc at *m/z* 185, 403 (Fig. 6E), suggesting that the sulfate group was linked to 6-OH of GlcNAc. According to the results of MS^4 analysis at *m/z* 347 (Fig. 6F), the peak at *m/z* 347 was probably generated by rearrangement of sodium

sulfate from HexNAc to B/Y ion fragments at *m/z* 227 derived from Hex, as in the case of the isomer (Fig. 6C). PA-*N*-glycans with one α2,6-Sia and one sulfate group, whose compositions were $Hex_2HexNAc_2Fuc_1(NeuAc+iPA)_1(SO_3\text{-}H+Na)_1C$-PA [at *m/z* 1424.67 ($2Na^+$) after SALSA/permethylation], showed B ion fragments at *m/z* 976 [$Hex_1HexNAc_1(NeuAc+iPA)_1(SO_3\text{-}H+Na)_1(Na^+)$] and 486 [$Hex_1HexNAc_1(Na^+)$] and B/Y ion fragments at *m/z* 560 [$Hex_1HexNAc_1(SO_3\text{-}H+Na)_1(Na^+)$], suggesting that the sulfate group and Sia were located on the same branch (data not shown).

Next, we determined the branch sequences of fucosylated and sialylated PA-*N*-glycans also after SALSA/permethylation. In fr. 3, LC-MS and MS/MS analysis revealed some multiply fucosylated PA-*N*-glycans with the compositions $Hex_2HexNAc_3Fuc_{2\text{–}3}NeuAc_1C$-PA (Supplementary Table S1, Supplementary Fig. S3-3). The PA-*N*-glycan with the composition $Hex_2HexNAc_3Fuc_2NeuAc_1C$-PA (pk. 3-16-1 in Supplementary Table S1) was converted to $Hex_2HexNAc_3Fuc_2(NeuAc+iPA)_1C$-PA by SALSA (pk. 3-28-1 in Supplementary Table S2), suggesting that it contained α2,6-Sia. At this point, it was uncertain whether Fuc and α2,6-Sia were on the same or different branches, as Fuc on branches is easily transferred to different positions by ionization. After SALSA/permethylation, MS/MS analysis of the PA-*N*-glycan with this composition [*m/z* 1067.88 ($3Na^+$)] revealed B ion fragments at *m/z* 660 [$Hex_1HexNAc_1Fuc_1(Na^+)$] and 888 [$Hex_1HexNAc_1(NeuAc+iPA)_1(Na^+)$], suggesting that the Fuc and α2,6-Sia were on different branches (Supplementary Fig. S5-1A). The Y ion fragments at *m/z* 566 [$Fuc_1HexNAc_1$-PA(Na^+)] supported the presence of core Fuc. The MS^3 spectra of the B ion fragments at *m/z* 660 exhibited B ion fragments at

m/z 241 [$Hex_1(Na^+)$], C ion fragments at *m/z* 259 [$Hex_1(Na^+)$], B/Y ion fragments at *m/z* 442 [$HexNAc_1Fuc_1(Na^+)$], and C/Y ion fragments at *m/z* 424 [$HexNAc_1Fuc_1(Na^+)$], suggesting that Fuc is linked to GlcNAc. Furthermore, cross-ring cleavages on HexNAc at *m/z* 329 and 586 (Supplementary Fig. S5-1B) suggested that Fuc and Hex were linked to 3- and 4-OH of HexNAc, respectively. Together with the results of neuraminidase, α1-3,4 fucosidase, and β1-4 galactosidase digestions, these observations indicate that the sequence of this fucosylated branch is Le^x (Galβ1-4(Fucα1-3)GlcNAc).

On the other hand, two PA-*N*-glycans with the composition $Hex_2HexNAc_3Fuc_3NeuAc_1$C-PA (pk. 3-14-3 and pk. 3-16-3 in Supplementary Table S1) were converted to $Hex_2HexNAc_3Fuc_3(NeuAc{+}MA)_1$C-PA by SALSA (pk. 3-8-3 and pk. 3-10-2 in Supplementary Table S2), suggesting that both of them contained α2,3-Sia. After SALSA/permethylation, MS/MS analysis of the PA-*N*-glycan with this composition [*m/z* 1116.56 ($3Na^+$)] revealed B ion fragments at *m/z* 660 [$Hex_1HexNAc_1Fuc_1(Na^+)$] and 1035 [$Hex_1HexNAc_1Fuc_1(NeuAc{+}MA)_1(Na^+)$], suggesting that one of the two fucosylated branches possessed α2,3-Sia (Supplementary Fig. S5-2A). The Y ion fragments at *m/z* 566 [$Fuc_1HexNAc_1$-$PA(Na^+)$] supported the presence of core Fuc. The MS^3 spectrum of the B/Y ion fragments at *m/z* 646 [$Hex_1HexNAc_1Fuc_1(Na^+)$] exhibited B/Y ion fragments at *m/z* 227 [$Hex_1(Na^+)$], C/Y ion fragments at *m/z* 245 [$Hex_1(Na^+)$], B/Y ion fragments at *m/z* 442 [$HexNAc_1Fuc_1(Na^+)$], C/Y ion fragments at *m/z* 424 [$HexNAc_1Fuc_1(Na^+)$], and cross-ring cleavages on HexNAc at *m/z* 315 and 572 (Supplementary Fig. S5-2B),

suggesting that Fuc and Hex are linked to 3- and 4-OH of HexNAc, respectively. Together with the results of neuraminidase, α1-3,4 fucosidase, and β1-4 galactosidase digestions, we concluded that the sequence of this sialylated and fucosylated branch is sLe^x.

PA-*N*-glycans with the composition $Hex_1HexNAc_3Fuc_1C$-PA [*m/z* 953.87 ($2H^+$)] were found in fr. 1. One of them was assumed to possess LacdiNAc, as the MS/MS spectrum indicated characteristic B ion fragments at *m/z* 407 ($HexNAc_2$) (pk. 1-27-4 in Supplementary Table S1). This was confirmed by MS^n analysis of the permethylated PA-*N*-glycan [*m/z* 1200.11 ($2Na^+$)], which showed B ion fragments at *m/z* 527 [$HexNAc_2(Na^+)$] (Supplementary Fig. S5-3A). The MS^3 spectra of the B ion fragments at *m/z* 527 indicated cross-ring cleavages on HexNAc of the reducing side at *m/z* 180, 370, and 453 (Supplementary Fig. S5-3B), suggesting that one HexNAc (probably GalNAc) is linked to 4-OH of HexNAc (probably GlcNAc). Although it would be preferable to identify the type of each monosaccharide, we assumed that the branches possessed the conventional LacdiNAc (GalNAcβ1-4GlcNAc) sequence, which is commonly found in vertebrates.

When we analyzed PA-*N*-glycans by LC-MS and MS/MS before and after exoglycosidase digestions, we found several PA-*N*-glycans with extended branches containing two to five LacNAc units connected in tandem, as described in the previous subsection. For example, one of the PA-*N*-glycans with the composition $Hex_3HexNAc_4Fuc_1NeuAc_1C$-PA in fr. 3 (pk. 3-31-1 in Supplementary Table S1) seemed to have a branch with two LacNAc units connected in tandem, as the B ion

fragments at *m/z* 731 ($Hex_2HexNAc_2$) and 1022 ($Hex_2HexNAc_2NeuAc_1$) were clearly detected by MS/MS analysis. Using the SALSA method, this PA-*N*-glycan was converted to $Hex_3HexNAc_4Fuc_1(NeuAc+MA)_1C$-PA (pk. 3-25-2 in Supplementary Table S2), suggesting the presence of α2,3-Sia. After SALSA/permethylation, MS/MS analysis of the PA-*N*-glycan with this composition [*m/z* 1150.24 ($3Na^+$)] revealed B ion fragments at *m/z* 486 [$Hex_1HexNAc_1(Na^+)$] and 1309 [$Hex_2HexNAc_2(NeuAc+MA)_1(Na^+)$], B/Y ion fragments at *m/z* 921 [$Hex_2HexNAc_2(Na^+)$], and Y ion fragments at *m/z* 1071 [$Hex_1HexNAc_2Fuc_1C$-PA($2Na^+$)], suggesting the presence of two LacNAc units connected in tandem (Supplementary Fig. S5-4A). MS^3 spectra of the B/Y ion fragments at *m/z* 921 exhibited B/Y ion fragments at *m/z* 472 [$Hex_1HexNAc_1(Na^+)$] and 717 [$Hex_1HexNAc_2(Na^+)$], confirming the LacNAc repeat structure (Supplementary Fig. S5-4B). Furthermore, $^{3,5}A$ cross-ring cleavages of HexNAc (*m/z* 315 and 764) suggested that Hex (probably Gal) was linked to 4- or 6-OH of HexNAc (probably GlcNAc). Together with the results of β1-4 galactosidase digestion, these observations indicated that the sequence of this LacNAc unit was most likely type II (Galβ1-4GlcNAc). We could not determine the linkage between two LacNAc residues, i.e., HexNAc-Hex, because the glycosidic linkage of HexNAc-Hex is easily cleaved by CID. One possible linkage for this position is GlcNAcβ1-3Gal, which is common in vertebrates.

Similarly, LC-MS and MS/MS analyses of SALSA/permethylated PA-*N*-glycans in fr. 3 with the composition $Hex_4HexNAc_5Fuc_1(NeuAc+MA)_1C$-PA [*m/z* 1299.99

$(3Na^{+})$] clearly distinguished between PA-*N*-glycans with a LacNAc repeat and those with multiantennary structures (Supplementary Fig. S5-5). One of the MS/MS spectra of SALSA/permethylated PA-*N*-glycans with this composition indicated B ion fragments at *m/z* 486 [$Hex_1HexNAc_1(Na^{+})$] and 1759 [$Hex_3HexNAc_3(NeuAc+MA)_1(Na^{+})$], B/Y ion fragments at *m/z* 1371 [$Hex_2HexNAc_2(Na^{+})$], and Y ion fragments at *m/z* 1071 [$Hex_1HexNAc_2Fuc_1C$-PA$(2Na^{+})$], suggesting the presence of α2,3-sialylated $LacNAc_3$ (Supplementary Fig. S5-5A). By contrast, another SALSA/permethylated PA-*N*-glycans with the same composition generated Y/Y ion fragments at *m/z* 991/1475 [$Hex_2HexNAc_3Fuc_1(NeuAc+MA)_1C$-PA$(3Na^{+}/2Na^{+})$], suggesting a multiantennary structure (Supplementary Fig. S5-5B). Although the detailed structures of some minor PA-*N*-glycans containing such LacNAc repeats could not be further analyzed due to insufficient quantities of sample, most of them were expected to possess cognate repeating structures.

Summary of the structural features of *N*-glycans in chicken colon

Based on the results of LC-MS, MS/MS, exoglycosidase digestions, SALSA, and SALSA/permethylation, we deduced the structures of almost all major PA-*N*-glycans from chicken colon, including the core structures, branching patterns, and branch sequences, with the exception of ambiguous positions of asymmetric branches, Sia-linkages, and LacNAc linkages (i.e., type I or type II) on each glycan. The deduced structures are summarized in Supplementary Table S1, along with the relative amounts

calculated from the area of each peak detected by fluorescence and full MS. Using the data sets, we calculated the contents of categorized glycan structures (Fig. 7). For instance, about one-third of *N*-glycans in chicken colon were of the high mannose-type, as is common in animal tissues (Fig. 7A). Biantennary structures were the most abundant among complex-type *N*-glycans, and 2,2′,6′-triantennary, 2,4′,2′-triantennary, and 2,4,2′,6′-tetraantennary structures were almost equally abundant. Small amounts of pentaantennary structures (0.4% in total *N*-glycans) were also detected. Meanwhile, *N*-glycans with 2,2′,4′,6′-tetraantennary structures could not be detected before neuraminidase digestion, although they could be detected after exoglycosidase digestions (Supplementary Fig.s S3-8, 3-11). About a half of all *N*-glycans possessed core Fuc with or without bisecting GlcNAc, and about a half of complex or hybrid-type *N*-glycans possessed bisecting GlcNAc with or without core Fuc (Fig. 7B). About 30% of all *N*-glycans were sialylated, with disialylation being most prevalent among the sialylated *N*-glycans (Fig. 7C). *N*-Glycans possessing at least one sLe^x constituted 2.5% of total *N*-glycans (data not shown). To quantify the structural features of branch sequences, we calculated the amounts of each GlcNAc/LacNAc/LacdiNAc-containing branch on complex and hybrid-type *N*-glycans (Fig. 7D). Although more than 90% of the branches were simple GlcNAc, LacNAc, or sialyl LacNAc (sLacNAc) sequences, some minor sequences such as Le^x, sLe^x, sulfated LacNAc, LacdiNAc, and LacNAc repeat were characteristic structures of chicken colon *N*-glycans.

References in *Supplementary Results*

1. Tomiya, N. *et al.* Analyses of *N*-linked oligosaccharides using a two-dimensional mapping technique. *Anal. Biochem.* **171**, 73-90 (1988).
2. Natsuka, S., Masuda, M., Sumiyoshi, W. & Nakakita, S. Improved method for drawing of a glycan map, and the first page of glycan atlas, which is a compilation of glycan maps for a whole organism. *PLoS One* **9**, e102219, doi:10.1371/journal.pone.0102219 (2014).
3. Tomiya, N. & Takahashi, N. Contribution of component monosaccharides to the coordinates of neutral and sialyl pyridylaminated *N*-glycans on a two-dimensional sugar map. *Anal. Biochem.* **264**, 204-210 (1998).
4. Wuhrer, M., Koeleman, C. A., Hokke, C. H. & Deelder, A. M. Mass spectrometry of proton adducts of fucosylated *N*-glycans: fucose transfer between antennae gives rise to misleading fragments. *Rapid Commun. Mass Spectrom.* **20**, 1747-1754, doi:10.1002/rcm.2509 (2006).
5. Hanzawa, K., Suzuki, N. & Natsuka, S. Structures and developmental alterations of *N*-glycans of zebrafish embryos. *Glycobiology* **27**, 228-245, doi:10.1093/glycob/cww124 (2017).
6. Yamashita, K., Kamerling, J. P. & Kobata, A. Structural study of the carbohydrate moiety of hen ovomucoid. Occurrence of a series of pentaantennary complex-type asparagine-linked sugar chains. *J. Biol. Chem.* **257**, 12809-12814 (1982).
7. Nishikaze, T. *et al.* Differentiation of sialyl linkage isomers by one-pot sialic acid derivatization for mass spectrometry-based glycan profiling. *Anal. Chem.* **89**, 2353-2360, doi:10.1021/acs.analchem.6b04150 (2017).
8. Schauer, R., Srinivasan, G. V., Coddeville, B., Zanetta, J. P. & Guerardel, Y. Low incidence of *N*-glycolylneuraminic acid in birds and reptiles and its absence in the platypus. *Carbohydr. Res.* **344**, 1494-1500 (2009).
9. Wuhrer, M., Deelder, A. M. & van der Burgt, Y. E. Mass spectrometric glycan rearrangements. *Mass Spectrom. Rev.* **30**, 664-680, doi:10.1002/mas.20337 (2011).
10. Suzuki, N., Abe, T. & Natsuka, S. Quantitative LC-MS and MS/MS analysis of sialylated glycans modified by linkage-specific alkylamidation. *Anal. Biochem.*

567, 117-127, doi:10.1016/j.ab.2018.11.014 (2019).
11. Abe, T., Kameyama, A., Natsuka, S. & Suzuki, N. Sequential modifications of glycans by linkage-specific alkylamidation of sialic acids and permethylation. *Anal. Biochem.* **606**, 113861, doi:10.1016/j.ab.2020.113861 (2020).
