# Supplementary material for: Toward robust N-glycomics of various tissue samples that may contain glycans with unknown or unexpected structures
Source: Sci Rep. 2021 Mar 18;11:6334. doi: 10.1038/s41598-021-84668-x (PMC7973440; doi:10.1038/s41598-021-84668-x)
Supplement: Supplementary file 3 — Supplementary Information 3. [file 41598_2021_84668_MOESM3_ESM.pdf]

Table S1. MS and MS/MS data for PA-*N*-glycans from chicken colon on LC-MS

a) H, hexose; HN, *N*-acetylhexosamine; F, fucose (or deoxyhexose); GN, *N*-acetylglucosamine; M, mannose; NA, *N*-acetylneuraminic acid; SO3, sulfate group; HPO3, phosphate group; C, trimannosyl core; PA, 2-aminopyridine.

b) Monosaccharide symbols for deduced glycan structures were as follows:

: Man : Gal : Glc : GlcNAc : GalNAc : Fuc : NeuAc : sulfate group : phosphate group

c) Most of the detectable fluorescence peaks eluted in 10–87 min were numbered, but not all peaks were derived from PA-*N*-glycans. The compositions of some peaks could not be determined by MS and MS/MS analysis due to insufficent signals; these cases are indicated as "data not available".

d) Individual peaks detected by fluorescence sometimes included more than two kinds of PA-glycans with different mass values. In such cases, the proportions were estimated using the ratios of integrated ion intensities for each *m/z* value detected at the corresponding elution time.

e) Amounts of glycans relative to the most abundant glycan (pk.1-14-1), for which the amount was defined as 100.

f) xMS, MS data not available; xMS2, MS/MS data not available.

Fr. No. (DEAE)	Peak. No. (ODS)	Full MS No.	Elusion time max (min)	Elusion time range (min)	Observed parent ion (*m/z* value)	Calculated (*m/z* value)	Estimated adduct	Estimated composition[a), c)]	Deduced glycan structure[b)]	Characteristic fragments[a)]	Relative amounts[d)]	Notes[f)]
fr.1	pk.1-1	1	14.96	14.63-15.19	899.85	900.33	M+2H$^+$	H5C-PA (M8GN2-PA)	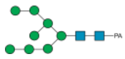	325(H2)	0.78	artifact (Shiff-base by incomplete reduction, pk. 1-6-1)
	pk.1-2	1	15.54	15.33-15.75	818.73	819.31	M+2H$^+$	H4C-PA (M7GN2-PA)	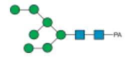	325(H2)<br>1151(H4HN2-PA)<br>1314(H5HN2-PA)	0.33	epimer (pk.1-7-1)
		2			979.91	981.36	M+2H$^+$	H6C-PA (M9GN2-PA)	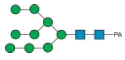	325(H2)	0.15	artifact (Shiff-base by incomplete reduction, pk.1-8-1)
	pk.1-3	1	16.03	15.82-16.59	980.89	981.36	M+2H$^+$	H6C-PA (M9GN2-PA)	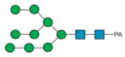	325(H2)<br>1151(H4HN2-PA)<br>1314(H5HN2-PA)	1.24	epimer (pk.1-8-1)
	pk.1-4	1	18.09	17.70-18.47	737.77<br>1475.55	738.28<br>1475.55	M+2H$^+$<br>M+H$^+$	H3C-PA (M6GN2-PA)	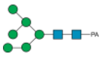	325(H2)<br>1151(H4HN2-PA)	0.93	epimer (pk.1-11-1)
		2			1142.83			data not available			0.11	(artifact)
	pk.1-5	1	21.52	20.98-21.95	656.81<br>1313.43	657.25<br>1313.50	M+2H$^+$<br>M+H$^+$	H2C-PA (M5GN2-PA)	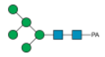	325(H2)<br>1151(H4HN2-PA)	1.62	epimer (pk.1-14-1)
	pk.1-6	1	23.53	23.00-23.97	900.06<br>1800.48	900.33<br>1799.66	M+2H$^+$<br>M+H$^+$	H5C-PA (M8GN2-PA)	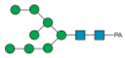	325(H2)<br>1151(H4HN2-PA)<br>1314(H5HN2-PA)	42.62	
	pk.1-7	1	24.70	24.18-25.23	818.86<br>1637.48	819.31<br>1637.60	M+2H$^+$<br>M+H$^+$	H4C-PA (M7GN2-PA)	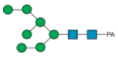	325(H2)<br>1151(H4HN2-PA)<br>1314(H5HN2-PA)	24.34	
	pk.1-8	1	25.94	25.37-26.55	981.27<br>1962.56	981.36<br>1961.71	M+2H$^+$<br>M+H$^+$	H6C-PA (M9GN2-PA)	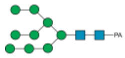	325(H2)<br>1151(H4HN2-PA)<br>1314(H5HN2-PA)	66.00	
	pk.1-9	1	27.34	26.83-27.60	899.86	900.33	M+2H$^+$	H5C-PA (M8GN2-PA)	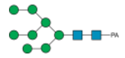	325(H2)<br>1151(H4HN2-PA)<br>1314(H5HN2-PA)	3.58	
	pk.1-10	1	28.07	27.67-28.57	818.87	819.31	M+2H$^+$	H4C-PA (M7GN2-PA)	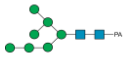	325(H2)<br>1151(H4HN2-PA)<br>1314(H5HN2-PA)	7.17	
		2			737.82	738.28	M+2H$^+$	H3C-PA (M6GN2-PA)	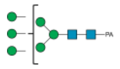	325(H2)<br>1151(H4HN2-PA)	1.91	
		3			912.75	912.85	M+2H$^+$	H3HN1F1C-PA	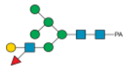	366(H1HN1)<br>512(H1HN1F1)<br>1151(H4HN2-PA)<br>1314(H5HN2-PA)	1.22	Le^X
	pk.1-11	1	29.20	28.78-30.04	738.10<br>1475.48	738.28<br>1475.55	M+2H$^+$<br>M+H$^+$	H3C-PA (M6GN2-PA)	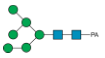	325(H2)<br>1151(H4HN2-PA)<br>1314(H5HN2-PA)	57.93	
	pk.1-12	1	31.23	30.73-31.43	997.26			data not available			2.16	(artifact)

Table S1 Continued.

Fr. No. (DEAE)	Peak. No. (ODS)	Full MS No.	Elusion time max (min)	Elusion time range (min)	Observed parent ion (*m/z* value)	Calculated (*m/z* value)	Estimated adduct	Estimated composition[a), c)]	Deduced glycan structure[b)]	Characteristic fragments[a)]	Relative amounts[d)]	Notes[f)]
		2			899.80	900.33	$M+2H^+$	H5C-PA (M8GN2-PA)	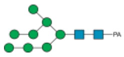	325(H2)<br>1151(H4HN2-PA)<br>1314(H5HN2-PA)	0.59	
		3			860.22	860.33	$M+2H^+$	H2HN2C-PA	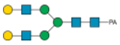	366(H1HN1)<br>1192(H3HN3-PA)	0.32	epimer<br>(pk.1-19-1)
	pk.1-13	1	31.94	31.50-32.13	1062.04	1062.39	$M+2H^+$	H7C-PA (Glc1M9GN2-PA)	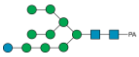	325(H2)<br>1151(H4HN2-PA)<br>1314(H5HN2-PA)	2.69	
	pk.1-14	1	33.17	32.20-34.01	656.99<br>1313.47	657.25<br>1313.50	$M+2H^+$<br>$M+H^+$	H2C-PA (M5GN2-PA)	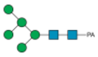	325(H2)<br>1151(H4HN2-PA)	100.00	
	pk.1-15	1	35.38	34.84-36.24	840.30<br>1678.51	839.82<br>1678.63	$M+2H^+$<br>$M+H^+$	H3HN1C-PA	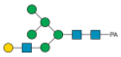	366(H1HN1)<br>1151(H4HN2-PA)<br>1314(H5HN2-PA)	12.94	
		2			758.84<br>1516.54	758.79<br>1516.58	$M+2H^+$<br>$M+H^+$	H2HN1C-PA	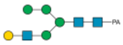	366(H1HN1)<br>1151(H4HN2-PA)	5.85	
	pk.1-16	1	36.95	36.38-37.70	738.04	738.28	$M+2H^+$	H3C-PA (M6GN2-PA)	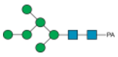	325(H2)<br>1151(H4HN2-PA)	1.63	
		2			1107.90	1107.93	$M+2H^+$	H2HN3F2C-PA	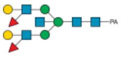	366(H1HN1)<br>512(H1HN1F1)	0.79	Le^X
		3			920.74	920.85	$M+2H^+$	H4HN1C-PA	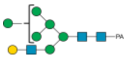	1314(H5HN2-PA)<br>1476(H6HN2-PA)	0.59	
	pk.1-17	1	39.09	38.54-40.14	860.30	860.33	$M+2H^+$	H2HN2C-PA	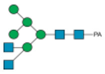	1151(H4HN2-PA)<br>1314(H5HN2-PA)	14.44	
	pk.1-18	1	41.87	41.05-42.44	779.78	779.31	$M+2H^+$	H1HN2C-PA	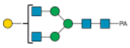	366(H1HN1)<br>1192(H3HN3-PA)	4.28	
		2			933.88	933.36	$M+2H^+$	H2HN2F1C-PA	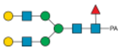	366(H1HN1)<br>1135(F1C-PA)<br>1339(H3HN3F1-PA)	2.66	epimer<br>(pk.1-27-1)
		3			941.81	941.36	$M+2H^+$	H3HN2C-PA	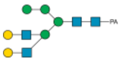	366(H1HN1)<br>1151(H4HN2-PA)	3.98	
	pk.1-19	1	43.86	43.35-44.11	860.44	860.33	$M+2H^+$	H2HN2C-PA	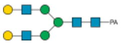	366(H1HN1)<br>1192(H3HN3-PA)	12.57	Standard E
	pk.1-20	1	44.72	44.18-45.51	973.37	973.40	$M+H^+$	H2HN2F1-PA	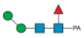	446(F1HN1-PA)	12.20	
		2			1135.41	1135.45	$M+2H^+$	F1C-PA	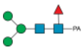	446(F1HN1-PA)	6.84	
		3			1063.38	1063.41	$M+2H^+$	H2HN4C-PA	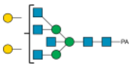	366(H1HN1)	2.43	
		4			1115.88	1115.93	$M+2H^+$	H3HN3F1C-PA	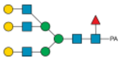	366(H1HN1)<br>446(F1HN1-PA)<br>1339(H3HN3F1-PA)	4.59	

Table S1 Continued.

Fr. No. (DEAE)	Peak. No. (ODS)	Full MS No.	Elusion time max (min)	Elusion time range (min)	Observed parent ion (*m/z* value)	Calculated (*m/z* value)	Estimated adduct	Estimated composition[a), c)]	Deduced glycan structure[b)]	Characteristic fragments[a)]	Relative amounts[d)]	Notes[f)]
		5			912.63	912.85	$M+2H^{+}$	H3HN1F1C-PA	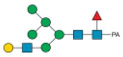	366(H1HN1)<br>1135(F1C-PA)	1.68	
		6			1022.41	1022.39	$M+2H^{+}$	H4HN2C-PA	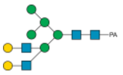	366(H1HN1)	1.37	
	pk.1-21	1	45.87	45.58-46.27	860.31	860.33	$M+2H^{+}$	H2HN2C-PA	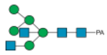	366(H1HN1)<br>1151(H4HN2-PA)	6.87	
	pk.1-22	1	46.74	46.41-47.25	832.32	831.82	$M+2H^{+}$	H2HN1F1C-PA	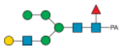	366(H1HN1)<br>973(H2HN2F1-PA)<br>1135(F1C-PA)	2.99	
		2			750.76	750.80	$M+2H^{+}$	H1HN1F1C-PA	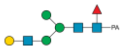	366(H1HN1)<br>973(H2HN2F1-PA)<br>1135(F1C-PA)	1.97	
		3			1144.41	1144.44	$M+2H^{+}$	H3HN4C-PA	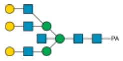	366(H1HN1)	0.98	
	pk.1-23	1	48.00	47.32-48.92	787.81<br>1181.43	787.64<br>1180.96	$M+3H^{+}$<br>$M+2H^{+}$	H2HN3F3C-PA	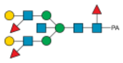	366(H1HN1)<br>446(F1HN1-PA)<br>512(H1HN1F1)<br>1339(H3HN3F1-PA)	20.30	Le^{X}
	pk.1-24	1	50.09	49.41-50.80	771.28	771.31	$M+2H^{+}$	HN2F1C-PA	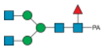	973(H2HN2F1-PA)<br>1135(F1C-PA)	8.36	Standard J
	pk.1-25	1	51.39	50.94-51.85	953.86	953.87	$M+2H^{+}$	H1HN3F1C-PA	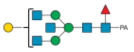	366(H1HN1)<br>446(F1HN1-PA)<br>1339(H3HN3F1-PA)	1.71	epimer<br>(pk.1-32-1, 1-33-1)
		2			839.91	839.82	$M+2H^{+}$	H3HN1C-PA	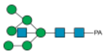	1314(H5HN2-PA)<br>1476(H6HN2-PA)	0.83	
		3			1001.83			data not available			1.05	(artifact)
	pk.1-26	1	52.37	51.99-53.10	852.82	852.33	$M+2H^{+}$	H1HN2F1C-PA	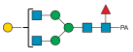	366(H1HN1)<br>973(H2HN2F1-PA)<br>1135(F1C-PA)<br>1339(H3HN3F1-PA)	5.06	
		2			1055.38	1055.41	$M+2H^{+}$	H1HN4F1C-PA	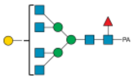	366(H1HN1)<br>1339(H3HN3F1-PA)	0.85	
	pk.1-27	1	54.30	53.59-55.82	933.96<br>1865.54	933.36<br>1865.72	$M+2H^{+}$<br>$M+H^{+}$	H2HN2F1C-PA	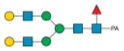	366(H1HN1)<br>973(H2HN2F1-PA)<br>1135(F1C-PA)<br>1339(H3HN3F1-PA)	43.71	Standard F
		2			799.82	799.82	$M+2H^{+}$	HN3C-PA	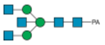	1030(H2HN3-PA)	13.16	Standard K
		3			1136.35	1136.44	$M+2H^{+}$	H2HN4F1C-PA	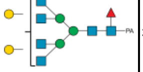	366(H1HN1)	4.40	
		4			954.42	953.87	$M+2H^{+}$	H1HN3F1C-PA	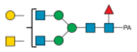	366(H1HN1)<br>407(HN2)<br>446(F1HN1-PA)<br>1135(F1C-PA)<br>1339(H3HN3F1-PA)	2.95	LacdiNAc
	pk.1-28	1	57.94	56.52-58.61	880.82	880.85	$M+2H^{+}$	H1HN3C-PA	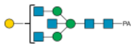	366(H1HN1)	17.50	

Table S1 Continued.

Fr. No. (DEAE)	Peak. No. (ODS)	Full MS No.	Elusion time max (min)	Elusion time range (min)	Observed parent ion (*m/z* value)	Calculated (*m/z* value)	Estimated adduct	Estimated composition[a), c)]	Deduced glycan structure[b)]	Characteristic fragments[e)]	Relative amounts[d)]	Notes[f)]
		2			1217.86	1217.47	M+2H$^+$	H3HN4F1C-PA	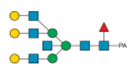	366(H1HN1)<br>1542(H3HN4F1-PA)	6.31	
		3			1055.27	1055.41	M+2H$^+$	H1HN4F1C-PA	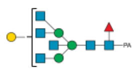	366(H1HN1)<br>1542(H3HN4F1-PA)	2.89	
		4			1026.60	1026.90	M+2H$^+$	H1HN3F2C-PA	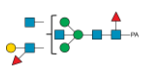	366(H1HN1)<br>512(H1HN1F1)<br>1339(H3HN3F1-PA)	1.81	Le^x
	pk.1-29	1	59.52	58.82-59.93	933.36	933.36	M+2H$^+$	H2HN2F1C-PA	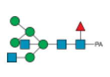	1135(F1C-PA)<br>1339(H3HN3F1-PA)	4.26	
		2			1107.87	1107.93	M+2H$^+$	H2HN3F2C-PA	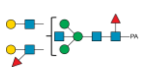	366(H1HN1)<br>512(H1HN1F1)<br>1339(H3HN3F1-PA)<br>1542(H3HN4F1-PA)	0.75	Le^x
		3			974.39	974.39	M+2H$^+$	HN4F1C-PA	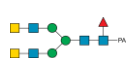	407(HN2)<br>1339(H3HN3F1-PA)<br>1542(H3HN4F1-PA)	0.75	LacdiNAc
	pk.1-30	1	60.57	60.07-61.33	962.27	961.87	M+2H$^+$	H2HN3C-PA	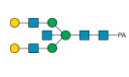	366(H1HN1)<br>1192(H3HN3-PA)	16.67	Standard G
	pk.1-31	1	69.08	68.16-69.90	872.86	872.85	M+2H$^+$	HN3F1C-PA	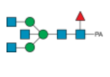	1135(F1C-PA)<br>1339(H3HN3F1-PA)	19.73	Standard L
	pk.1-32	1	71.33	70.39-71.85	954.28	953.87	M+2H$^+$	H1HN3F1C-PA	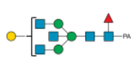	366(H1HN1)<br>1339(H3HN3F1-PA)	15.75	
	pk.1-33	1	72.53	71.99-73.17	636.27<br>953.86	636.25<br>953.87	M+2H$^+$	H1HN3F1C-PA	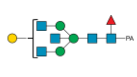	366(H1HN1)<br>1339(H3HN3F1-PA)	22.98	
	pk.1-34	1	74.22	73.3175.05	690.60<br>1035.11	690.27<br>1034.90	M+3H$^+$<br>M+2H$^+$	H2HN3F1C-PA	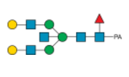	366(H1HN1)<br>446(F1HN1-PA)<br>1135(F1C-PA)<br>1339(H3HN3F1-PA)	85.18	Standard H
	pk.1-35	1	76.58	75.82-77.01	744.25<br>1116.30	744.29<br>1115.93	M+3H$^+$<br>M+2H$^+$	H3HN3F1C-PA	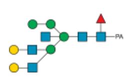	366(H1HN1)<br>446(F1HN1-PA)<br>1135(F1C-PA)<br>1339(H3HN3F1-PA)	2.81	
		2			1056.04	1055.41	M+2H$^+$	H1HN4F1C-PA	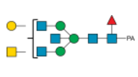	366(H1HN1)<br>407(HN2)	0.21	LacdiNAc
		3			974.61	974.39	M+2H$^+$	HN4F1C-PA	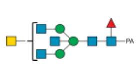	407(HN2)<br>1339(H3HN3F1-PA)<br>1542(H3HN4F1-PA)	0.54	LacdiNAc
	pk.1-36	1	77.64	77.15-78.47	1180.48	1180.45	M+2H$^+$	H2HN3F1NA1C-PA	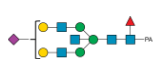	366(H1HN1)<br>657(H1HN1NA1)<br>1339(H3HN3F1-PA)<br>1542(H3HN4F1-PA)	1.53	NA (pk.3-27-1)
		2			1055.96	1055.41	M+2H$^+$	H1HN4F1C-PA	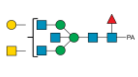	366(H1HN1)<br>407(HN2)<br>1339(H3HN3F1-PA)	1.28	LacdiNAc
	pk.1-37	1	79.79	79.31-80.63	909.16<br>1363.14	909.01<br>1363.01	M+3H$^+$<br>M+2H$^+$	H3HN4F1NA1C-PA	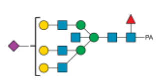	366(H1HN1)<br>657(H1HN1NA1)<br>1339(H3HN3F1-PA)<br>1542(H3HN4F1-PA)	1.34	NA (pk.3-29-2)
		2			811.98<br>1217.76	811.98<br>1217.47	M+3H$^+$<br>M+2H$^+$	H3HN4F1C-PA	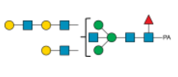	366(H1HN1)<br>731(H2HN2)<br>1339(H3HN3F1-PA)<br>1542(H3HN4F1-PA)	1.28	LacNAc repeat

Table S1 Continued.

Fr. No. (DEAE)	Peak. No. (ODS)	Full MS No.	Elusion time max (min)	Elusion time range (min)	Observed parent ion (*m/z* value)	Calculated (*m/z* value)	Estimated adduct	Estimated composition[a), c)]	Deduced glycan structure[b)]	Characteristic fragments[a)]	Relative amounts[d)]	Notes[f)]
	pk.1-38	1	85.44	85.17-85.70	811.99<br>1217.80	811.98<br>1217.47	$M+3H^+$<br>$M+2H^+$	H3HN4F1C-PA	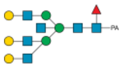	366(H1HN1)<br>1339(H3HN3F1-PA)<br>1542(H3HN4F1-PA)	0.52	
fr.3	pk.3-1	1	18.14	17.75-18.80	879.43	879.80	$M+2H^+$	H3HN1(HPO3)1C-PA	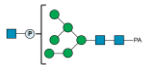	405(H2(HPO3)1)<br>1151(H4HN2-PA)	2.23	HPO3
	pk.3-2	1	19.31	18.94-19.71	1041.51	1041.86	$M+2H^+$	H5HN1(HPO3)1C-PA	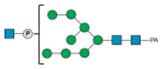	405(H2(HPO3)1)<br>1151(H4HN2-PA)	0.76	HPO3
	pk.3-3	1	20.24	19.85-20.96	960.50	960.83	$M+2H^+$	H4HN1(HPO3)1C-PA	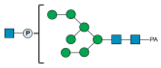	405(H2(HPO3)1)<br>1151(H4HN2-PA)	0.59	HPO3
	pk.3-4	1	23.24	22.71-23.69	879.32	879.80	$M+2H^+$	H3HN1(HPO3)1C-PA	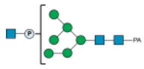	405(H2(HPO3)1)	0.23	HPO3
	pk.3-5	1	33.22	36.34-38.71	1006.20	1005.88	$M+2H^+$	H2HN2NA1C-PA	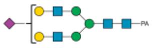	366(H1HN1)<br>657(H1HN1NA1)<br>1192(H3HN3-PA)	0.41	epimer<br>(pk.3-10-1)
	pk.3-6	1	37.53	36.34-38.71	985.36	985.37	$M+2H^+$	H3HN1NA1C-PA	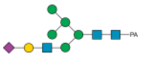	366(H1HN1)<br>657(H1HN1NA1)<br>1151(H4HN2-PA)<br>1314(H5HN2-PA)	1.37	
		2			823.27	823.31	$M+2H^+$	H1HN1NA1C-PA	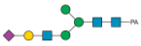	366(H1HN1)<br>657(H1HN1NA1)<br>989(H3HN2-PA)	0.45	
		3			904.33	904.34	$M+2H^+$	H2HN1NA1C-PA	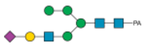	366(H1HN)<br>657(H1HN1NA1)<br>1151(H4HN2-PA)	0.57	
		4			1188.60	1188.45	$M+2H^+$	H3HN3NA1C-PA	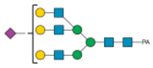	366(H1HN1)<br>657(H1HN1NA1)<br>1355(H1HN1C-PA)	0.38	
	pk.3-7	1	39.71	39.06-40.39	1188.17			data not available			0.96	x MS2
	pk.3-8	1	41.47	40.95-41.93	985.14	985.37	$M+2H^+$	H3HN1NA1C-PA	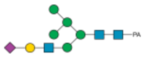	366(H1HN1)<br>657(H1HN1NA1)<br>1151(H4HN2-PA)<br>1314(H5HN2-PA)	3.41	
	pk.3-9	1	42.69	42.07-43.26	904.29	904.34	$M+2H^+$	H2HN1NA1C-PA	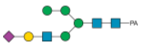	366(H1HN1)<br>657(H1HN1NA1)<br>1151(H4HN2-PA)	2.55	
		2			925.44	924.85	$M+2H^+$	H1HN2NA1C-PA	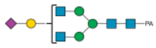	366(H1HN1)<br>657(H1HN1NA1)<br>1192(H3HN3-PA)	0.52	
	pk.3-10	1	44.99	44.23-45.84	671.13<br>1005.87	670.92<br>1005.88	$M+3H^+$<br>$M+2H^+$	H2HN2NA1C-PA	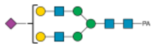	366(H1HN1)<br>657(H1HN1NA1)<br>1192(H3HN3-PA)	12.16	Standard a
	pk.3-11	1	47.90	47.03-48.57	1079.35	1078.91	$M+2H^+$	H2HN2F1NA1C-PA	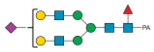	366(H1HN1)<br>657(H1HN1NA1)<br>1339(H3HN3F1-PA)	3.45	epimer<br>(pk.3-18-1)
	pk.3-12	1	50.11	48.92-50.73	896.15	896.34	$M+2H^+$	H1HN1F1NA1C-PA	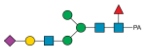	366(H1HN1)<br>657(H1HN1NA1)<br>973(H2HN2F1-PA)<br>1135(F1C-PA)	1.62	
		2			1006.30	1005.88	$M+2H^+$	H2HN2NA1C-PA	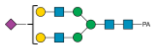	366(H1HN1)<br>657(H1HN1NA1)<br>1192(H3HN3-PA)	6.46	Standard c

Table S1 Continued.

Fr. No. (DEAE)	Peak. No. (ODS)	Full MS No.	Elusion time max (min)	Elusion time range (min)	Observed parent ion (*m/z* value)	Calculated (*m/z* value)	Estimated adduct	Estimated composition[a), c)]	Deduced glycan structure[b)]	Characteristic fragments[a)]	Relative amounts[d)]	Notes[f)]
		3			841.43<br>1261.84	841.32<br>1261.48	$M+3H^+$<br>$M+2H^+$	H3HN3F1NA1C-PA	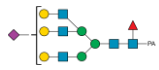	366(H1HN1NA1)<br>657(H1HN1NA1)<br>1339(H3HN3F1-PA)	1.88	
	pk.3-13	1	51.93	50.80-52.55	1005.74	1005.88	$M+2H^+$	H2HN2NA1C-PA	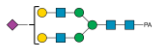	366(H1HN1)<br>657(H1HN1NA1)	1.67	Standard d
		2			1058.30	1058.40	$M+2H^+$	H3HN1F1NA1C-PA	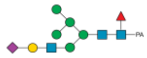	366(H1HN1)<br>1135(F1C-PA)<br>1460(H5HN2F1-PA)	0.66	
		3			1188.31	1188.45	$M+2H^+$	H3HN3NA1C-PA	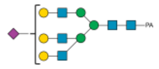	366(H1HN1)<br>657(H1HN1NA1)<br>1192(H3HN3-PA)	1.25	
		4			1261.72	1261.48	$M+2H^+$	H3HN3F1NA1C-PA	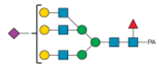	366(H1HN1)<br>657(H1HN1NA1)<br>1339(H3HN3F1-PA)	1.45	
	pk.3-14	1	53.92	52.97-55.28	977.53	977.37	$M+2H^+$	H2HN1F1NA1C-PA	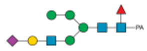	366(H1HN1)<br>657(H1HN1NA1)<br>1135(F1C-PA)<br>1298(H4HN2F1-PA)	1.57	
		2			1180.43	1180.45	$M+2H^+$	H2HN3F1NA1C-PA	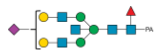	366(H1HN1)<br>657(H1HN1NA1)<br>1339(H3HN3F1-PA)	0.76	epimer<br>(pk.3-23-1)
		3			1326.51	1326.51	$M+2H^+$	H2HN3F3NA1C-PA	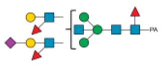	512(H1HN1F1)<br>803(H1HN1F1NA1)<br>1704(H1HN2F1C-PA)	0.80	Le^X<br>sLe^X
		4			1099.20	1099.42	$M+2H^+$	H1HN3F1NA1C-PA	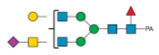	366(H1HN1)<br>407(HN2)<br>698(HN2NA1)<br>1339(H3HN3F1-PA)	0.54	sLacdiNAc
	pk.3-15	1	56.16	55.49-56.60	719.57<br>1079.39	719.61<br>1078.91	$M+3H^+$<br>$M+2H^+$	H2HN2F1NA1C-PA	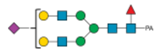	366(H1HN1)<br>657(H1HN1NA1)<br>973(H2HN2F1-PA)<br>1135(F1C-PA)	5.61	Standard e
	pk.3-16	1	57.23	56.74-57.91	836.15<br>1253.77	835.99<br>1253.48	$M+3H^+$<br>$M+2H^+$	H2HN3F2NA1C-PA	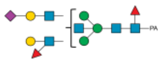	366(H1HN1)<br>512(H1HN1F1)<br>657(H1HN1NA1)<br>1339(H3HN3F1-PA)	5.23	Le^X
		2			1107.87	1107.42	$M+2H^+$	H2HN3NA1C-PA	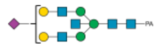	366(H1HN1)<br>657(H1HN1NA1)<br>1192(H3HN3-PA)	1.53	
		3			1326.37	1326.51	$M+2H^+$	H2HN3F3NA1C-PA	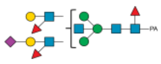	512(H1HN1F1)<br>657(H1HN1NA1)<br>803(H1HN1F1NA1)<br>1339(H3HN3F1-PA)	0.51	Le^X<br>sLe^X
		4			973.34	973.34	$M+2H^+$	H2HN2F1(SO3)1C-PA	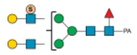	366(H1HN1)<br>1339(H3HN3F1-PA)<br>1581(H1HN1F1(SO3)1C-PA)	0.35	LacNAc(SO3)
	pk.3-17	1	60.16	59.47-60.66	997.68	997.88	$M+2H^+$	H1HN2F1NA1C-PA	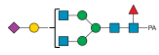	366(H1HN1)<br>657(H1HN1NA1)<br>973(H2HN2F1-PA)<br>1135(F1C-PA)<br>1339(H3HN3F1-PA)	2.24	
	pk.3-18	1	62.04	60.94-63.17	719.57<br>1079.22	719.61<br>1078.91	$M+3H^+$<br>$M+2H^+$	H2HN2F1NA1C-PA	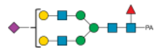	366(H1HN1)<br>657(H1HN1NA1)<br>1135(F1C-PA)<br>1339(H3HN3F1-PA)	21.43	
	pk.3-19	1	63.71	63.31-64.64	909.15<br>1362.90	909.01<br>1363.01	$M+3H^+$<br>$M+2H^+$	H3HN4F1NA1C-PA	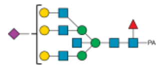	366(H1HN1)<br>657(H1HN1NA1)<br>1339(H3HN3F1-PA)	2.17	
		2			1099.45	1099.42	$M+2H^+$	H1HN3F1NA1C-PA	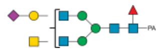	366(H1HN1)<br>407(HN2)<br>657(H1HN1NA1)<br>1339(H3HN3F1-PA)	1.29	LacdiNAc

Table S1 Continued.

Fr. No. (DEAE)	Peak. No. (ODS)	Full MS No.	Elusion time max (min)	Elusion time range (min)	Observed parent ion (*m/z* value)	Calculated (*m/z* value)	Estimated adduct	Estimated composition[a), c)]	Deduced glycan structure[b)]	Characteristic fragments[a)]	Relative amounts[d)]	Notes[f)]
	pk.3-20	1	65.73	64.78-66.81	1107.12	1107.42	M+2H+	H2HN3NA1C-PA	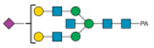	366(H1HN1)<br>657(H1HN1NA1)<br>1396(HN2C-PA)	1.86	
		2			1006.55			data not available			1.10	x MS2
	pk.3-21	1	67.76	67.02-68.41	841.59<br>1261.05	841.32<br>1261.48	M+3H+<br>M+2H+	H3HN3F1NA1C-PA	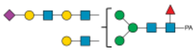	366(H1HN1)<br>657(H1HN1NA1)<br>731(H2HN2)<br>1022(H2HN2NA1)	2.03	sLacNAc repeat
	pk.3-22	1	68.97	68.48-69.46	1099.75	1099.42	M+2H+	H1HN3F1NA1C-PA	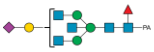	366(H1HN1)<br>657(H1HN1NA1)<br>1135(F1C-PA)<br>1339(H3HN3F1-PA)	1.69	
	pk.3-23	1	72.61	71.77-73.72	787.38<br>1180.44	787.30<br>1180.45	M+3H+<br>M+2H+	H2HN3F1NA1C-PA	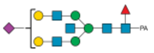	366(H1HN1)<br>657(H1HN1NA1)<br>1135(F1C-PA)<br>1339(H3HN3F1-PA)	10.56	Standard g
	pk.3-24	1	74.81	74.14-75.19	962.87			data not available			1.28	xMS2
	pk.3-25	1	75.66	75.26-76.24	1099.33	1099.42	M+2H+	H1HN3F1NA1C-PA	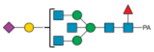	366(H1HN1)<br>657(H1HN1NA1)<br>1339(H3HN3F1-PA)<br>1542(H3HN4F1-PA)	1.35	
	pk.3-26	1	76.75	76.38-77.15	1084.67			data not available			0.81	xMS2
	pk.3-27	1	78.16	77.29-78.76	787.47<br>1180.68	787.30<br>1180.45	M+3H+<br>M+2H+	H2HN3F1NA1C-PA	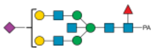	366(H1HN1)<br>657(H1HN1NA1)<br>1339(H3HN3F1-PA)<br>1542(H3HN4F1-PA)	5.68	(pk.1-36-1)
		2			1031.09	1030.72	M+3H+	H4HN5F1NA1C-PA	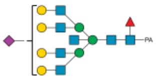	366(H1HN1)<br>657(H1HN1NA1)<br>1542(H3HN4F1-PA)	0.30	
	pk.3-28	1	79.28	78.90-79.87	733.31<br>1099.56	733.28<br>1099.42	M+3H+<br>M+2H+	H1HN3F1NA1C-PA	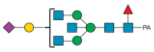	366(H1HN1)<br>657(H1HN1NA1)<br>1135(F1C-PA)<br>1339(H3HN3F1-PA)	2.20	
	pk.3-29	1	80.51	79.94-81.20	787.38<br>1180.64	787.30<br>1180.45	M+3H+<br>M+2H+	H2HN3F1NA1C-PA	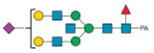	366(H1HN1)<br>657(H1HN1NA1)<br>1339(H3HN3F1-PA)<br>1542(H3HN4F1-PA)	4.70	
		2			909.14<br>1363.22	909.01<br>1363.01	M+3H+<br>M+2H+	H3HN4F1NA1C-PA	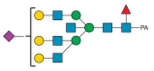	366(H1HN1)<br>657(H1HN1NA1)<br>1542(H3HN4F1-PA)	1.75	(pk.1-37-1)
	pk.3-30	1	81.38	81.21-81.40	1030.82	1030.72	M+3H+	H4HN5F1NA1C-PA	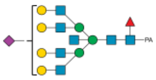	366(H1HN1)<br>657(H1HN1NA1)<br>1542(H3HN4F1-PA)	0.91	
	pk.3-31	1	81.98	81.41-82.74	908.92<br>1363.28	909.01<br>1363.01	M+3H+<br>M+2H+	H3HN4F1NA1C-PA	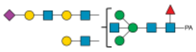	366(H1HN1)<br>657(H1HN1NA1)<br>731(H2HN2)<br>1022(H2HN2NA1)	3.11	sLacNAc repeat
	pk.3-32	1	83.65	83.09-84.63	976.97			data not available			1.76	xMS2
	pk.3-33	1	86.43	86.02-86.86	1030.67	1030.72	M+3H+	H4HN5F1NA1C-PA	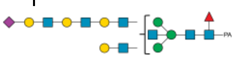	366(H1HN1)<br>657(H1HN1NA1)<br>731(H2HN2)<br>1096(H3HN3)<br>1339(H3HN3F1-PA)<br>1388(H3HN3NA1)	0.67	sLacNAc repeat
fr.4	pk.4-1	1	16.14	15.77-16.39	550.28	550.22	M+H+	H1NA1-PA	unknown		2.25	non-*N*-glycan

Table S1 Continued.

Fr. No. (DEAE)	Peak. No. (ODS)	Full MS No.	Elusion time max (min)	Elusion time range (min)	Observed parent ion (*m/z* value)	Calculated (*m/z* value)	Estimated adduct	Estimated composition[a), c)]	Deduced glycan structure[b)]	Characteristic fragments[a)]	Relative amounts[d)]	Notes[f)]
	pk.4-2	1	16.77	16.46-17.08	753.22	753.30	$M+H^+$	H1HN1NA1-PA	unknown		0.77	non-*N* -glycan
	pk.4-3	1	17.41	17.22-17.64	899.33	899.36	$M+H^+$	H1HN1F1NA1-PA	unknown		1.39	non-*N* -glycan
	pk.4-4	1	19.76	19.36-20.13	549.22	550.22	$M+H^+$	H1NA1-PA	unknown		0.49	non-*N* -glycan
		2			753.30	753.30	$M+H^+$	H1HN1NA1-PA	unknown		0.05	non-*N* -glycan
	pk.4-5	1	22.44	21.85-22.89	753.29	753.30	$M+H^+$	H1HN1NA1-PA	unknown		1.37	non-*N* -glycan
	pk.4-6	1	30.09	29.74-30.43	766.27			data not available			0.15	non-glycan
	pk.4-7	1	38.40	37.97-38.73	775.36			data not available			0.52	non-glycan
	pk.4-8	1	48.45	47.86-49.10	1084.70			data not available			1.72	x MS2
	pk.4-9	1	52.65	51.66-53.60	1370.86	1370.51	$M+2H^+$	H2HN2F3NA2C-PA	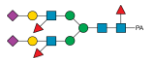	512(H1HN1F1)<br>657(H1HN1NA1)<br>803(H1HN1F1NA1)<br>1501(H1HN1F1C-PA)	3.00	sLe^X
	pk.4-10	1	55.04	53.74-55.67	938.48<br>1407.91	938.35<br>1407.02	$M+3H^+$<br>$M+2H^+$	H3HN3F1NA2C-PA	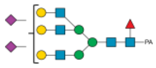	366(H1HN1)<br>657(H1HN1NA1)<br>1339(H3HN3F1-PA)	4.71	
	pk.4-11	1	56.47	55.74-57.26	889.79<br>1334.36	889.67<br>1333.99	$M+3H^+$<br>$M+2H^+$	H3HN3NA2C-PA	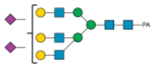	366(H1HN1)<br>657(H1HN1NA1)<br>1192(H3HN3-PA)	3.15	
	pk.4-12	1	57.87	57.40-58.44	1060.44			data not available			1.90	xMS2
	pk.4-13	1	59.19	58.58-60.65	1181.93	1181.77	$M+3H^+$	H5HN5F1NA2C-PA	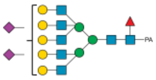	366(H1HN1)<br>657(H1HN1NA1)<br>1339(H3HN3F1-PA)	4.02	
	pk.4-14	1	61.67	61.00-62.59	981.81<br>1472.29	981.71<br>1472.05	$M+3H^+$<br>$M+2H^+$	H2HN3F3NA2C-PA	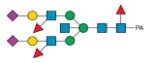	366(H1HN1)<br>657(H1HN1NA1)<br>803(H1HN1F1NA1)<br>1339(H3HN3F1-PA)	6.10	sLe^X
	pk.4-15	1	63.41	62.73-64.04	933.02<br>1398.90	933.02<br>1399.03	$M+3H^+$<br>$M+2H^+$	H2HN3F2NA2C-PA	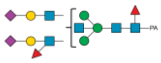	366(H1HN1)<br>657(H1HN1NA1)<br>803(H1HN1F1NA1)<br>1339(H3HN3F1-PA)	3.25	sLe^X
	pk.4-16	1	66.78	66.05-67.78	938.40<br>1407.33	938.35<br>1407.02	$M+3H^+$<br>$M+2H^+$	H3HN3F1NA2C-PA	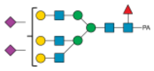	366(H1HN1)<br>657(H1HN1NA1)<br>1339(H3HN3F1-PA)	1.59	
		2			1059.89	1060.06	$M+3H^+$	H4HN4F1NA2C-PA	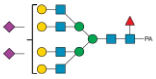	366(H1HN1)<br>657(H1HN1NA1)<br>1339(H3HN3F1-PA)	1.49	
	pk.4-17	1	69.42	68.81-70.96	1006.21	1006.04	$M+3H^+$	H3HN4F1NA2C-PA	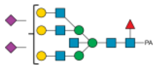	366(H1HN1)<br>446(F1HN1-PA)<br>657(H1HN1NA1)<br>1542(H3HN4F1-PA)	1.99	

Table S1 Continued.

Fr. No. (DEAE)	Peak. No. (ODS)	Full MS No.	Elusion time max (min)	Elusion time range (min)	Observed parent ion (*m/z* value)	Calculated (*m/z* value)	Estimated adduct	Estimated composition[a), c)]	Deduced glycan structure[b)]	Characteristic fragments[a)]	Relative amounts[d)]	Notes[f)]
		2			1151.99	1152.10	$M+3H^+$	H3HN4F4NA2C-PA	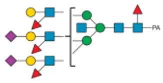	366(H1HN1)<br>657(H1HN1NA1)<br>803(H1HN1F1NA1)<br>1542(H3HN4F1-PA)	1.99	Le^x<br>sLe^x
		3			1103.42	1103.42	$M+3H^+$	H3HN4F3NA2C-PA	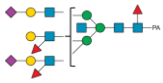	366(H1HN1)<br>657(H1HN1NA1)<br>803(H1HN1F1NA1)<br>1542(H3HN4F1-PA)	0.68	Le^x<br>sLe^x
		4			1127.73			data not available			1.18	x MS2
	pk.4-18	1	73.46	72.48-74.21	938.85<br>1407.28	938.35<br>1407.02	$M+3H^+$<br>$M+2H^+$	H3HN3F1NA2C-PA	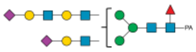	657(H1HN1NA)<br>731(H2HN2)<br>1022(H2HN2NA1)<br>1339(H3HN3F1-PA)	2.90	sLacNAc repeat
	pk.4-19	1	78.85	78.50-79.32	1060.22	1060.06	$M+3H^+$	H4HN4F1NA2C-PA	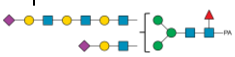	657(H1HN1NA)<br>731(H2HN2)<br>1022(H2HN2NA1)<br>1339(H3HN3F1-PA)	2.10	sLacNAc repeat
	pk.4-20	1	79.85	79.39-80.15	1128.17	1127.75	$M+3H^+$	H4HN5F1NA2C-PA	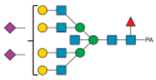	366(H1HN1)<br>657(H1HN1NA1)	2.02	
	pk.4-21	1	81.91	80.57-82.30	1128.34	1127.75	$M+3H^+$	H4HN5F1NA2C-PA	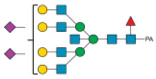	366(H1HN1)<br>657(H1HN1NA1)<br>1542(H3HN4F1-PA)	3.67	
		2			1073.62	1073.74	$M+3H^+$	H3HN5F1NA2C-PA	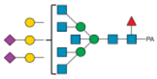	366(H1HN1)<br>657(H1HN1NA)<br>1542(H3HN4F1-PA)	1.36	
		3			1006.14	1006.04	$M+3H^+$	H3HN4F1NA2C-PA	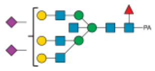	366(H1HN1)<br>657(H1HN1NA1)<br>1542(H3HN4F1-PA)	1.12	
	pk.4-22	1	82.63	82.44-83.27	1073.87	1073.74	$M+3H^+$	H3HN5F1NA2C-PA	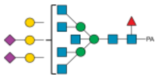	366(H1HN1)<br>657(H1HN1NA1)<br>1704(H1HN2F1C-PA)	0.88	
		2			1127.76	1127.75	$M+3H^+$	H4HN5F1NA2C-PA	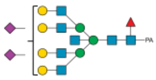	366(H1HN1)<br>657(H1HN1NA1)	0.65	
		3			1006.29	1006.04	$M+3H^+$	H3HN4F1NA2C-PA	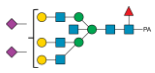	366(H1HN1)<br>657(H1HN1NA1)<br>1542(H3HN4F1-PA)	0.63	(pk.5-19-1)
	pk.4-23	1	84.03	83.41-84.51	1073.67	1073.74	$M+3H^+$	H3HN5F1NA2C-PA	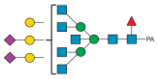	366(H1HN1)<br>657(H1HN1NA1)<br>1542(H3HN4F1-PA)	2.43	
	pk.4-24	1	85.44	84.86-86.10	1005.97	1006.04	$M+3H^+$	H3HN4F1NA2C-PA	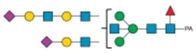	657(H1HN1NA)<br>731(H2HN2)<br>1022(H2HN2NA1)<br>1501(H1HN1F1C-PA)	2.37	sLacNAc repeat
fr.5	pk.5-1	1	18.00	17.82-18.31	665.19			data not available			0.50	xMS2
	pk.5-2	1	37.18	36.72-37.76	767.93<br>1151.74	767.95<br>1151.43	$M+3H^+$<br>$M+2H^+$	H2HN2NA2C-PA	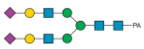	366(H1HN1)<br>657(H1HN1NA1)<br>1192(H3HN3-PA)	1.07	epimer<br>(pk.5-4-1)
	pk.5-3	1	43.52	43.08-43.85	889.64<br>1333.99	889.67<br>1333.99	$M+3H^+$<br>$M+2H^+$	H3HN3NA2C-PA	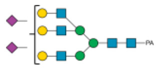	366(H1HN1)<br>657(H1HN1NA1)<br>1355(H1HN1C-PA)	0.81	
	pk.5-4	1	48.93	48.06-49.72	768.49<br>1151.50	767.95<br>1151.43	$M+3H^+$<br>$M+2H^+$	H2HN2NA2C-PA	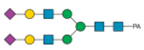	366(H1HN1)<br>657(H1HN1NA1)<br>1192(H3HN3-PA)	25.27	Standard A<br>(pk.6-8-1)

Table S1 Continued.

Fr. No. (DEAE)	Peak. No. (ODS)	Full MS No.	Elusion time max (min)	Elusion time range (min)	Observed parent ion (*m/z* value)	Calculated (*m/z* value)	Estimated adduct	Estimated composition[a), c)]	Deduced glycan structure[b)]	Characteristic fragments[a)]	Relative amounts[d)]	Notes[f)]
	pk.5-5	1	50.64	50.00-57.40	767.96<br>1151.75	767.95<br>1151.43	M+3H$^+$<br>M+2H$^+$	H2HN2NA2C-PA	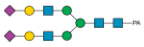	366(H1HN1)<br>657(H1HN1NA1)<br>1192(H3HN3-PA)	3.04	
	pk.5-6	1	53.15	52.42-53.87	816.71<br>1224.46	816.64<br>1224.46	M+3H$^+$<br>M+2H$^+$	H2HN2F1NA2C-PA	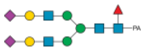	366(H1HN1)<br>657(H1HN1NA1)<br>973(H2HN2F1-PA)<br>1135(F1C-PA)<br>1339(H3HN3F1-PA)	2.30	epimer<br>(pk.5-12-1)
	pk.5-7	1	55.07	54.36-55.88	938.29<br>1406.93	938.35<br>1407.02	M+3H$^+$<br>M+2H$^+$	H3HN3F1NA2C-PA	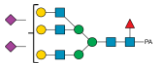	366(H1HN1)<br>657(H1HN1NA1)<br>1339(H3HN3F1-PA)	6.45	
	pk.5-8	1	56.62	56.02-57.40	889.87<br>1334.44	889.67<br>1333.99	M+3H$^+$<br>M+2H$^+$	H3HN3NA2C-PA	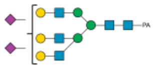	366(H1HN1)<br>657(H1HN1NA1)<br>1192(H3HN3-PA)	3.58	
		2			1151.20	1151.43	M+2H$^+$	H2HN2NA2C-PA	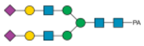	366(H1HN1)<br>657(H1HN1NA1)<br>1192(H3HN3-PA)	2.25	
	pk.5-9	1	59.01	58.09-60.10	816.80<br>1224.45	816.64<br>1224.46	M+3H$^+$<br>M+2H$^+$	H2HN2F1NA2C-PA	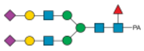	366(H1HN1)<br>657(H1HN1NA1)<br>973(H2HN2F1-PA)<br>1135(F1C-PA)<br>1339(H3HN3F1-PA)	9.37	Standard B
	pk.5-10	1	62.81	62.03-63.00	816.86<br>1224.41	816.64<br>1224.46	M+3H$^+$<br>M+2H$^+$	H2HN2F1NA2C-PA	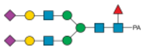	366(H1HN1)<br>657(H1HN1NA1)<br>973(H2HN2F1-PA)<br>1135(F1C-PA)<br>1339(H3HN3F1-PA)	2.73	
	pk.5-11	1	63.58	63.07-69.16	933.05<br>1399.33	933.02<br>1399.03	M+3H$^+$<br>M+2H$^+$	H2HN3F2NA2C-PA	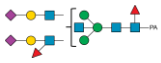	366(H1HN1)<br>657(H1HN1NA1)<br>803(H1HN1F1NA1)<br>1339(H3HN3F1-PA)	4.25	sLe^X
	pk.5-12	1	68.48	67.63-69.16	816.85<br>1224.40	816.64<br>1224.46	M+3H$^+$<br>M+2H$^+$	H2HN2F1NA2C-PA	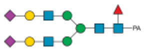	366(H1HN1)<br>657(H1HN1NA1)<br>973(H2HN2F1-PA)<br>1135(F1C-PA)<br>1339(H3HN3F1-PA)	14.55	(pk.6-10-1)
	pk.5-13	1	73.67	73.03-74.14	884.89<br>1326.25	884.33<br>1326.00	M+3H$^+$<br>M+2H$^+$	H2HN3F1NA2C-PA	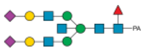	366(H1HN1)<br>657(H1HN1NA1)<br>1339(H3HN3F1-PA)	3.40	Standard D
	pk.5-14	1	74.77	74.27-75.24	938.68<br>1406.75	938.35<br>1407.02	M+3H$^+$<br>M+2H$^+$	H3HN3F1NA2C-PA	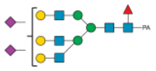	366(H1HN1)<br>657(H1HN1NA1)<br>973(H2HN2F1-PA)<br>1339(H3HN3F1-PA)	1.27	
	pk.5-15	1	76.66	76.07-77.25	884.75<br>1326.23	884.33<br>1326.00	M+3H$^+$<br>M+2H$^+$	H2HN3F1NA2C-PA	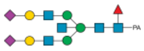	366(H1HN1)<br>657(H1HN1NA1)<br>1339(H3HN3F1-PA)	2.68	
	pk.5-16	1	77.59	77.32-77.87	938.41<br>1407.01	938.35<br>1407.02	M+3H$^+$<br>M+2H$^+$	H3HN3F1NA2C-PA	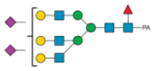	657(H1HN1NA1)<br>1339(H3HN3F1-PA)	0.64	
	pk.5-17	1	78.54	78.08-79.11	782.00<br>1172.84			data not available			1.10	(artifact)
	pk.5-18	1	81.56	80.98-82.09	884.24<br>1325.88	884.33<br>1326.00	M+3H$^+$<br>M+2H$^+$	H2HN3F1NA2C-PA	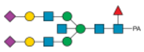	366(H1HN1)<br>657(H1HN1NA1)<br>1339(H3HN3F1-PA)	3.74	
	pk.5-19	1	82.65	82.23-83.20	1006.04<br>1508.98	1006.04<br>1508.56	M+3H$^+$<br>M+2H$^+$	H3HN4F1NA2C-PA	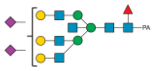	366(H1HN1)<br>657(H1HN1NA1)<br>1542(H3HN4F1-PA)	1.53	(pk.4-22-3)
fr.6	pk.6-1	1	11.12	10.84-11.67	1102.01			data not available			0.18	xMS2
	pk.6-2	1	13.03	12.42-13.32	859.10	859.29	M+2H$^+$	H4(HPO3)1C-PA	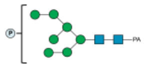	243(H1(HPO3)1)<br>325(H2)<br>405(H2(HPO3)1)<br>1151(H4HN2-PA)	0.42	HPO3

Table S1 Continued.

Fr. No. (DEAE)	Peak. No. (ODS)	Full MS No.	Elusion time max (min)	Elusion time range (min)	Observed parent ion (*m/z* value)	Calculated (*m/z* value)	Estimated adduct	Estimated composition[a), c)]	Deduced glycan structure[b)]	Characteristic fragments[a)]	Relative amounts[d)]	Notes[f)]
	pk.6-3	1	13.98	13.66-14.69	1102.35			data not available			2.05	(artifact)
	pk.6-4	1	15.77	15.58-16.33	1021.34			data not available			0.59	xMS2
	pk.6-5	1	18.24	17.64-18.67	940.17	940.32	$M+2H^+$	H5(HPO3)1C-PA	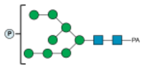	405(H2(HPO3)1)<br>1151(H4HN2-PA)	0.55	HPO3
	pk.6-6	1	19.05	18.81-19.49	858.98	859.29	$M+2H^+$	H4(HPO3)1C-PA	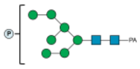	243(H1(HPO3)1)<br>405(H2(HPO3)1)<br>1151(H4HN2-PA)	0.42	HPO3
	pk.6-7	1	47.29	46.53-47.84	1285.61			data not available			0.90	xMS2
	pk.6-8	1	48.77	48.18-49.69	768.15<br>1151.89	767.95<br>1151.43	$M+3H^+$<br>$M+2H^+$	H2HN2NA2C-PA	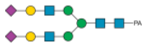	366(H1HN1)<br>657(H1HN1NA1)<br>1192(H3HN3-PA)	3.47	(pk.5-4-1)
	pk.6-9	1	61.25	60.47-62.11	1118.65	1118.89	$M+2H^+$	H2HN2F1NA1(SO3)1C-PA	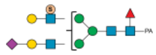	366(H1HN1)<br>657(H1HN1NA1)<br>1339(H3HN3F1-PA)<br>1581(H1HN1F1(SO3)1C-PA)	1.30	LacNAc(SO3)
	pk.6-10	1	68.09	67.40-68.84	817.18<br>1224.39	816.64<br>1224.46	$M+3H^+$<br>$M+2H^+$	H2HN2F1NA2C-PA	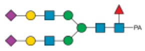	366(H1HN1)<br>657(H1HN1NA1)<br>973(H2HN2F1-PA)<br>1135(F1C-PA)<br>1339(H3HN3F1-PA)	1.33	(pk.5-12-1)
fr.7	pk.7-1	1	14.47	14.00-15.03	777.91	778.26	$M+2H^+$	H3(HPO3)1C-PA	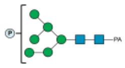	243(H1(HPO3)1)<br>405(H2(HPO3)1)<br>1151(H4HN2-PA)	2.11	HPO3<br>(pk.8-1-1)
	pk.7-2	1	22.09	21.69-22.37	778.03	778.26	$M+2H^+$	H3(HPO3)1C-PA	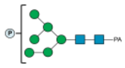	243(H1(HPO3)1)<br>405(H2(HPO3)1)<br>1151(H4HN2-PA)	0.52	HPO3
	pk.7-3	1	55.63	54.98-56.42	1119.33	1118.89	$M+2H^+$	H2HN2F1NA1(SO3)1C-PA	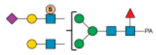	366(H1HN1)<br>657(H1HN1NA1)<br>1339(H3HN3F1-PA)<br>1581(H1HN1F1(SO3)1C-PA)	1.18	sLacNAc(SO3)<br>(α2,6)
	pk.7-4	1	57.47	56.62-58.06	1181.49	1181.44	$M+3H^+$	H3HN3F4NA3C-PA	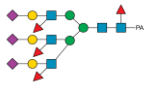	366(H1HN1)<br>657(H1HN1NA1)<br>803(H1HN1F1NA1)<br>973(H2HN2F1-PA)<br>1339(H3HN3F1-PA)	1.48	sLe^X
	pk.7-5	1	58.83	58.13-59.57	1119.20	1118.89	$M+2H^+$	H2HN2F1NA1(SO3)1C-PA	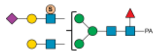	366(H1HN1)<br>657(H1HN1NA1)<br>1339(H3HN3F1-PA)<br>1581(H1HN1F1(SO3)1C-PA)	2.25	sLacNAc(SO3)<br>(α2,3)
	pk.7-6	1	70.43	69.18-71.04	1248.95	1249.13	$M+3H^+$	H3HN4F4NA3C-PA	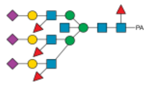	366(H1HN1)<br>657(H1HN1NA1)<br>803(H1HN1F1NA1)<br>1339(H3HN3F1-PA)	2.33	sLe^X
	pk.7-7	1	71.84	71.23-72.35	1279.46	1278.80	$M+3H^+$	H5HN5F1NA3C-PA	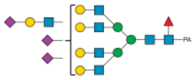	657(H1HN1NA1)<br>1022(H2HN2NA1)<br>1339(H3HN3F1-PA)	0.45	sLacNAc repeat
	pk.7-8	1	74.91	74.06-75.70	1400.89			data not available			1.51	xMS2
fr.8	pk.8-1	1	14.40	13.83-14.87	778.10	778.26	$M+2H^+$	H3(HPO3)C-PA	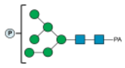	243(H1(HPO3)1)<br>405(H2(HPO3)1)<br>1151(H4HN2-PA)	0.62	HPO3<br>(pk.7-1-1)
	pk.8-2	1	20.09	19.85-20.61				data not available			0.21	xMS

Table S1 Continued.

Fr. No. (DEAE)	Peak. No. (ODS)	Full MS No.	Elusion time max (min)	Elusion time range (min)	Observed parent ion (*m/z* value)	Calculated (*m/z* value)	Estimated adduct	Estimated composition[a), c)]	Deduced glycan structure[b)]	Characteristic fragments[a)]	Relative amounts[d)]	Notes[f)]
	pk.8-3	1	46.59	46.13-47.03	986.72<br>1479.20	986.70<br>1479.54	$M+3H^{+}$<br>$M+2H^{+}$	H3HN3NA3C-PA	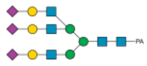	366(H1HN1)<br>657(H1HN1NA1)<br>1192(H3HN3-PA)	1.09	
	pk.8-4	1	50.04	49.17-51.60	1108.94			data not available			1.33	x MS2
		2			1035.50			data not available			1.36	x MS2
	pk.8-5	1	52.51	51.87-53.46	1108.48	1108.41	$M+3H^{+}$	H4HN4NA3C-PA	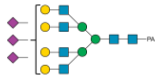	366(H1HN1)<br>657(H1HN1NA1)<br>1192(H3HN3-PA)	1.78	
	pk.8-6	1	54.27	53.53-55.05	1108.44	1108.41	$M+3H^{+}$	H4HN4NA3C-PA	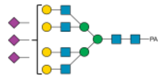	366(H1HN1)<br>657(H1HN1NA1)<br>1192(H3HN3-PA)	2.04	
		2			1200.16	1200.45	$M+3H^{+}$	H3HN4F3NA3C-PA	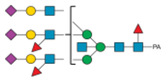	366(H1HN1)<br>446(HN1F1-PA)<br>657(H1HN1NA1)<br>803(H1HN1F1NA1)	0.38	sLe^{X}
	pk.8-7	1	55.38	55.19-56.02	1035.90			data not available			0.44	x MS2
		2			1108.35			data not available			1.02	x MS2
	pk.8-8	1	57.11	56.15-57.61	1035.48<br>1552.65	1035.38<br>1552.57	$M+3H^{+}$<br>$M+2H^{+}$	H3HN3F1NA3C-PA	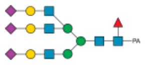	366(H1HN1)<br>657(H1HN1NA1)<br>1339(H3HN3F1-PA)	6.74	
	pk.8-9	1	58.10	57.68-58.78	986.72<br>1479.92	986.70<br>1479.54	$M+3H^{+}$<br>$M+2H^{+}$	H3HN3NA3C-PA	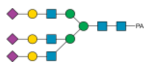	366(H1HN1)<br>657(H1HN1NA1)<br>1192(H3HN3-PA)	3.80	
	pk.8-10	1	60.66	59.82-61.34	987.16<br>1479.24	986.70<br>1479.54	$M+3H^{+}$<br>$M+2H^{+}$	H3HN3NA3C-PA	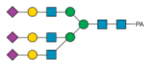	366(H1HN1)<br>657(H1HN1NA1)<br>1339(H3HN3F1-PA)	1.18	
		2			1278.77	1278.80	$M+3H^{+}$	H5HN5F1NA3C-PA	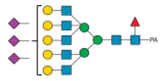	366(H1HN1)<br>657(H1HN1NA1)<br>1339(H3HN3F1-PA)	0.55	
		3			1157.04			data not available			0.58	x MS2
	pk.8-11	1	62.21	61.48-62.86	986.63	986.70	$M+3H^{+}$	H3HN3NA3C-PA	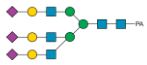	366(H1HN1)<br>657(H1HN1NA1)<br>1192(H3HN3-PA)	1.13	
		2			1157.57	1157.09	$M+3H^{+}$	H4HN4F1NA3C-PA	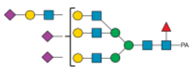	366(H1HN1)<br>657(H1HN1NA1)<br>731(H2HN2)<br>1022(H2HN2NA1)<br>1339(H3HN3F1-PA)	0.75	sLacNAc repeat
	pk.8-12	1	63.22	62.99-63.32	1157.40	1157.09	$M+3H^{+}$	H4HN4F1NA3C-PA	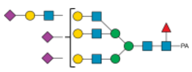	366(H1HN1)<br>657(H1HN1NA1)<br>731(H2HN2)<br>1022(H2HN2NA1)<br>1339(H3HN3F1-PA)	0.37	sLacNAc repeat
	pk.8-13	1	64.49	63.69-65.28	1157.49	1157.09	$M+3H^{+}$	H4HN4F1NA3C-PA	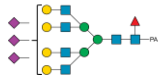	366(H1HN1)<br>657(H1HN1NA1)<br>1339(H3HN3F1-PA)	2.08	
	pk.8-14	1	66.34	65.77-67.01	1157.60	1157.09	$M+3H^{+}$	H4HN4F1NA3C-PA	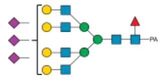	366(H1HN1)<br>657(H1HN1NA1)<br>1339(H3HN3F1-PA)	3.12	

Table S1 Continued.

Fr. No. (DEAE)	Peak. No. (ODS)	Full MS No.	Elusion time max (min)	Elusion time range (min)	Observed parent ion (*m/z* value)	Calculated (*m/z* value)	Estimated adduct	Estimated composition[a), c)]	Deduced glycan structure[b)]	Characteristic fragments[a)]	Relative amounts[d)]	Notes[f)]
	pk.8-15	1	67.40	67.04-67.79	1157.10	1157.09	$M+3H^+$	H4HN4F1NA3C-PA	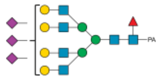	366(H1HN1)<br>657(H1HN1NA1)<br>1339(H3HN3F1-PA)	0.69	
		2			1103.57	1103.08	$M+3H^+$	H3HN4F1NA3C-PA	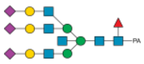	366(H1HN1)<br>657(H1HN1NA1)<br>1339(H3HN3F1-PA)	0.66	
	pk.8-16	1	68.49	67.84-69.57	1157.34	1157.09	$M+3H^+$	H4HN4F1NA3C-PA	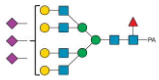	366(H1HN1)<br>657(H1HN1NA1)<br>1339(H3HN3F1-PA)	1.83	
		2			1035.45	1035.38	$M+3H^+$	H3HN3F1NA3C-PA	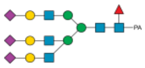	366(H1HN1)<br>657(H1HN1NA1)<br>973(H2HN2F1-PA)<br>1339(H3HN3F1-PA)	1.68	
	pk.8-17	1	70.32	69.71-70.89	1249.35			data not available			1.79	xMS2
	pk.8-18	1	72.10	71.02-72.75	1200.33	1200.45	$M+3H^+$	H3HN4F3NA3C-PA	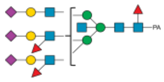	366(H1HN1)<br>657(H1HN1NA1)<br>803(H1HN1F1NA1)<br>1339(H3HN3F1-PA)	2.06	sLe^X
		2			1035.47<br>1552.27	1035.38<br>1552.57	$M+3H^+$<br>$M+2H^+$	H3HN3F1NA3C-PA	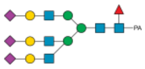	366(H1HN1)<br>657(H1HN1NA1)<br>973(H2HN2F1-PA)<br>1339(H3HN3F1-PA)	1.10	
	pk.8-19	1	73.30	72.89-74.20	1224.99	1224.79	$M+3H^+$	H4HN5F1NA3C-PA	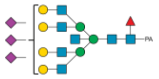	366(H1HN1)<br>657(H1HN1NA1)<br>1542(H3HN4F1-PA)	1.68	
	pk.8-20	1	76.83	76.21-77.18	1035.73<br>1552.83	1035.38<br>1552.57	$M+3H^+$<br>$M+2H^+$	H3HN3F1NA3C-PA	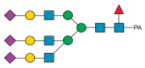	366(H1HN1)<br>657(H1HN1NA1)<br>1339(H3HN3F1-PA)	1.55	
	pk.8-21	1	77.84	77.32-78.42	1224.81			data not available			1.72	x MS2
	pk.8-22	1	78.90	78.56-79.39	1035.48	1035.38	$M+3H^+$	H3HN3F1NA3C-PA	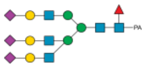	366(H1HN1)<br>657(H1HN1NA1)<br>1339(H3HN3F1-PA)<br>1542(H3HN4F1-PA)	1.03	
	pk.8-23	1	79.86	79.46-80.64	1224.70	1224.79	$M+3H^+$	H4HN5F1NA3C-PA	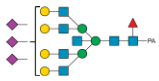	366(H1HN1)<br>657(H1HN1NA1)<br>1339(H3HN3F1-PA)<br>1542(H3HN4F1-PA)	2.00	
	pk.8-24	1	82.01	81.54-82.50	1103.11	1103.08	$M+3H^+$	H3HN4F1NA3C-PA	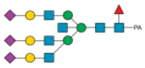	366(H1HN1)<br>657(H1HN1NA1)<br>1542(H3HN4F1-PA)	0.57	
		2			1225.06			data not available			0.51	x MS2
	pk.8-25	1	83.71	83.06-84.23	1224.68	1224.79	$M+3H^+$	H4HN5F1NA3C-PA	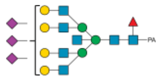	366(H1HN1)<br>657(H1HN1NA1)<br>1339(H3HN3F1-PA)	1.62	
		2			1103.48	1103.08	$M+3H^+$	H3HN4F1NA3C-PA	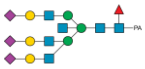	366(H1HN1)<br>657(H1HN1NA1)<br>1542(H3HN4F1-PA)	0.67	
fr.9	pk.9-1	1	48.42	47.15-49.28				data not available			1.62	xMS
	pk.9-2	1	50.68	49.55-51.41	965.11<br>1447.45			data not available			1.64	x MS2

Table S1 Continued.

Fr. No. (DEAE)	Peak. No. (ODS)	Full MS No.	Elusion time max (min)	Elusion time range (min)	Observed parent ion (*m/z* value)	Calculated (*m/z* value)	Estimated adduct	Estimated composition[a), c)]	Deduced glycan structure[b)]	Characteristic fragments[a)]	Relative amounts[d)]	Notes[f)]
	pk.9-3	1	52.06	51.54-53.53	1468.16				data not available		1.96	x MS2
	pk.9-4	1	58.44	57.79-59.51	1054.54				data not available		1.16	x MS2
	pk.9-5	1	60.95	59.64-61.84	965.40<br>1447.33				data not available		1.70	x MS2
	pk.9-6	1	70.86	70.21-73.30	1376.44	1375.84	M+3H+	H5HN5F1NA4C-PA	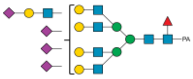	366(H1HN1)<br>657(H1HN1NA1)<br>731(H2HN2)<br>1022(H2HN2NA1)<br>1339(H3HN3F1-PA)	1.23	sLacNAc repeat
		2			914.25				data not available		1.67	x MS2
	pk.9-7	1	74.32	73.64-74.88	1497.58	1497.55	M+3H+	H6HN6F1NA4C-PA	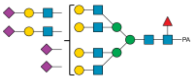	366(H1HN1)<br>657(H1HN1NA1)<br>731(H2HN2)<br>973(H2HN2F1-PA)<br>1022(H2HN2NA1)<br>1339(H3HN3F1-PA)	0.94	sLacNAc repeat
		2			914.25				data not available		0.23	x MS2
		3			1123.45				data not available		0.25	x MS2
	pk.9-8	1	75.43	75.37-75.69	1497.68	1497.55	M+3H+	H6HN6F1NA4C-PA	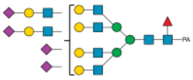	366(H1HN1)<br>657(H1HN1NA1)<br>731(H2HN2)<br>1022(H2HN2NA1)<br>1339(H3HN3F1-PA)	0.99	sLacNAc repeat
		2			1123.60				data not available		0.26	x MS2
fr.10	pk.10-1	1	10.29	10.02-11.26	1000.52				data not available		2.52	(artifact)
	pk.10-2	1	13.07	12.63-13.45	1000.84				data not available		0.74	(artifact)
	pk.10-3	1	46.79	46.12-47.49	699.20				data not available		1.06	non-glycan
	pk.10-4	1	55.24	54.50-55.66	1205.76	1205.44	M+3H+	H4HN4NA4C-PA	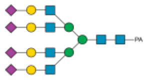	366(H1HN1)<br>657(H1HN1NA1)<br>1396(HN2C-PA)	1.77	
	pk.10-5	1	56.30	55.73-57.24	1205.52				data not available		2.13	x MS2
	pk.10-6	1	61.83	61.08-62.39	1264.23	1264.44	M+2H+	H2HN2F1NA2(SO3)1-PA	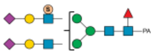	657(H1HN1NA1)<br>737(H1HN1NA1(SO3)1)<br>1135(F1C-PA)<br>1339(H3HN3F1-PA)<br>1581(H1HN1F1(SO3)1C-PA)	3.22	sLacNAc(SO3) (α2,3)
	pk.10-7	1	66.42	65.55-66.99	1254.19	1254.13	M+3H+	H4HN4F1NA4C-PA	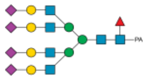	366(H1HN1)<br>657(H1HN1NA1)<br>1339(H3HN3F1-PA)	2.51	
	pk.10-8	1	67.57	67.06-68.36	1254.61	1254.13	M+3H+	H4HN4F1NA4C-PA	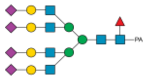	366(H1HN1)<br>657(H1HN1NA1)<br>1339(H3HN3F1-PA)	2.74	

Table S1 Continued.

Fr. No. (DEAE)	Peak. No. (ODS)	Full MS No.	Elusion time max (min)	Elusion time range (min)	Observed parent ion (*m/z* value)	Calculated (*m/z* value)	Estimated adduct	Estimated composition[a), c)]	Deduced glycan structure[b)]	Characteristic fragments[a)]	Relative amounts[d)]		Notes[f)]
	pk.10-9	1	74.79	74.12-75.57	1321.68			data not available			1.27		x MS2
	pk.10-10	1	81.24	80.58-82.02	1322.21			data not available			0.96		x MS2
fr.11	pk.11-1	1	37.11	36.86-37.54				data not available			0.31		non-glycan
	pk.11-2	1	39.16	38.92-39.74				data not available			0.42		non-glycan
	pk.11-3	1	51.20	50.52-52.03	1062.06	1062.04	$M+3H^+$	H3HN3F1NA3(SO3)1C-PA	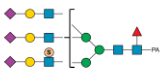	366(H1HN1)<br>657(H1HN1NA1)<br>737(H1HN1NA1(SO3)1)<br>1339(H3HN3F1-PA)	1.77		sLacNAc(SO3)<br>(α2,3)
	pk.11-4	1	66.82	65.82-68.02	1351.58	1351.16	$M+3H^+$	H4HN4F1NA5C-PA	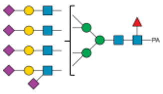	657(H1HN1NA1)	1.20		
