# Supplementary material for: Toward robust N-glycomics of various tissue samples that may contain glycans with unknown or unexpected structures
Source: Sci Rep. 2021 Mar 18;11:6334. doi: 10.1038/s41598-021-84668-x (PMC7973440; doi:10.1038/s41598-021-84668-x)
Supplement: Supplementary file 4 — Supplementary Information 4. [file 41598_2021_84668_MOESM4_ESM.pdf]

Table S2. MS and MS/MS data for PA-*N*-glycans from chicken colon after two-step alkylamidation

[a] H, hexose; HN, *N*-acetylhexosamine; F, fucose (or deoxyhexose); NA, *N*-acetylneuraminic acid; NG, *N*-glycolylneuraminic acid; SO3, sulfate group; HPO3, phosphate group; C, trimannosyl core; PA, 2-aminopyridine.

[b] Sialyl linkage : ◇: α2,3; ◆ : α2,6

[c] Most of the detectable fluorescence peaks eluted in 10–87 min were numbered, but not all peaks were derived from PA-*N*-glycans. The compositions of some peaks could not be determined by MS and MS/MS analysis due to insufficent signals; these cases are indicated as "data not available".

[d] Individual peaks detected by fluorescence sometimes included more than two kinds of PA-glycans with different mass values. In such cases, the proportions were estimated using the ratios of integrated ion intensities for each *m/z* value detected at the corresponding elution time.

[e] Amounts of glycans relative to the most abundant glycan (pk. 5-16-1), for which the amount was defined as 100.

[f] xMS2, MS/MS data not available.

Fr. No. (DEAE)	Peak. No. (ODS)	Full MS No.	Elution time max (min)	Elution time range (min)	Observed parent ion (m/z value)	Calculated (m/z value)	Estimated adduct	Estimated composition[a), b), c)]		Characteristic fragments[a)]	Area[d)]	Relative amounts[e)]	Notes[f)]
fr.3	pk.3-1	1	15.50	15.07-15.83	879.48	879.80	$M+2H^+$	H3HN1(HPO3)1C-PA			314621	5.03	
		2			940.29	940.32	$M+2H^+$	H5(HPO3)1C-PA			146854	2.35	
	pk.3-2	1	16.25	15.90-16.46	1041.53	1041.86	$M+2H^+$	H5HN1(HPO3)1C-PA			109757	1.76	
		2			859.07	859.29	$M+2H^+$	H4(HPO3)1C-PA			66402	1.06	
		3			940.70			data not available			39077	0.63	
	pk.3-3	1	16.81	16.53-17.50	960.51	960.83	$M+2H^+$	H4HN1(HPO3)1C-PA			260219	4.16	
	pk.3-4	1	28.70	28.40-29.17	797.07<br>1194.79	796.98<br>1194.96	$M+3H^+$<br>$M+2H^+$	H3HN3NA1C-PA	◇1		546297	8.74	
	pk.3-5	1	30.25	29.93-30.56	992.18	991.88	$M+2H^+$	H3HN1NA1C-PA	◇1		1265794	20.25	
	pk.3-6	1	31.14	30.76-31.93	910.76	910.86	$M+2H^+$	H2HN1NA1C-PA	◇1		997406	15.96	
	pk.3-7	1	31.69	31.46-32.01	1012.40	1012.40	$M+2H^+$	H2HN2NA1C-PA	◇1		629012	10.06	
	pk.3-8	1	32.61	32.08-32.85	845.72<br>1267.67	845.66<br>1267.99	$M+3H^+$<br>$M+2H^+$	H3HN3F1NA1C-PA	◇1		755023	12.08	
		2			1085.19	1085.42	$M+2H^+$	H2HN2F1NA1C-PA	◇1		610572	9.77	
		3			888.92<br>1332.42	889.02<br>1333.02	$M+3H^+$<br>$M+2H^+$	H2HN3F3NA1C-PA	◇1	816(H1HN1F1NA1)	305246	4.88	sLe^X
	pk.3-9	1	33.12	32.92-33.54	845.62<br>1268.43	845.66<br>1267.99	$M+3H^+$<br>$M+2H^+$	H3HN3F1NA1C-PA	◇1		669034	10.70	
		2			973.11	973.34	$M+2H^+$	H2HN2F1(SO3)1C-PA			190574	3.05	
		3			1020.21			data not available			180449	2.89	
		4			1236.43			data not available			168335	2.69	
	pk.3-10	1	34.28	33.68-34.65	675.22<br>1012.66	675.27<br>1012.40	$M+3H^+$<br>$M+2H^+$	H2HN2NA1C-PA	◇1		1906515	30.50	
		2			889.46<br>1333.35	889.02<br>1333.02	$M+3H^+$<br>$M+2H^+$	H2HN3F3NA1C-PA	◇1	816(H1HN1F1NA1)	466083	7.46	sLe^X
	pk.3-11	1	35.14	34.72-35.56	1085.29	1085.42	$M+2H^+$	H2HN2F1NA1C-PA	◇1		666061	10.65	
		2			967.34	967.37	$M+3H^+$	H4HN4F1NA1C-PA	◇1		195424	3.13	
	pk.3-12	1	35.97	35.63-36.32	983.47	983.89	$M+2H^+$	H2HN1F1NA1C-PA	◇1		526800	8.43	
		2			902.39	902.86	$M+2H^+$	H1HN1F1NA1C-PA	◇1		199753	3.20	
		3			1186.64	1186.96	$M+2H^+$	H2HN3F1NA1C-PA	◇1		122341	1.96	
	pk.3-13	1	36.73	36.39-37.08	724.03<br>1085.38	723.95<br>1085.42	$M+3H^+$<br>$M+2H^+$	H2HN2F1NA1C-PA	◇1		1346166	21.53	
	pk.3-14	1	37.44	37.15-37.64	1093.31	1093.42	$M+2H^+$	H2HN2F1NG1C-PA	◇1		273395	4.37	
		2			1105.88	1105.94	$M+2H^+$	H1HN3F1NA1C-PA	◇1	407(HN2)	157940	2.53	LacdiNAc
		3			1186.63	1186.96	$M+2H^+$	H2HN3F1NA1C-PA	◇1		88474	1.42	
		4			1032.56	1032.91	$M+2H^+$	H1HN3NA1C-PA	◇1		119408	1.91	
		5			913.81	913.36	$M+3H^+$	H3HN4F1NA1C-PA	◇1		40102	0.64	
	pk.3-15	1	38.90	38.13-39.58	724.33<br>1085.15	723.95<br>1085.42	$M+3H^+$<br>$M+2H^+$	H2HN2F1NA1C-PA	◇1		5957936	95.31	
		2			1003.96	1004.40	$M+2H^+$	H1HN2F1NA1C-PA	◇1		218829	3.50	
		3			845.38<br>1267.97	845.66<br>1267.99	$M+3H^+$<br>$M+2H^+$	H3HN3F1NA1C-PA	◇1	731(H2HN2)<br>1035(H2HN2NA1)	345103	5.52	
	pk.3-16	1	40.26	39.93-40.90	1026.79	1026.41	$M+2H^+$	H2HN2NA1C-PA	◆1		451279	7.22	
		2			1209.23	1208.98	$M+2H^+$	H3HN3NA1C-PA	◆1		257642	4.12	
		3			1105.68	1105.94	$M+2H^+$	H1HN3F1NA1C-PA	◇1	407(HN2)	138712	2.22	LacdiNAc
		4			1267.73	1267.99	$M+2H^+$	H3HN3F1NA1C-PA	◇1		116705	1.87	
		5			1085.42	1085.42	$M+2H^+$	H2HN2F1NA1C-PA	◇1		89570	1.43	
	pk.3-17	1	41.54	41.04-41.81	1113.54	1113.94	$M+2H^+$	H2HN3NA1C-PA	◇1		496040	7.94	
		2			967.26	967.37	$M+3H^+$	H4HN4F1NA1C-PA	◇1	731(H2HN2)<br>1035(H2HN2NA1)	122717	1.96	sLacNAc repeat
	pk.3-18	1	43.00	42.57-43.13	1005.75	1005.90	$M+2H^+$	H3HN1NA1C-PA	◆1		458140	7.33	
		2			981.05	981.05	$M+3H^+$	H3HN5F1NA1C-PA	◇1		76857	1.23	
		3			1035.11	1035.07	$M+3H^+$	H4HN5F1NA1C-PA	◇1		70210	1.12	
		4			1107.30	1107.44	$M+2H^+$	H3HN2NA1C-PA	◆1		26104	0.42	
	pk.3-19	1	43.98	43.19-45.14	792.02<br>1187.23	791.65<br>1186.96	$M+3H^+$<br>$M+2H^+$	H2HN3F1NA1C-PA	◇1		2035770	32.57	
		2			1105.75	1105.94	$M+2H^+$	H1HN3F1NA1C-PA	◇1		485166	7.76	
		3			1035.01	1035.07	$M+3H^+$	H4HN5F1NA1C-PA	◇1		269333	4.31	
		4			1026.41	1026.41	$M+2H^+$	H2HN2NA1C-PA	◆1		282117	4.51	
		5			981.47	981.05	$M+3H^+$	H3HN5F1NA1C-PA	◇1		153397	2.45	

Table S2 Continued.

Fr. No. (DEAE)	Peak. No. (ODS)	Full MS No.	Elusion time max (min)	Elusion time range (min)	Observed parent ion (m/z value)	Calculated (m/z value)	Estimated adduct	Estimated composition[a), b), c)]		Characteristic fragments[a)]	Area[d)]	Relative amounts[e)]	Notes[f)]
	pk.3-20	1	45.62	45.28-46.25	854.95<br>1282.01	855.01<br>1282.01	M+3H+<br>M+2H+	H3HN3F1NA1C-PA	◆1		464281	7.43	
		2			1106.15	1105.94	M+2H+	H1HN3F1NA1C-PA	◇1		303924	4.86	
		3			1187.30	1186.96	M+2H+	H2HN3F1NA1C-PA	◇1		117941	1.89	
		4			1099.80	1099.44	M+2H+	H2HN2F1NA1C-PA	◆1		192934	3.09	
	pk.3-21	1	46.77	46.32-47.22	684.75<br>1026.73	684.61<br>1026.41	M+3H+<br>M+2H+	H2HN2NA1C-PA	◆1		4088770	65.41	
		2			1187.28	1186.96	M+2H+	H2HN3F1NA1C-PA	◇1		743617	11.90	
	pk.3-22	1	47.55	47.36-47.71	1172.75	1172.47	M+2H+	H2HN2F2NA1C-PA	◆1	512(H1HN1F1)	175012	2.80	Le^{X}
		2			1026.84	1026.41	M+2H+	H2HN2NA1C-PA	◆1		161207	2.58	
	pk.3-23	1	48.55	47.99-48.68	800.75<br>1200.45	800.99<br>1200.98	M+3H+<br>M+2H+	H2HN3F1NA1C-PA	◆1		288518	4.62	
		2			952.47<br>1428.01	952.38<br>1428.06	M+3H+<br>M+2H+	H3HN3F3NA1C-PA	◆1	512(H1HN1F1)	179111	2.87	Le^{X}
		3			1099.49	1099.44	M+2H+	H2HN2F1NA1C-PA	◆1		102428	1.64	
		4			1046.90	1046.92	M+2H+	H1HN3NA1C-PA	◆1	407(HN2)	76492	1.22	LacdiNAc
		5			1035.38	1035.07	M+3H+	H4HN5F1NA1C-PA	◇1	1096(H3HN3)<br>1401(H3HN3NA1)	205696	3.29	sLacNAc repeat3
	pk.3-24	1	48.96	48.75-49.38	806.06<br>1208.85	806.32<br>1208.98	M+3H+<br>M+2H+	H3HN3NA1C-PA	◆1		566365	9.06	
		2			860.14<br>1288.86	859.34<br>1288.50	M+3H+<br>M+2H+	H2HN4F1NA1C-PA	◇1		172767	2.76	
		3			913.66<br>1370.46	913.36<br>1369.53	M+3H+<br>M+2H+	H3HN4F1NA1C-PA	◇1		277331	4.44	
	pk.3-25	1	49.69	49.51-49.86	916.79	916.87	M+2H+	H1HN1F1NA1C-PA	◆1		314169	5.03	
		2			1369.37	1369.53	M+2H+	H3HN4F1NA1C-PA	◇1	731(H2HN2)<br>1035(H2HN2NA1)	136479	2.18	sLacNAc repeat
	pk.3-26	1	50.49	49.93-50.97	733.30<br>1099.37	733.30<br>1099.44	M+3H+<br>M+2H+	H2HN2F1NA1C-PA	◆1		1830827	29.29	
		2			1201.22	1200.98	M+2H+	H2HN3F1NA1C-PA	◆1		127122	2.03	
		3			1018.67	1018.41	M+2H+	H1HN2F1NA1C-PA	◆1		80507	1.29	
	pk.3-27	1	51.52	51.25-51.60	1099.22	1099.44	M+2H+	H2HN2F1NA1C-PA	◆1	512(H1HN1F1)	97388	1.56	Le^{X}
		2			1140.49	1140.47	M+2H+	HN4F1NA1C-PA	◆1	407(HN2)<br>739(HN2NA1)	67836	1.09	sLacdiNAc
	pk.3-28	1	52.02	51.67-52.50	849.68<br>1273.92	849.68<br>1274.01	M+3H+<br>M+2H+	H2HN3F2NA1C-PA	◆1	512(H1HN1F1)	1750081	28.00	Le^{X}
		2			1046.62	1046.92	M+2H+	H1HN3NA1C-PA	◆1		59442	0.95	
	pk.3-29	1	52.89	52.57-53.40	752.36<br>1127.78	752.30<br>1127.95	M+3H+<br>M+2H+	H2HN3NA1C-PA	◆1		799915	12.80	
	pk.3-30	1	53.71	53.47-54.17	1020.53<br>1529.60	1020.07<br>1529.60	M+3H+<br>M+2H+	H3HN4F3NA1C-PA	◆1	512(H1HN1F1)	322355	5.16	Le^{X}
	pk.3-31	1	55.91	55.14-56.74	800.97<br>1201.30	800.99<br>1200.98	M+3H+<br>M+2H+	H2HN3F1NA1C-PA	◆1		2875545	46.00	
		2			747.50<br>1120.05	746.97<br>1119.95	M+3H+<br>M+2H+	H1HN3F1NA1C-PA	◆1		224400	3.59	
	pk.3-32	1	58.22	57.85-58.54	923.12<br>1383.53	922.70<br>1383.55	M+3H+<br>M+2H+	H3HN4F1NA1C-PA	◆1		458690	7.34	
fr.4	pk.4-1	1	31.24	30.09-31.67				data not available			448754	7.18	
	pk.4-2	1	32.40	31.94-32.85	990.43<br>1484.95	990.39<br>1485.09	M+3H+<br>M+2H+	H2HN3F3NA2C-PA	◇2	816(H1HN1F1NA1)	356507	5.70	sLe^{X}
		2			1160.84	1160.79	M+3H+	H3HN4F4NA2C-PA	◇2	816(H1HN1F1NA1)	236719	3.79	sLe^{X}
		3			1452.85			data not available			148494	2.38	
	pk.4-3	1	33.33	32.92-33.75	1093.41	1093.10	M+3H+	H3HN3F4NA2C-PA	◇2	816(H1HN1F1NA1)	293527	4.70	sLe^{X}
		2			1111.55	1112.10	M+3H+	H3HN4F3NA2C-PA	◇2	816(H1HN1F1NA1)	222092	3.55	sLe^{X}
		3			1405.01			data not available			167062	2.67	
	pk.4-4	1	34.23	33.89-34.58	922.67<br>1383.20	922.70<br>1383.55	M+3H+<br>M+2H+	H2HN2F3NA2C-PA	◇2	816(H1HN1F1NA1)	346012	5.54	sLe^{X}
		2			1112.58	1112.10	M+3H+	H3HN4F3NA2C-PA	◇2	816(H1HN1F1NA1)	147736	2.36	sLe^{X}
		3			1093.15			data not available			117557	1.88	
	pk.4-5	1	35.37	35.00-35.90	1020.04	1020.06	M+3H+	H4HN4NA2C-PA	◇2		740614	11.85	
	pk.4-6	1	37.39	36.74-37.71	990.33<br>1484.70	990.39<br>1485.09	M+3H+<br>M+2H+	H2HN3F3NA2C-PA	◇2	816(H1HN1F1NA1)	1835724	29.37	sLe^{X}
	pk.4-7	1	38.10	37.85-38.61	947.01<br>1420.23	947.04<br>1420.05	M+3H+<br>M+2H+	H3HN3F1NA2C-PA	◇2		1146044	18.33	
	pk.4-8	1	39.14	38.68-39.58	1160.84	1160.79	M+3H+	H3HN4F4NA2C-PA	◇2	816(H1HN1F1NA1)	717849	11.48	sLe^{X}
		2			1069.23	1068.75	M+3H+	H4HN4F1NA2C-PA	◇2	731(H2HN2)<br>1035(H2HN2NA1)	439923	7.04	sLacNAc repeat
	pk.4-9	1	40.00	39.65-40.14	1068.82	1068.75	M+3H+	H4HN4F1NA2C-PA	◇2		561873	8.99	
		2			1190.27	1190.46	M+3H+	H5HN5F1NA2C-PA	◇2	731(H2HN2)<br>1035(H2HN2NA1)	130265	2.08	sLacNAc repeat
	pk.4-10	1	40.54	40.28-41.32	1068.69	1068.75	M+3H+	H4HN4F1NA2C-PA	◇2		714298	11.43	
		2			1190.45	1190.46	M+3H+	H5HN5F1NA2C-PA	◇2	731(H2HN2)<br>1035(H2HN2NA1)	394763	6.32	sLacNAc repeat
		3			1312.11	1312.17	M+3H+	H6HN6F1NA2C-PA	◇2	731(H2HN2)<br>1035(H2HN2NA1)	208180	3.33	sLacNAc repeat
	pk.4-11	1	42.01	41.60-42.15	1312.12	1312.17	M+3H+	H6HN6F1NA2C-PA	◇2	731(H2HN2)<br>1035(H2HN2NA1)	223389	3.57	sLacNAc repeat
		2			1190.63	1190.46	M+3H+	H5HN5F1NA2C-PA	◇2	731(H2HN2)<br>1035(H2HN2NA1)	146544	2.34	sLacNAc repeat
		3			1014.57	1014.73	M+3H+	H3HN4F1NA2C-PA	◇2		120074	1.92	

Table S2 Continued.

Fr. No. (DEAE)	Peak. No. (ODS)	Full MS No.	Elusion time max (min)	Elusion time range (min)	Observed parent ion (m/z value)	Calculated (m/z value)	Estimated adduct	Estimated composition[a), b), c)]		Characteristic fragments[a)]	Area[d)]	Relative amounts[e)]	Notes[f)]
	pk.4-12	1	43.88	43.40-44.58	946.93<br>1419.57	947.04<br>1420.05	M+3H$^+$<br>M+2H$^+$	H3HN3F1NA2C-PA	◇2	731(H2HN2)<br>1035(H2HN2NA1)	1077800	17.24	sLacNAc repeat
		2			1015.09	1014.73	M+3H$^+$	H3HN4F1NA2C-PA	◇2		573311	9.17	
		3			1087.58	1087.76	M+3H$^+$	H4HN5NA2C-PA	◇2		374011	5.98	
	pk.4-13	1	44.91	44.65-45.15	1257.88	1258.15	M+3H$^+$	H5HN6F1NA2C-PA	◇2	731(H2HN2)<br>1035(H2HN2NA1)	180555	2.89	sLacNAc repeat
		2			1068.67	1068.75	M+3H$^+$	H4HN4F1NA2C-PA	◇2		197390	3.16	
		3			1033.77			data not available			152031	2.43	
		4			946.69	947.04	M+3H$^+$	H3HN3F1NA2C-PA	◇2		114817	1.84	
	pk.4-14	1	45.52	45.21-46.18	1069.47	1068.75	M+3H$^+$	H4HN4F1NA2C-PA	◇2	1096(H3HN3)<br>1401(H3HN3NA1)	618384	9.89	sLacNAc repeat3
		2			1190.30	1190.46	M+3H$^+$	H5HN5F1NA2C-PA	◇2	1096(H3HN3)<br>1401(H3HN3NA1)	587970	9.41	sLacNAc repeat3
	pk.4-15	1	46.51	46.25-46.60	1312.24	1312.17	M+3H$^+$	H6HN6F1NA2C-PA	◇2	1462(H4HN4)<br>1766(H4HN4NA1)	179097	2.87	sLacNAc repeat4
		2			1190.94	1190.46	M+3H$^+$	H5HN5F1NA2C-PA	◇2	1096(H3HN3)<br>1401(H3HN3NA1)	81368	1.30	sLacNAc repeat3
		3			1068.88			data not available			77530	1.24	xMS2
	pk.4-16	1	48.24	46.88-48.68	1136.42	1136.44	M+3H$^+$	H4HN5F1NA2C-PA	◇2		2692527	43.07	
		2			1082.39	1082.43	M+3H$^+$	H3HN5F1NA2C-PA	◇2		551332	8.82	
		3			1258.42	1258.15	M+3H$^+$	H5HN6F1NA2C-PA	◇2	731(H2HN2)<br>1035(H2HN2NA1)	334797	5.36	sLacNAc repeat
	pk.4-17	1	49.05	48.75-49.24	1082.49	1082.43	M+3H$^+$	H3HN5F1NA2C-PA	◇2		647645	10.36	
		2			1028.76	1028.41	M+3H$^+$	H2HN5F1NA2C-PA	◇2		210478	3.37	
	pk.4-18	1	49.48	48.75-49.24	1015.28	1014.73	M+3H$^+$	H3HN4F1NA2C-PA	◇2	731(H2HN2)<br>1035(H2HN2NA1)	569494	9.11	sLacNAc repeat
		2			1082.39	1082.43	M+3H$^+$	H3HN5F1NA2C-PA	◇2		164187	2.63	
	pk.4-19	1	50.37	49.79-50.69	1136.33	1136.44	M+3H$^+$	H4HN5F1NA2C-PA	◇2	1096(H3HN3)<br>1401(H3HN3NA1)	636251	10.18	sLacNAc repeat3
		2			1200.33	1199.80	M+3H$^+$	H5HN5F1NA2C-PA	◇1◆1		318354	5.09	
		3			1258.00	1258.15	M+3H$^+$	H5HN6F1NA2C-PA	◇2	1096(H3HN3)<br>1401(H3HN3NA1)	305240	4.88	sLacNAc repeat3
		4			1121.49	1121.45	M+3H$^+$	H3HN4F3NA2C-PA	◇1◆1	816(H1HN1F1NA1)	150140	2.40	sLe^X
		5			956.46	956.38	M+3H$^+$	H3HN3F1NA2C-PA	◇1◆1		172473	2.76	
	pk.4-20	1	51.14	50.76-52.15	1199.64	1199.80	M+3H$^+$	H5HN5F1NA2C-PA	◇1◆1		900659	14.41	
		2			1078.13	1078.09	M+3H$^+$	H4HN4F1NA2C-PA	◇1◆1		391964	6.27	
		3			1217.72			data not available			323510	5.18	
		4			1054.22	1053.75	M+3H$^+$	H3HN3F3NA2C-PA	◇1◆1		380155	6.08	
		5			1014.58	1014.73	M+3H$^+$	H3HN4F1NA2C-PA	◇2		382417	6.12	
	pk.4-21	1	52.79	52.29-53.06	1014.99	1014.73	M+3H$^+$	H3HN4F1NA2C-PA	◇2		464568	7.43	
		2			1136.44			data not available			318618	5.10	
	pk.4-22	1	53.33	53.19-53.82	950.96<br>1267.72	950.87<br>1267.50	M+4H$^+$<br>M+3H$^+$	H5HN6F1NA2C-PA	◇1◆1	1096(H3HN3)<br>1401(H3HN3NA1)	542802	8.68	sLacNAc repeat3
	pk.4-23	1	54.33	54.03-54.72	951.09<br>1425.80	951.05<br>1426.07	M+3H$^+$<br>M+2H$^+$	H2HN3F2NA2C-PA	◇1◆1	816(H1HN1F1NA1)	1148930	18.38	sLe^X
		2			902.81	902.36	M+3H$^+$	H2HN3F1NA2C-PA	◇1◆1		211885	3.39	
	pk.4-24	1	55.01	54.86-55.14	1145.76	1145.79	M+3H$^+$	H4HN5F1NA2C-PA	◇1◆1		267889	4.29	
	pk.4-25	1	55.56	55.28-55.83	1121.94	1121.45	M+3H$^+$	H3HN4F3NA2C-PA	◇1◆1	816(H1HN1F1NA1)	540482	8.65	sLe^X
	pk.4-26	1	57.39	56.67-57.57	1192.53	1192.49	M+2H$^+$	H2HN2NA2C-PA	◆2		351993	5.63	
	pk.4-27	1	58.08	57.71-58.61	917.10<br>1375.02	917.04<br>1375.06	M+3H$^+$<br>M+2H$^+$	H3HN3NA2C-PA	◆2		611021	9.77	
	pk.4-28	1	60.20	59.44-60.42	1024.55	1024.08	M+3H$^+$	H3HN4F1NA2C-PA	◇1◆1		437420	7.00	
	pk.4-29	1	60.82	60.56-61.25	965.85<br>1447.78	965.73<br>1448.09	M+3H$^+$<br>M+2H$^+$	H3HN3F1NA2C-PA	◆2		293923	4.70	
	pk.4-30	1	64.23	63.96-64.86	911.75<br>1366.98	911.71<br>1367.06	M+3H$^+$<br>M+2H$^+$	H2HN3F1NA2C-PA	◆2		110830	1.77	
		2			966.22<br>1447.99	965.73<br>1448.09	M+3H$^+$<br>M+2H$^+$	H3HN3F1NA2C-PA	◆2		54226	0.87	
		3			1145.74	1145.79	M+3H$^+$	H4HN5F1NA2C-PA	◇1◆1		46890	0.75	
	pk.4-31	1	65.52	65.28-66.04	1033.38<br>1549.49	1033.42<br>1549.63	M+3H$^+$	H3HN4F1NA2C-PA	◆2		205542	3.29	
fr.5	pk.5-1	1	31.22	30.83-31.67				data not available			424603	6.79	
	pk.5-2	1	33.27	32.78-33.61	898.27<br>1347.23	898.35<br>1347.03	M+3H$^+$<br>M+2H$^+$	H3HN3NA2C-PA	◇2		407701	6.52	
	pk.5-3	1	37.94	37.50-38.26	947.32<br>1420.26	947.04<br>1420.05	M+3H$^+$<br>M+2H$^+$	H3HN3F1NA2C-PA	◇2		1269421	20.31	
		2			1237.26	1237.49	M+2H$^+$	H2HN2F1NA2C-PA	◇2		275842	4.41	
	pk.5-4	1	38.51	38.33-38.96	776.64<br>1164.10	776.64<br>1164.46	M+3H$^+$<br>M+2H$^+$	H2HN2NA2C-PA	◇2		793118	12.69	
	pk.5-5	1	42.26	41.67-42.64	1154.88			data not available			415205	6.64	
		2			1245.42	1245.49	M+2H$^+$	H2HN2F1NA1NG1C-PA	◇2		57008	0.91	
	pk.5-6	1	43.93	43.47-44.44	825.34<br>1237.66	825.33<br>1237.49	M+3H$^+$<br>M+2H$^+$	H2HN2F1NA2C-PA	◇2		3974207	63.58	
	pk.5-7	1	44.93	44.72-45.42	1237.03	1237.49	M+2H$^+$	H2HN2F1NA2C-PA	◇2		250244	4.00	

Table S2 Continued.

Fr. No. (DEAE)	Peak. No. (ODS)	Full MS No.	Elusion time max (min)	Elusion time range (min)	Observed parent ion (m/z value)	Calculated (m/z value)	Estimated adduct	Estimated composition[a), b), c)]		Characteristic fragments[a)]	Area[d)]	Relative amounts[e)]	Notes[f)]
		2			1266.51	1266.00	$M+2H^+$	H2HN3NA2C-PA	◇2		145776	2.33	
	pk.5-8	1	48.86	48.61-49.17	1178.46	1178.48	$M+2H^+$	H2HN2NA2C-PA	◇1◆1		177902	2.85	
		2			1227.62			data not available			174750	2.80	
	pk.5-9	1	49.79	49.51-50.14	892.97<br>1338.60	893.02<br>1339.03	$M+3H^+$<br>$M+2H^+$	H2HN3F1NA2C-PA	◇2		959132	15.34	
	pk.5-10	1	50.61	50.28-50.90	785.80<br>1178.65	785.99<br>1178.48	$M+3H^+$<br>$M+2H^+$	H2HN2NA2C-PA	◇1◆1		703976	11.26	
		2			956.46	956.38	$M+3H^+$	H3HN3F1NA2C-PA	◇1◆1		75349	1.21	
		3			1251.41	1251.50	$M+2H^+$	H2HN2F1NA2C-PA	◇1◆1		62509	1.00	
	pk.5-11	1	51.38	51.04-51.67	902.19<br>1353.18	902.36<br>1353.04	$M+3H^+$<br>$M+2H^+$	H2HN3F1NA2C-PA	◇1◆1		283640	4.54	
		2			1324.89	1324.53	$M+2H^+$	H2HN2F2NA2C-PA	◇1◆1	816(H1HN1F1NA1)	238069	3.81	sLe^X
	pk.5-12	1	52.07	51.74-52.36	908.01<br>1361.29	907.70<br>1361.04	$M+3H^+$<br>$M+2H^+$	H3HN3NA2C-PA	◇1◆1		497461	7.96	
		2			1251.31	1251.50	$M+2H^+$	H2HN2F1NA2C-PA	◇1◆1		111155	1.78	
	pk.5-13	1	52.90	52.57-53.19	794.70<br>1191.47	795.33<br>1192.49	$M+3H^+$<br>$M+2H^+$	H2HN2NA2C-PA	◆2		304527	4.87	
		2			1014.94	1014.73	$M+3H^+$	H3HN4F1NA2C-PA	◇2		99782	1.60	
	pk.5-14	1	53.72	53.26-54.03	834.84<br>1251.91	834.67<br>1251.50	$M+3H^+$<br>$M+2H^+$	H2HN2F1NA2C-PA	◇1◆1	816(H1HN1F1NA1)	729870	11.68	sLe^X
		2			795.50<br>1192.85	795.33<br>1192.49	$M+3H^+$<br>$M+2H^+$	H2HN2NA2C-PA	◆2		495686	7.93	
	pk.5-15	1	54.51	54.17-54.93	951.62<br>1426.42	951.05<br>1426.07	$M+3H^+$<br>$M+2H^+$	H2HN3F2NA2C-PA	◇1◆1	816(H1HN1F1NA1)	964883	15.44	sLe^X
		2			902.57	902.36	$M+3H^+$	H2HN3F1NA2C-PA	◇1◆1		151414	2.42	
	pk.5-16	1	57.52	57.15-57.85	795.33<br>1192.50	795.33<br>1192.49	$M+3H^+$<br>$M+2H^+$	H2HN2NA2C-PA	◆2		6251184	100.00	
	pk.5-17	1	58.18	57.92-58.68	917.19<br>1375.52	917.04<br>1375.06	$M+3H^+$<br>$M+2H^+$	H3HN3NA2C-PA	◆2		634019	10.14	
		2			902.20<br>1352.74	902.36<br>1353.04	$M+3H^+$<br>$M+2H^+$	H2HN3F1NA2C-PA	◇1◆1		337323	5.40	
		3			795.31<br>1192.44	795.33<br>1192.49	$M+3H^+$<br>$M+2H^+$	H2HN2NA2C-PA	◆2		253140	4.05	
	pk.5-18	1	60.37	60.00-60.76	843.98<br>1265.30	844.02<br>1265.52	$M+3H^+$<br>$M+2H^+$	H2HN2F1NA2C-PA	◆2		2144985	34.31	
		2			1024.38	1024.08	$M+3H^+$	H3HN4F1NA2C-PA	◇1◆1		100718	1.61	
	pk.5-19	1	61.25	60.97-61.46	862.94<br>1293.79	863.02<br>1294.03	$M+3H^+$<br>$M+2H^+$	H2HN3NA2C-PA	◆2		128843	2.06	
		2			1265.38	1265.52	$M+2H^+$	H2HN2F1NA2C-PA	◆2		59726	0.96	
	pk.5-20	1	64.25	63.82-64.72	911.71<br>1367.33	911.71<br>1367.06	$M+3H^+$<br>$M+2H^+$	H2HN3F1NA2C-PA	◆2		581399	9.30	
fr.6	pk.6-1	1	31.01	30.53-31.56				data not available			425200	6.80	
	pk.6-2	1	33.34	32.74-33.56	1308.44	1307.97	$M+2H^+$	H3HN3F1NA1(SO3)1C-PA	◇1		167874	2.69	
		2			1535.14			data not available			71562	1.14	
	pk.6-3	1	33.98	33.63-34.25	1308.57	1307.97	$M+2H^+$	H3HN3F1NA1(SO3)1C-PA	◇1		285218	4.56	
	pk.6-4	1	37.26	36.80-37.49	1308.25	1307.97	$M+2H^+$	H3HN3F1NA1(SO3)1C-PA	◇1	731(H2HN2)<br>811(H2HN2(SO3)1)	157861	2.53	LacNAc repeat(SO3)
		2			1236.88			data not available			60146	0.96	
	pk.6-5	1	37.79	37.56-38.04	751.15<br>1125.16	750.60<br>1125.40	$M+3H^+$<br>$M+2H^+$	H2HN2F1NA1(SO3)1C-PA	◇1		279605	4.47	
	pk.6-6	1	38.19	38.18-38.53	1164.57	1164.46	$M+2H^+$	H2HN2NA2C-PA	◇2		131089	2.10	
	pk.6-7	1	38.98	38.59-39.28	994.08	994.03	$M+3H^+$	H4HN4F1NA1(SO3)1C-PA	◇1	731(H2HN2)<br>811(H2HN2(SO3)1)	201774	3.23	LacNAc repeat(SO3)
	pk.6-8	1	40.24	39.83-40.52	1116.12			data not available			184761	2.96	
	pk.6-9	1	42.07	41.70-42.45	1154.80			data not available			148230	2.37	
	pk.6-10	1	43.52	43.07-43.83	825.61<br>1237.77	825.33<br>1237.49	$M+3H^+$<br>$M+2H^+$	H2HN2F1NA2C-PA	◇2		369051	5.90	
	pk.6-11	1	44.91	44.52-45.42	989.35			data not available			129692	2.07	
		2			1178.60	1178.48	$M+2H^+$	H2HN2NA2C-PA	◇1◆1	698(H1HN1NA1)<br>1002(H1HN1NA2)	112466	1.80	α2,3, α2,6NA<br>LacNAc
	pk.6-12	1	46.24	45.55-46.73	1152.30<br>1535.49	1151.69<br>1535.26	$M+4H^+$<br>$M+3H^+$	H7HN7F1NA3C-PA	◇3	1096(H3HN3)<br>1401(H3HN3NA1)	368399	5.89	sLacNAc repeat3
		2			1243.30	1242.98	$M+4H^+$	H8HN8F1NA3C-PA	◇3	731(H2HN2)<br>1035(H2HN2NA1)	82361	1.32	sLacNAc repeat
		3			1061.73	1060.41	$M+4H^+$	H6HN6F1NA3C-PA	◇3	731(H2HN2)<br>1035(H2HN2NA1)	106036	1.70	sLacNAc repeat
	pk.6-13	1	48.65	48.45-49.07	834.85<br>1251.85	834.67<br>1251.50	$M+3H^+$<br>$M+2H^+$	H2HN2F1NA2C-PA	◇1◆1		218408	3.49	
	pk.6-14	1	49.75	49.35-49.83	1253.49	1253.47	$M+2H^+$	HN4F2NA1(SO3)1C-PA	◆1	407(HN2)<br>739(HN2NA1)	70181	1.12	sLacdiNAc
		2			1139.30			data not available			36925	0.59	
		3			1179.07			data not available			34902	0.56	xMS2
	pk.6-15	1	50.06	49.90-49.07	786.45<br>1178.80	785.99<br>1178.48	$M+3H^+$<br>$M+2H^+$	H2HN2NA2C-PA	◇1◆1		134810	2.16	
	pk.6-16	1	50.78	50.31-51.07	1203.16			data not available			300597	4.81	
	pk.6-17	1	53.21	52.86-53.76	795.91<br>1192.24	795.33<br>1192.49	$M+3H^+$<br>$M+2H^+$	H2HN2NA2C-PA	◆2		155951	2.49	
		2			1251.16	1251.50	$M+2H^+$	H2HN2F1NA2C-PA	◇1◆1		49699	0.80	
		3			1313.79	1313.99	$M+2H^+$	H2HN3F2NA1(SO3)1C-PA	◆1		57166	0.91	
	pk.6-18	1	56.92	56.51-57.48	795.26<br>1192.79	795.33<br>1192.49	$M+3H^+$<br>$M+2H^+$	H2HN2NA2C-PA	◆2		807000	12.91	

Table S2 Continued.

Fr. No. (DEAE)	Peak. No. (ODS)	Full MS No.	Elusion time max (min)	Elusion time range (min)	Observed parent ion (m/z value)	Calculated (m/z value)	Estimated adduct	Estimated composition[a), b), c)]		Characteristic fragments[a)]	Area[d)]	Relative amounts[e)]	Notes[f)]
	pk.6-19	1	59.74	59.34-60.10	844.04<br>1265.75	844.02<br>1265.52	$M+3H^+$<br>$M+2H^+$	H2HN2F1NA2C-PA	◆2		99893	1.60	
fr.7	pk.7-1	1	12.53	12.06-12.96	777.88	778.26	$M+2H^+$	H3(HPO3)1C-PA		405(H2(HPO3)1)	51623	0.83	Man-6-P
	pk.7-2	1	13.52	13.09-13.92	778.11	778.26	$M+2H^+$	H3(HPO3)1C-PA		405(H2(HPO3)1)	85724	1.37	Man-6-P
	pk.7-3	1	17.03	16.47-17.64	784.68			data not available			240846	3.85	
	pk.7-4	1	17.96	17.78-18.13	777.92	778.26	$M+2H^+$	H3(HPO3)1C-PA		405(H2(HPO3)1)	24864	0.40	Man-6-P
	pk.7-5	1	18.51	18.19-19.09	784.43			data not available			65130	1.04	
	pk.7-6	1	27.19	26.95-27.57	800.66<br>1200.79	800.60<br>1200.40	$M+3H^+$<br>$M+2H^+$	HN4F3(SO3)2C-PA		633(HN2F1(SO3)1)	66869	1.07	LacdiNAc(SO3)F
	pk.7-7	1	33.37	32.94-33.63	1125.16	1125.40	$M+2H^+$	H2HN2F1NA1(SO3)1C-PA	◇1		151568	2.42	
	pk.7-8	1	34.18	33.70-34.60	1433.00			data not available			191403	3.06	
		2			1052.58			data not available			118414	1.89	
	pk.7-9	1	35.12	34.67-35.63	1384.01			data not available			311331	4.98	
	pk.7-10	1	36.08	35.70-36.32	1262.43	1262.17	$M+3H^+$	H3HN4F4NA3C-PA	◇3	816(H1HN1F1NA1)	169222	2.71	sLe^X
	pk.7-11	1	37.28	36.87-37.56	872.60<br>1307.52	872.32<br>1307.97	$M+3H^+$<br>$M+2H^+$	H3HN3F1NA1(SO3)1C-PA	◇1	731(H2HN2)	188815	3.02	LacNAc repeat
		2			1145.57	1145.92	$M+2H^+$	H1HN3F1NA1(SO3)1C-PA	◇1	487(HN2(SO3)1)	110679	1.77	LacdiNAc(SO3)
	pk.7-12	1	38.00	37.70-38.46	1194.83	1194.47	$M+3H^+$	H3HN3F4NA3C-PA	◇3	816(H1HN1F1NA1)	348474	5.57	sLe^X
		2			1125.06	1125.40	$M+2H^+$	H2HN2F1NA1(SO3)1C-PA	◇1		60215	0.96	
	pk.7-13	1	39.21	38.54-39.83	750.89<br>1124.93	750.60<br>1125.40	$M+3H^+$<br>$M+2H^+$	H2HN2F1NA1(SO3)1C-PA	◇1		737473	11.80	
	pk.7-14	1	42.28	41.70-42.73	947.08<br>1262.40	946.88<br>1262.17	$M+4H^+$<br>$M+3H^+$	H3HN4F4NA3C-PA	◇3	816(H1HN1F1NA1)	667223	10.67	sLe^X
	pk.7-15	1	44.37	43.90-44.52	969.13<br>1291.84	969.13<br>1291.84	$M+4H^+$<br>$M+3H^+$	H5HN5F1NA3C-PA	◇3	731(H2HN2)<br>1035(H2HN2NA1)	240636	3.85	sLacNAc repeat
	pk.7-16	1	45.61	44.59-46.86	1060.43<br>1414.26	1060.41<br>1413.55	$M+4H^+$<br>$M+3H^+$	H6HN6F1NA3C-PA	◇3	731(H2HN2)<br>1035(H2HN2NA1)	734024	11.74	sLacNAc repeat
		2			1292.10	1291.84	$M+3H^+$	H5HN5F1NA3C-PA	◇3	731(H2HN2)<br>1035(H2HN2NA1)	713571	11.41	sLacNAc repeat
	pk.7-17	1	47.72	46.93-48.38	1066.00	1066.39	$M+2H^+$	H2HN2NA1(SO3)1C-PA	◆1		571741	9.15	
	pk.7-18	1	50.39	49.76-50.93	1019.65<br>1360.15	1019.90<br>1359.53	$M+4H^+$<br>$M+3H^+$	H5HN6F1NA3C-PA	◇3	731(H2HN2)<br>1035(H2HN2NA1)	546484	8.74	sLacNAc repeat
		2			1111.15<br>1481.84	1111.18<br>1481.24	$M+4H^+$<br>$M+3H^+$	H6HN7F1NA3C-PA	◇3	731(H2HN2)<br>1035(H2HN2NA1)	274247	4.39	sLacNAc repeat
		3			1237.74	1237.82	$M+3H^+$	H4HN5F1NA3C-PA	◇3		121915	1.95	
	pk.7-19	1	51.21	51.07-51.69	760.09<br>1139.63	759.95<br>1139.42	$M+3H^+$<br>$M+2H^+$	H2HN2F1NA1(SO3)1C-PA	◆1	778(H1HN1NA1(SO3)1)	424858	6.80	sLacNAc(SO3)
	pk.7-20	1	52.52	52.24-52.65	1301.06	1301.18	$M+3H^+$	H5HN5F1NA3C-PA	◇2◆1	731(H2HN2)<br>1035(H2HN2NA1)	107232	1.72	sLacNAc repeat
		2			1423.03	1422.89	$M+3H^+$	H6HN6F1NA3C-PA	◇2◆1	1035(H2HN2NA1)	83846	1.34	sLacNAc repeat
	pk.7-21	1	52.99	52.79-53.34	1423.16	1422.89	$M+3H^+$	H6HN6F1NA3C-PA	◇2◆1	731(H2HN2)<br>1035(H2HN2NA1)	166848	2.67	sLacNAc repeat
		2			1301.62	1301.18	$M+3H^+$	H5HN5F1NA3C-PA	◇2◆1	731(H2HN2)<br>1035(H2HN2NA1)	76373	1.22	sLacNAc repeat
	pk.7-22	1	54.09	53.62-54.17	1237.86			data not available			153762	2.46	
	pk.7-23	1	55.02	54.86-55.41	1369.11			data not available			129762	2.08	
	pk.7-24	1	56.66	56.24-57.00	1222.62	1222.82	$M+3H^+$	H3HN4F3NA3C-PA	◇2◆1	816(H1HN1F1NA1)	208519	3.34	sLe^X
	pk.7-25	1	57.44	57.13-57.82				data not available			107104	1.71	
fr.8	pk.8-1	1	27.18	26.88-27.50	800.42<br>1200.56	800.60<br>1200.40	$M+3H^+$<br>$M+2H^+$	HN4F3(SO3)2C-PA		633(HN2F1(SO3)1)	101586	1.63	LacdiNAc(SO3)F
	pk.8-2	1	32.86	32.57-33.40				data not available			196506	3.14	
	pk.8-3	1	34.43	33.75-34.79				data not available			234435	3.75	
	pk.8-4	1	36.72	36.39-37.08				data not available			217716	3.48	
	pk.8-5	1	37.55	37.15-38.06	1000.13<br>1499.16	999.73<br>1499.09	$M+3H^+$<br>$M+2H^+$	H3HN3NA3C-PA	◇3		376897	6.03	
		2			1194.41	1194.47	$M+3H^+$	H3HN3F4NA3C-PA	◇3	816(H1HN1F1NA1)	95998	1.54	sLe^X
	pk.8-6	1	39.83	39.17-40.49	841.72<br>1121.58	841.33<br>1121.44	$M+4H^+$<br>$M+3H^+$	H4HN4NA3C-PA	◇3		902258	14.43	
		2			1048.55	1048.41	$M+3H^+$	H3HN3F1NA3C-PA	◇3		82199	1.31	
	pk.8-7	1	41.01	40.69-41.39	1067.33	1067.42	$M+3H^+$	H3HN4NA3C-PA	◇3		159774	2.56	
		2			1169.81	1170.13	$M+3H^+$	H4HN4F1NA3C-PA	◇3		163922	2.62	
	pk.8-8	1	41.89	41.53-42.36	1262.47	1262.17	$M+3H^+$	H3HN4F4NA3C-PA	◇3	816(H1HN1F1NA1)	225484	3.61	sLe^X
		2			1170.36	1170.13	$M+3H^+$	H4HN4F1NA3C-PA	◇3		122026	1.95	
	pk.8-9	1	42.98	42.50-43.82	1048.39<br>1572.22	1048.41<br>1572.12	$M+3H^+$<br>$M+2H^+$	H3HN3F1NA3C-PA	◇3		1933192	30.93	
	pk.8-10	1	44.92	44.03-45.49	877.86<br>1170.67	877.85<br>1170.13	$M+3H^+$<br>$M+2H^+$	H4HN4F1NA3C-PA	◇3		1698555	27.17	
		2			1291.62	1291.84	$M+3H^+$	H5HN5F1NA3C-PA	◇3	731(H2HN2)<br>1035(H2HN2NA1)	170301	2.72	sLacNAc repeat
	pk.8-11	1	45.88	45.69-46.60				data not available			453290	7.25	
	pk.8-12	1	47.66	46.94-48.33	1189.37	1189.13	$M+3H^+$	H4HN5NA3C-PA	◇3		435194	6.96	
		2			1115.86	1116.11	$M+3H^+$	H3HN4F1NA3C-PA	◇3		277338	4.44	

Table S2 Continued.

Fr. No. (DEAE)	Peak. No. (ODS)	Full MS No.	Elusion time max (min)	Elusion time range (min)	Observed parent ion (m/z value)	Calculated (m/z value)	Estimated adduct	Estimated composition[a), b), c)]		Characteristic fragments[a)]	Area[d)]	Relative amounts[e)]	Notes[f)]
		3			1135.05			data not available			239768	3.84	
	pk.8-13	1	49.31	49.31-48.40	1048.88	1048.41	M+3H$^+$	H3HN3F1NA3C-PA	◇3		574861	9.20	
		2			1228.20			data not available			606342	9.70	
	pk.8-14	1	50.55	49.86-51.18	928.88<br>1237.79	928.62<br>1237.82	M+4H$^+$<br>M+3H$^+$	H4HN5F1NA3C-PA	◇3		1515860	24.25	
		2			1184.27	1183.80	M+3H$^+$	H3HN5F1NA3C-PA	◇3		310481	4.97	
	pk.8-15	1	51.51	51.25-52.01	1184.04	1183.80	M+3H$^+$	H3HN5F1NA3C-PA	◇3		426746	6.83	
		2			1067.43	1067.42	M+3H$^+$	H3HN4NA3C-PA	◇3		163247	2.61	
	pk.8-16	1	52.52	52.15-52.78	1301.46	1301.18	M+3H$^+$	H5HN5F1NA3C-PA	◇2◆1		376105	6.02	
		2			1223.29	1222.82	M+3H$^+$	H3HN4F3NA3C-PA	◇2◆1	816(H1HN1F1NA1)	177665	2.84	sLe^X
	pk.8-17	1	53.09	52.85-53.96	1058.03	1057.76	M+3H$^+$	H3HN3F1NA3C-PA	◇2◆1		303675	4.86	
		2			1179.33	1179.47	M+3H$^+$	H4HN4F1NA3C-PA	◇2◆1		363328	5.81	
		3			1155.18	1155.13	M+3H$^+$	H3HN3F3NA3C-PA	◇2◆1	816(H1HN1F1NA1)	185458	2.97	sLe^X
		4			1301.05	1301.18	M+3H$^+$	H5HN5F1NA3C-PA	◇2◆1		242911	3.89	
	pk.8-18	1	54.34	54.10-54.79	1115.96	1116.11	M+3H$^+$	H3HN4F1NA3C-PA	◇3		502329	8.04	
	pk.8-19	1	55.17	54.86-55.42	1368.88	1368.87	M+3H$^+$	H5HN6F1NA3C-PA	◇2◆1		198993	3.18	
		2			1198.32	1198.48	M+3H$^+$	H4HN5NA3C-PA	◇2◆1		82110	1.31	
	pk.8-20	1	55.90	55.63-56.32	1125.37	1125.45	M+3H$^+$	H3HN4F1NA3C-PA	◇2◆1		352900	5.65	
	pk.8-21	1	56.70	56.39-56.88	1223.21	1222.82	M+3H$^+$	H3HN4F3NA3C-PA	◇2◆1	816(H1HN1F1NA1)	368597	5.90	sLe^X
	pk.8-22	1	57.28	57.01-57.71	1247.07	1247.16	M+3H$^+$	H4HN5F1NA3C-PA	◇2◆1		385685	6.17	
	pk.8-23	1	59.71	59.17-60.00	1018.19	1018.42	M+3H$^+$	H3HN3NA3C-PA	◇1◆2		1029677	16.47	
	pk.8-24	1	61.38	60.97-61.67	1125.41	1125.45	M+3H$^+$	H3HN4F1NA3C-PA	◇2◆1		196558	3.14	
	pk.8-25	1	62.32	61.74-62.78	1066.93	1067.10	M+3H$^+$	H3HN3F1NA3C-PA	◇1◆2		352019	5.63	
	pk.8-26	1	65.49	65.00-65.97	1027.64<br>1540.78	1027.76<br>1541.14	M+3H$^+$<br>M+2H$^+$	H3HN3NA3C-PA	◆3		241521	3.86	
		2			1183.60	1183.48	M+3H$^+$	H3HN4F2NA3C-PA	◇1◆2		30445	0.49	
	pk.8-27	1	66.58	66.25-67.01	1134.75	1134.80	M+3H$^+$	H3HN4F1NA3C-PA	◇1◆2		107783	1.72	
	pk.8-28	1	68.01	67.71-68.26	1077.05<br>1614.35	1076.45<br>1614.17	M+3H$^+$<br>M+2H$^+$	H3HN3F1NA3C-PA	◆3		63174	1.01	
	pk.8-29	1	71.58	71.39-71.88	1144.89	1144.14	M+3H$^+$	H3HN4F1NA3C-PA	◆3		22743	0.36	
fr.9	pk.9-1	1	33.73	33.49-34.18	1054.61	1054.34	M+2H$^+$	HN4F1(SO3)2C-PA		407(HN2)<br>487(HN2(SO3)1)	129847	2.08	LacdiNAc(SO3)
	pk.9-2	1	36.98	36.46-37.28	1636.40	1636.63	M+3H$^+$	H7HN7F1NA4C-PA	◇4		170984	2.74	
	pk.9-3	1	37.99	37.35-38.11				data not available			272208	4.35	
	pk.9-4	1	38.64	38.18-39.63	973.98<br>1459.52	973.69<br>1460.03	M+3H$^+$<br>M+2H$^+$	H3HN3F1NA2(SO3)1C-PA	◇2		686226	10.98	
	pk.9-5	1	40.22	39.90-40.80				data not available			199325	3.19	
	pk.9-6	1	41.98	41.42-43.76	973.68<br>1460.22	973.69<br>1460.03	M+3H$^+$<br>M+2H$^+$	H3HN3F1NA2(SO3)1C-PA	◇2	811(H2HN2(SO3)1)<br>1115(H2HN2NA1(SO3)1)	318645	5.10	LacNAc repeat
	pk.9-7	1	43.31	42.73-43.76	1095.50	1095.40	M+3H$^+$	H4HN4F1(SO3)1NA2C-PA	◇2	811(H2HN2(SO3)1)<br>1115(H2HN2NA1(SO3)1)	200811	3.21	LacNAc repeat
	pk.9-8	1	44.13	43.83-44.52	813.31			data not available			129676	2.07	
	pk.9-9	1	45.18	44.59-45.90	1344.23			data not available			364897	5.84	
	pk.9-10	1	47.40	46.93-47.76	1534.25			data not available			212870	3.41	
	pk.9-11	1	48.85	48.38-49.69	1045.31<br>1393.15	1045.16<br>1393.21	M+4H$^+$<br>M+3H$^+$	H5HN5F1NA4C-PA	◇4	731(H2HN2)<br>1035(H2HN2NA1)	568315	9.09	sLacNAc repeat
		2			1136.68<br>1514.86	1136.44<br>1514.92	M+4H$^+$<br>M+3H$^+$	H6HN6F1NA4C-PA	◇4	731(H2HN2)<br>1035(H2HN2NA1)	538040	8.61	sLacNAc repeat
	pk.9-12	1	50.60	49.90-50.93	1402.85			data not available			393797	6.30	
	pk.9-13	1	51.78	51.34-52.17	1597.06			data not available			276967	4.43	
	pk.9-14	1	52.73	52.24-53.62	1460.98			data not available			888175	14.21	
	pk.9-15	1	55.17	54.31-55.48	1402.67	1402.56	M+3H$^+$	H5HN5F1NA4C-PA	◇3◆1	731(H2HN2)<br>1035(H2HN2NA1)	397851	6.36	sLacNAc repeat
	pk.9-16	1	55.67	55.62-56.44	1402.90	1402.56	M+3H$^+$	H5HN5F1NA4C-PA	◇3◆1	731(H2HN2)<br>1035(H2HN2NA1)	202544	3.24	sLacNAc repeat
	pk.9-17	1	57.00	56.79-57.34	1192.26			data not available			99032	1.58	
	pk.9-18	1	58.40	58.10-58.65				data not available			96940	1.55	
	pk.9-19	1	59.17	58.92-59.61	1011.72<br>1348.99	1011.66<br>1348.54	M+4H$^+$<br>M+3H$^+$	H4HN5F1NA4C-PA	◇3◆1		128623	2.06	
fr.10	pk.10-1	1	31.12	30.67-31.63				data not available			176810	2.83	
	pk.10-2	1	33.58	33.36-34.25	1246.02			data not available			105214	1.68	
	pk.10-3	1	34.68	34.46-35.29	1020.55			data not available			105945	1.69	
	pk.10-4	1	36.27	35.70-36.67	1142.22			data not available			170760	2.73	
	pk.10-5	1	37.75	37.29-38.11				data not available			135674	2.17	

Table S2 Continued.

Fr. No. (DEAE)	Peak. No. (ODS)	Full MS No.	Elusion time max (min)	Elusion time range (min)	Observed parent ion (m/z value)	Calculated (m/z value)	Estimated adduct	Estimated composition[a), b), c)]		Characteristic fragments[a)]	Area[d)]	Relative amounts[e)]	Notes[f)]
	pk.10-6	1	38.64	38.18-39.42	1277.54	1277.47	$M+2H^{+}$	H2HN2F1NA2(SO3)1C-PA	◇2		142867	2.29	
		2			1204.43	1204.44	$M+2H^{+}$	H2HN2NA2(SO3)1C-PA	◇2		117795	1.88	
	pk.10-7	1	40.67	40.25-41.21	1738.46			data not available			241631	3.87	
	pk.10-8	1	42.06	41.56-42.73	1355.45<br>1806.22	1355.52<br>1807.03	$M+4H^{+}$<br>$M+3H^{+}$	H8HN8F2NA4C-PA	◇4	816(H1HN1F1NA1)	250962	4.01	sLe^{X}
		2			1318.38<br>1757.13	1319.01<br>1758.34	$M+4H^{+}$<br>$M+3H^{+}$	H8HN8F1NA4C-PA	◇4		141044	2.26	
	pk.10-9	1	44.20	43.76-44.59	851.97<br>1277.47	851.98<br>1277.47	$M+3H^{+}$<br>$M+2H^{+}$	H2HN2F1NA2(SO3)1C-PA	◇2	750(H1HN1NA1(SO3)1)	941418	15.06	sLacNAc(SO3)
	pk.10-10	1	44.78	44.66-45.35	917.47<br>1223.34	917.36<br>1222.81	$M+4H^{+}$<br>$M+3H^{+}$	H4HN4NA4C-PA	◇4		341529	5.46	
	pk.10-11	1	45.99	45.63-46.38	1271.90	1271.50	$M+3H^{+}$	H4HN4F1NA4C-PA	◇4		227094	3.63	
	pk.10-12	1	46.64	46.45-47.00	1271.11	1271.50	$M+3H^{+}$	H4HN4F1NA4C-PA	◇4		118806	1.90	
	pk.10-13	1	47.77	47.14-48.11	1232.49	1232.16	$M+3H^{+}$	H4HN4NA4C-PA	◇3◆1	1002(H1HN1NA2)	244720	3.91	α2,3, α2,6NA LacNAc
	pk.10-14	1	48.63	48.18-49.28	954.53<br>1271.78	953.88<br>1271.50	$M+4H^{+}$<br>$M+3H^{+}$	H4HN4F1NA4C-PA	◇4		1246593	19.94	
	pk.10-15	1	50.71	49.90-50.93	1281.12	1280.84	$M+3H^{+}$	H4HN4F1NA4C-PA	◇3◆1	1002(H1HN1NA2)	495668	7.93	α2,3, α2,6NA LacNAc
	pk.10-16	1	51.22	51.00-51.83	1419.62			data not available			298481	4.77	
	pk.10-17	1	52.74	52.31-53.21	1399.56	1339.19	$M+3H^{+}$	H4HN5F1NA4C-PA	◇4		561383	8.98	
	pk.10-18	1	53.60	53.34-53.90	1232.61	1232.16	$M+3H^{+}$	H4HN4NA4C-PA	◇3◆1		168363	2.69	
		2			1035.29			data not available			109970	1.76	
	pk.10-19	1	54.24	54.03-54.79	1291.69	1291.48	$M+2H^{+}$	H2HN2F1NA2(SO3)1C-PA	◇1◆1		358802	5.74	
	pk.10-20	1	55.11	54.86-55.27	1325.26<br>1767.03	1326.02<br>1767.69	$M+4H^{+}$<br>$M+3H^{+}$	H8HN8F1NA4C-PA	◇3◆1		171987	2.75	
	pk.10-21	1	55.57	55.34-55.89	1280.64	1280.84	$M+3H^{+}$	H4HN4F1NA4C-PA	◇3◆1		258913	4.14	
	pk.10-22	1	56.11	55.96-56.38	1281.12	1280.84	$M+3H^{+}$	H4HN4F1NA4C-PA	◇3◆1		155175	2.48	
	pk.10-23	1	56.66	56.45-57.20	1281.01	1280.84	$M+3H^{+}$	H4HN4F1NA4C-PA	◇3◆1	1002(H1HN1NA2)	172929	2.77	α2,3, α2,6NA LacNAc
	pk.10-24	1	57.57	57.27-57.69	1290.57	1290.19	$M+3H^{+}$	H4HN4F1NA4C-PA	◇2◆2	1002(H1HN1NA2)	108636	1.74	α2,3, α2,6NA LacNAc
	pk.10-25	1	57.92	57.82-58.24	1232.75	1232.47	$M+2H^{+}$	H2HN2NA2(SO3)1C-PA	◆2		103637	1.66	
	pk.10-26	1	59.12	58.93-59.96	1348.50	1348.54	$M+3H^{+}$	H4HN5F1NA4C-PA	◇3◆1		211509	3.38	
	pk.10-27	1	60.91	60.65-61.41	870.70<br>1305.28	870.67<br>1305.50	$M+3H^{+}$<br>$M+2H^{+}$	H2HN2F1NA2(SO3)1C-PA	◆2	778(H1HN1NA1(SO3)1)	186501	2.98	sLacNAc(SO3)
fr.11	pk.11-1	1	31.13	30.81-31.29				data not available			21634	0.35	
	pk.11-2	1	35.02	34.74-35.43				data not available			39584	0.63	
	pk.11-3	1	37.12	36.94-37.35	1164.97			data not available			41316	0.66	
	pk.11-4	1	37.98	37.56-38.11				data not available			59424	0.95	
	pk.11-5	1	38.45	38.18-38.87				data not available			75724	1.21	
	pk.11-6	1	40.74	40.32-41.28				data not available			115588	1.85	
	pk.11-7	1	41.86	41.49-42.39				data not available			88208	1.41	
	pk.11-8	1	43.26	42.52-43.49				data not available			85359	1.37	
	pk.11-9	1	44.32	43.63-44.87	1075.34	1075.07	$M+3H^{+}$	H3HN3F1NA3(SO3)1C-PA	◇3		520350	8.32	
	pk.11-10	1	45.48	45.14-45.97				data not available			92649	1.48	
	pk.11-11	1	46.38	46.04-46.93				data not available			91091	1.46	
	pk.11-12	1	50.05	49.76-50.24	1179.03	1179.40	$M+2H^{+}$	H2HN2F1NA1(SO3)2C-PA	◆1		96814	1.55	
	pk.11-13	1	50.77	50.31-51.00	1000.87<br>1333.84	1000.40<br>1333.53	$M+4H^{+}$<br>$M+3H^{+}$	H4HN4NA5C-PA	◇4◆1	1002(H1HN1NA2)	179924	2.88	α2,3, α2,6NA LacNAc
	pk.11-14	1	53.29	52.59-53.69	1036.94<br>1381.96	1036.92<br>1382.22	$M+4H^{+}$<br>$M+3H^{+}$	H4HN4F1NA5C-PA	◇4◆1	536(HN1NA1)<br>1002(H1HN1NA2)	328331	5.25	α2,3, α2,6NA LacNAc
		2			1128.06<br>1504.15	1128.20<br>1503.93	$M+4H^{+}$<br>$M+3H^{+}$	H5HN5F1NA5C-PA	◇4◆1	1002(H1HN1NA2)<br>1035(H2HN2NA1)	144713	2.31	α2,3, α2,6NA LacNAc
	pk.11-15	1	53.99	53.83-54.72	1084.32			data not available			164437	2.63	
	pk.11-16	1	55.25	54.86-55.55				data not available			96764	1.55	
	pk.11-17	1	56.26	55.83-56.45				data not available			92045	1.47	
	pk.11-18	1	57.25	56.72-58.03	1504.29	1503.93	$M+3H^{+}$	H5HN5F1NA5C-PA	◇4◆1		178884	2.86	
	pk.11-19	1	59.84	59.41-60.17	1044.24<br>1391.74	1043.92<br>1391.56	$M+4H^{+}$<br>$M+3H^{+}$	H4HN4F1NA5C-PA	◇3◆2	1002(H1HN1NA2)	124410	1.99	α2,3, α2,6NA LacNAc
	pk.11-20	1	62.81	62.51-63.13	1459.27			data not available			45212	0.72	
