# Supplementary material for: Toward robust N-glycomics of various tissue samples that may contain glycans with unknown or unexpected structures
Source: Sci Rep. 2021 Mar 18;11:6334. doi: 10.1038/s41598-021-84668-x (PMC7973440; doi:10.1038/s41598-021-84668-x)
Supplement: Supplementary file 2 — Supplementary Information 2. [file 41598_2021_84668_MOESM2_ESM.pdf]

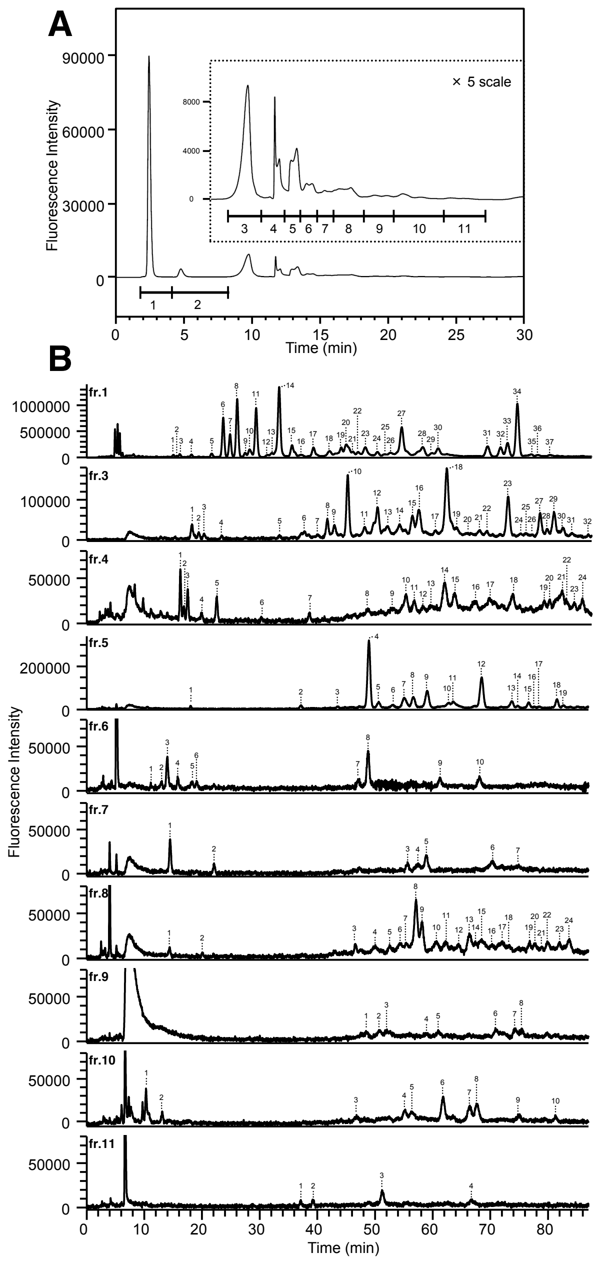

Figure S1. Elution profiles of PA-*N*-glycans from chicken colon. (A) PA-*N*-glycans from chicken colon were separated with a DEAE column. Each numbered major peak was fractionated and analyzed by LC-MS and MS/MS. (B) Elution profiles of PA-*N*-glycans from chicken colon on reversed-phase LC. Each fraction from the DEAE column was analyzed. The profile of fr. 2 from the DEAE column is not shown, because no obvious signals of PA-glycans were detected. Most of the detectable peaks eluted in 10–87 min were numbered, regardless of whether they contained PA-*N*-glycans. The results of LC-MS and MS/MS are summarized in Supplementary Table S1.

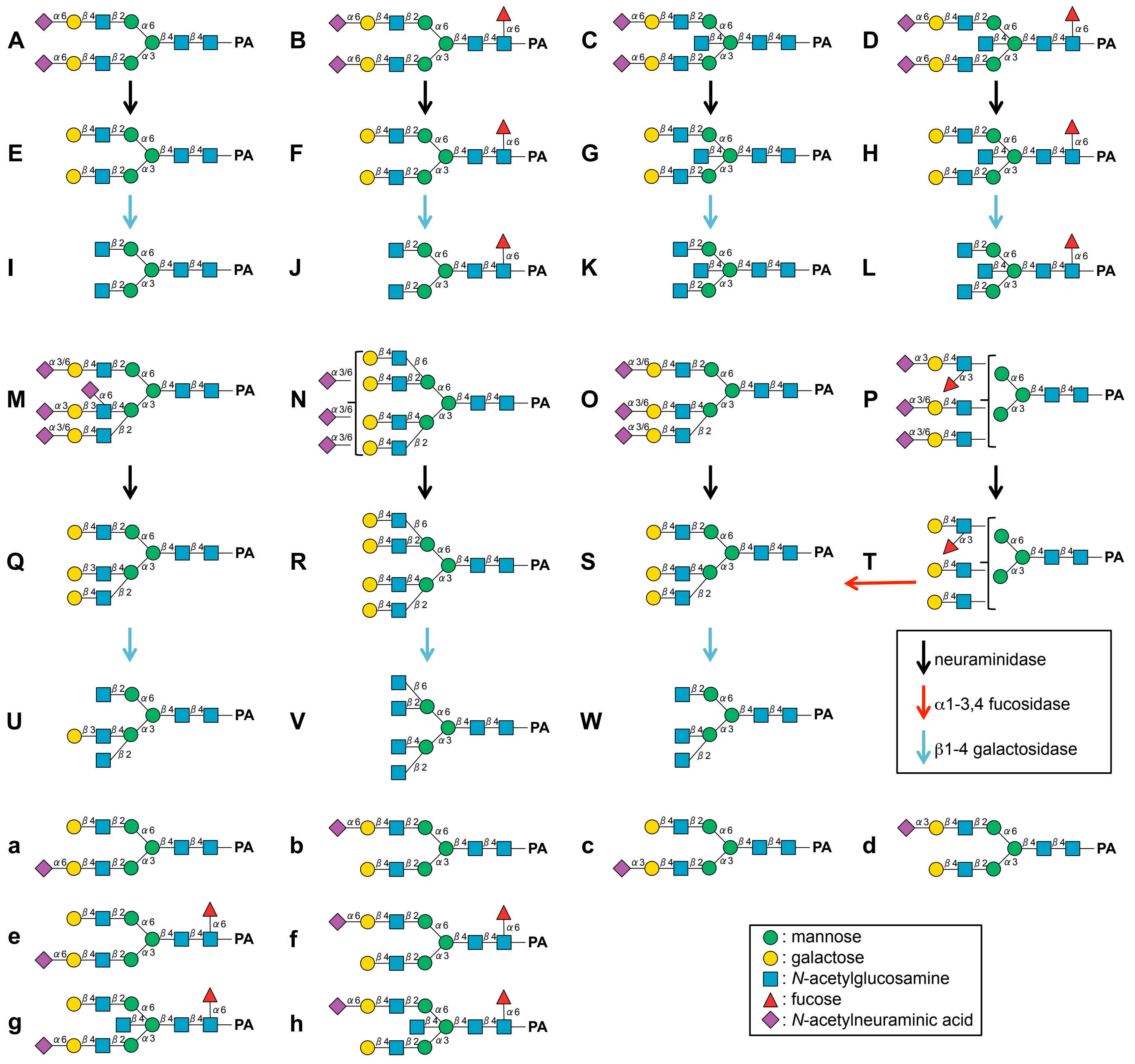

Figure S2. Structures of standard PA-*N*-glycans used for reversed-phase LC-MS and MS/MS. Sialylated PA-*N*-glycans were prepared from human γ-globulin (A, B, C, D), bovine fetuin (M), and α1-AGP (N, O, P), and sequentially digested with neuraminidase, α1-3,4 fucosidase (for T), and β1-4 galactosidase. α2,6-Monosialylated PA-*N*-glycans were prepared from human transferrin (a, b) and human γ-globulin (e, f, g, h). α2,3-Monosialylated PA-*N*-glycans (c, d) were prepared by treatment of asialo-biantennary PA-*N*-glycans with recombinant α2,3-sialyltransferase from *Photobacterium phosphoreum*. The standard Symbol Nomenclature for Glycan system was used for monosaccharide symbols.

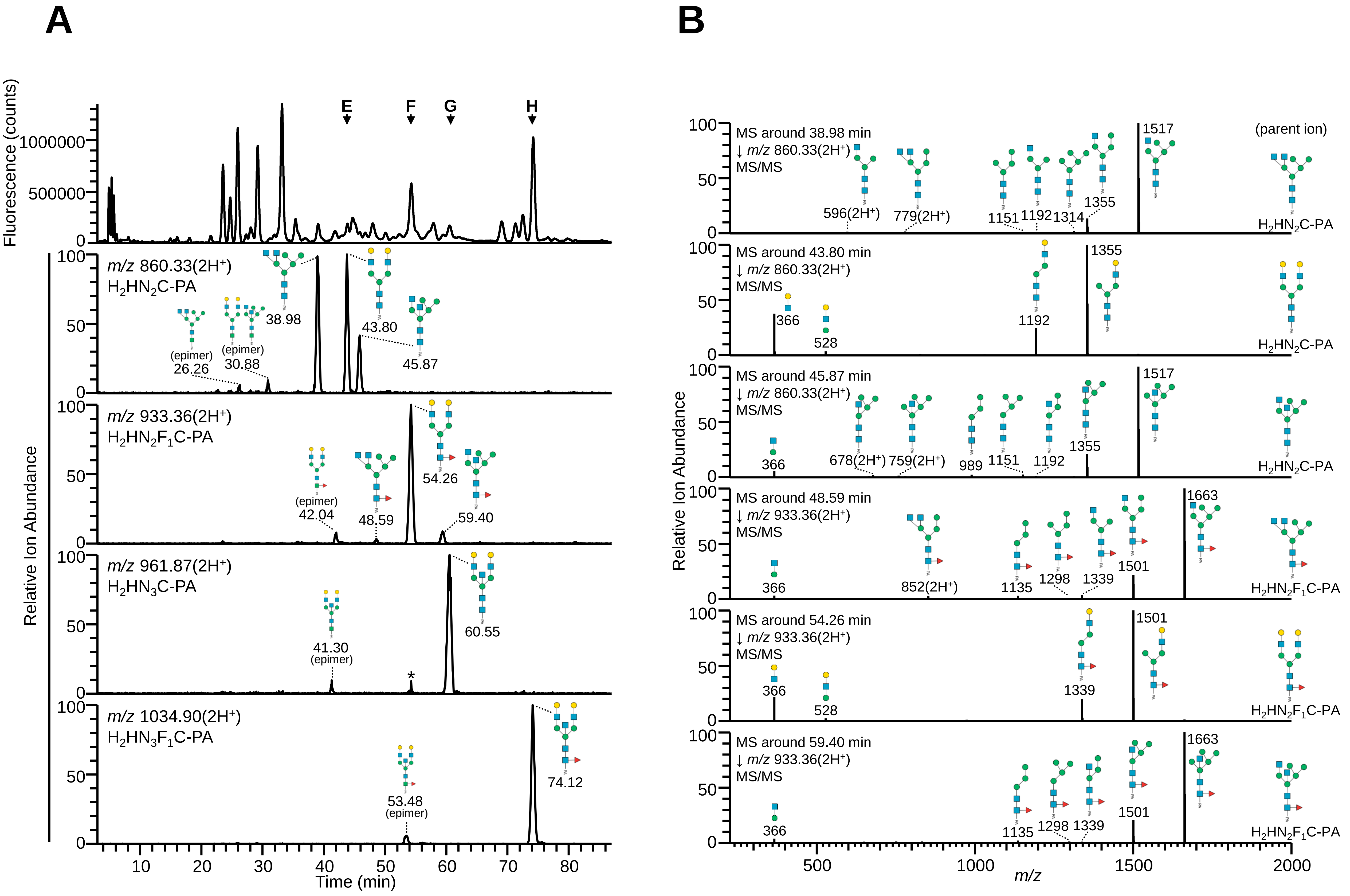

Figure S3-1. Different elution positions among glycan isomers on reversed-phase LC. (A) EICs at *m/z* 860.33, 933.36, 961.87, and 1034.90 of neutral PA-*N*-glycans (fr. 1 from DEAE column) from chicken colon. Arrows with alphabetical characters indicate the elution positions of the standard PA-*N*-glycans (Supplementary Figure S2). The peaks indicated by an asterisk (*) are probably artificial ion signals derived from large amounts of PA-*N*-glycans eluted around the corresponding times. (B) Comparison of MS/MS spectra of glycan isomers at *m/z* 860.33 and 933.36 eluted at different times, as shown in Supplementary Figure S3-1A. The structures of fragments shown in the figures are representative examples, and other isomeric ions can also be generated.

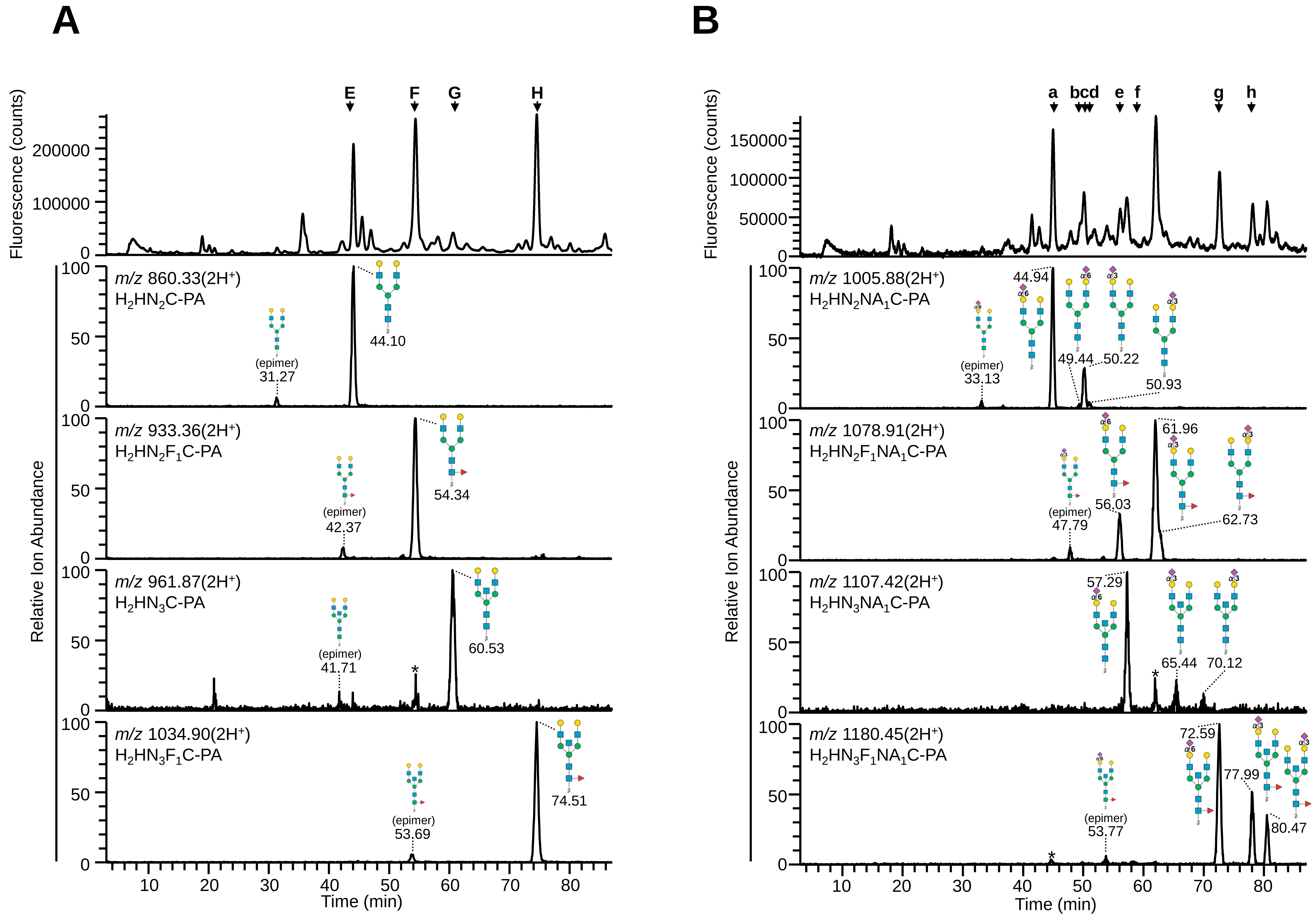

Figure S3-2. Different elution positions among glycan isomers on reversed-phase LC. (A) EICs at *m/z* 860.33, 933.36, 961.87, and 1034.90 of biantennary PA-*N*-glycans in neuraminidase/α1-3,4 fucosidase-treated fr. 3 from chicken colon or human γ-globulin. Arrows with alphabetical characters indicate the elution positions of the standard PA-*N*-glycans (Supplementary Figure S2). The peaks indicated by an asterisk (*) are probably artificial ion signals derived from large amounts of PA-*N*-glycans eluted around the corresponding times. (B) EICs at *m/z* 1005.88, 1078.91, 1107.42, and 1180.45 of monosialylated biantennary PA-*N*-glycans in fr. 3. (C, D: see next page.)

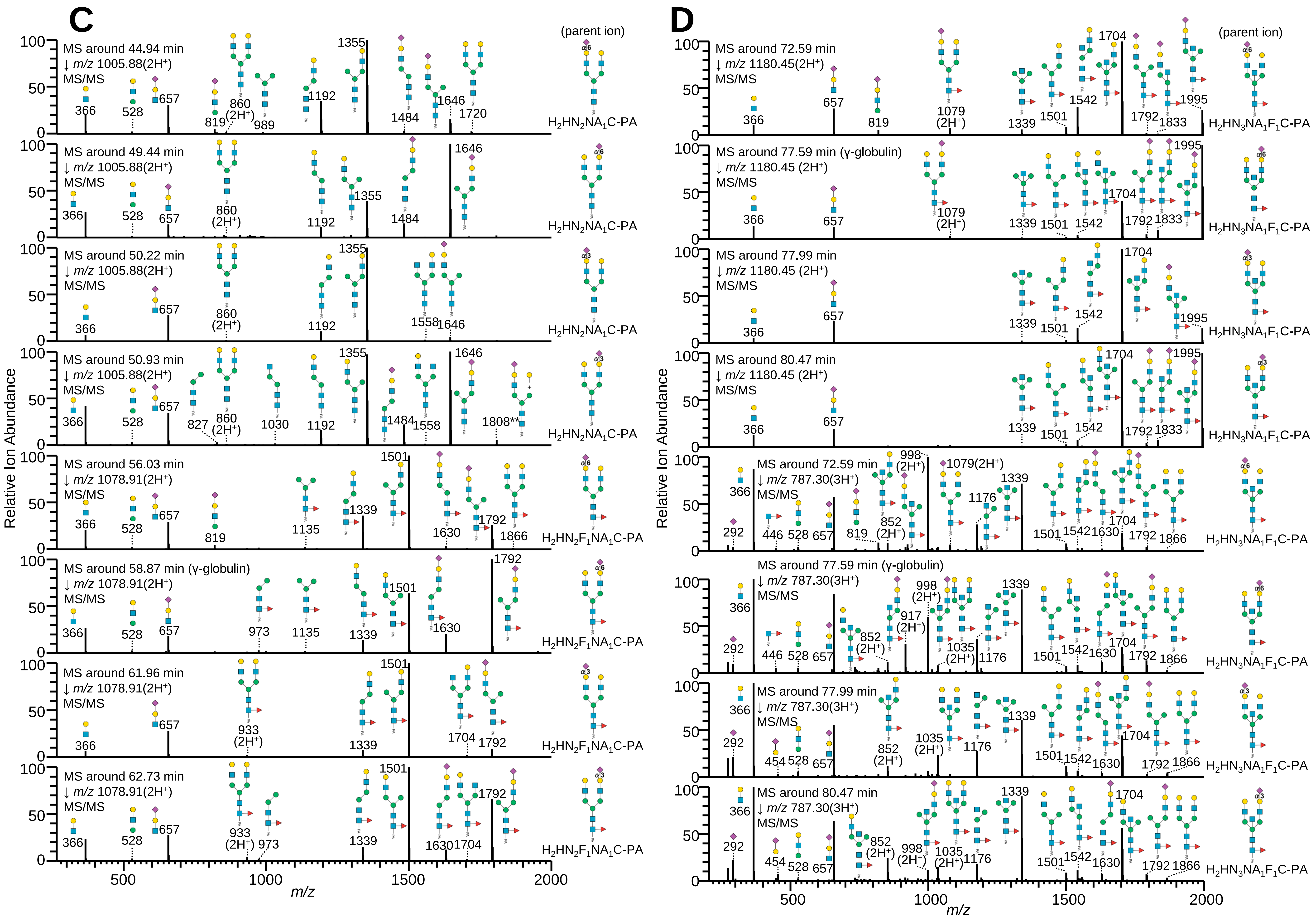

Figure S3-2. (Continued.) (C) Comparison of MS/MS spectra of glycan isomers at *m/z* 1005.88 and 1078.91 eluted at different times, as shown in Supplementary Figure S3-2B. MS/MS data at *m/z* 1078.91 around 58.87 min is from Standard f (Supplementary Figure S2) from human γ-globulin. The structures of fragments shown in the figures are representative examples, and other isomeric ions can also be generated. The peaks indicated by double asterisks (**) are probably artificial ion signals generated by ion rearrangements. (D) Comparison of MS/MS spectra of glycan isomers at *m/z* 1180.45 ($2H^+$) and 787.30 ($3H^+$) eluted at different times, as shown in Supplementary Figure S3-2B. MS/MS data around 77.59 min are from Standard h (Supplementary Figure S2) from human γ-globulin.

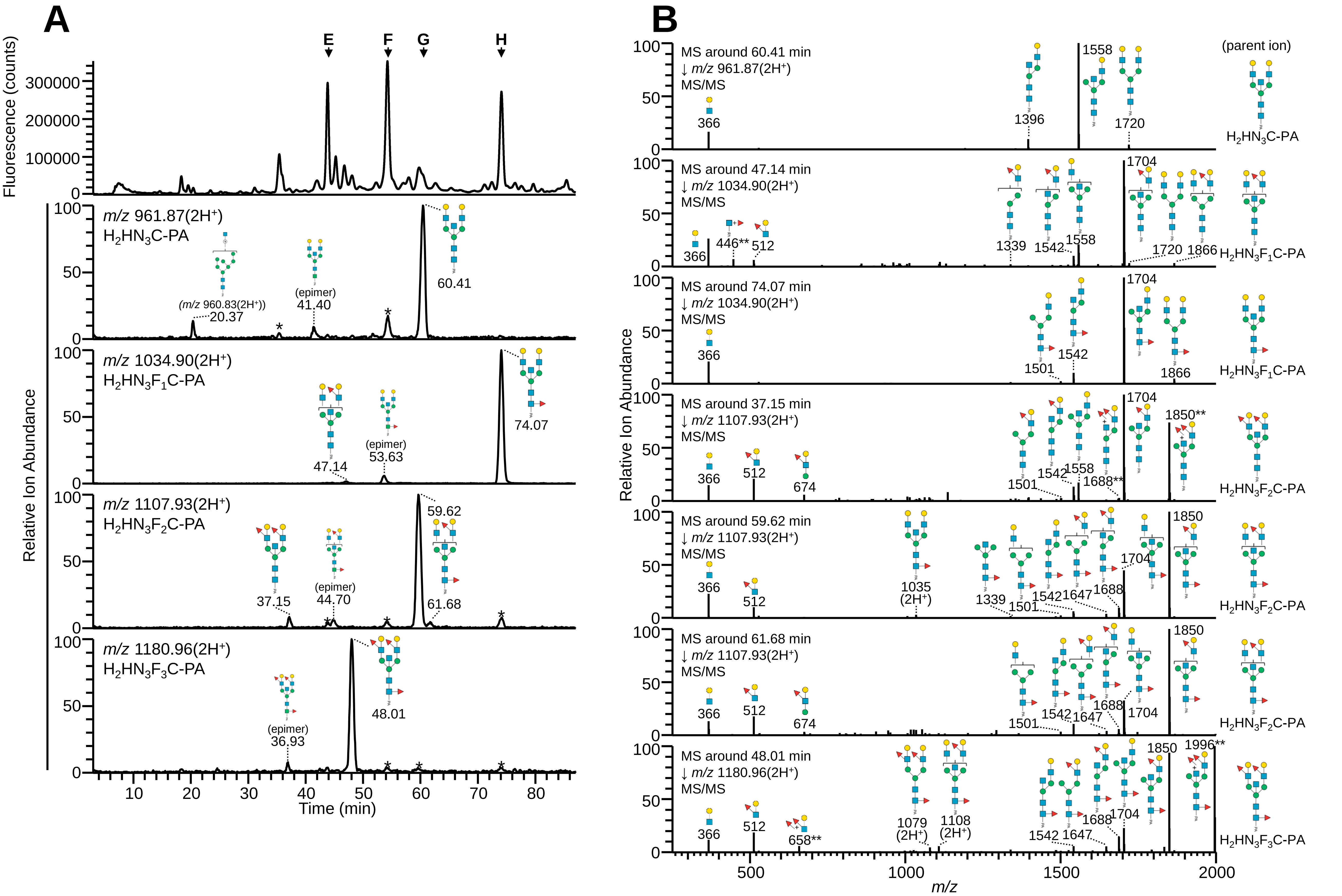

Figure S3-3. Different elution positions among glycan isomers with or without multiple fucosylation on reversed-phase LC. (A) EICs at *m/z* 961.87, 1034.90, 1107.93, and 1180.96 of PA-*N*-glycans in neuraminidase-treated fr. 3. Arrows with alphabetical characters indicate the elution positions of the standard PA-*N*-glycans (Supplementary Figure S2). The peaks indicated by an asterisk (*) are probably artificial ion signals derived from large amounts of PA-*N*-glycans eluted around the corresponding times. (B) Comparison of MS/MS spectra of glycan isomers at *m/z* 961.87, 1034.90, 1107.93, and 1180.96 eluted at different times, as shown in Supplementary Figure S3-3A. The structures of fragments shown in the figures are representative examples, and other isomeric ions can also be generated. The peaks indicated by double asterisks (**) are probably artificial ion signals generated by ion rearrangements.

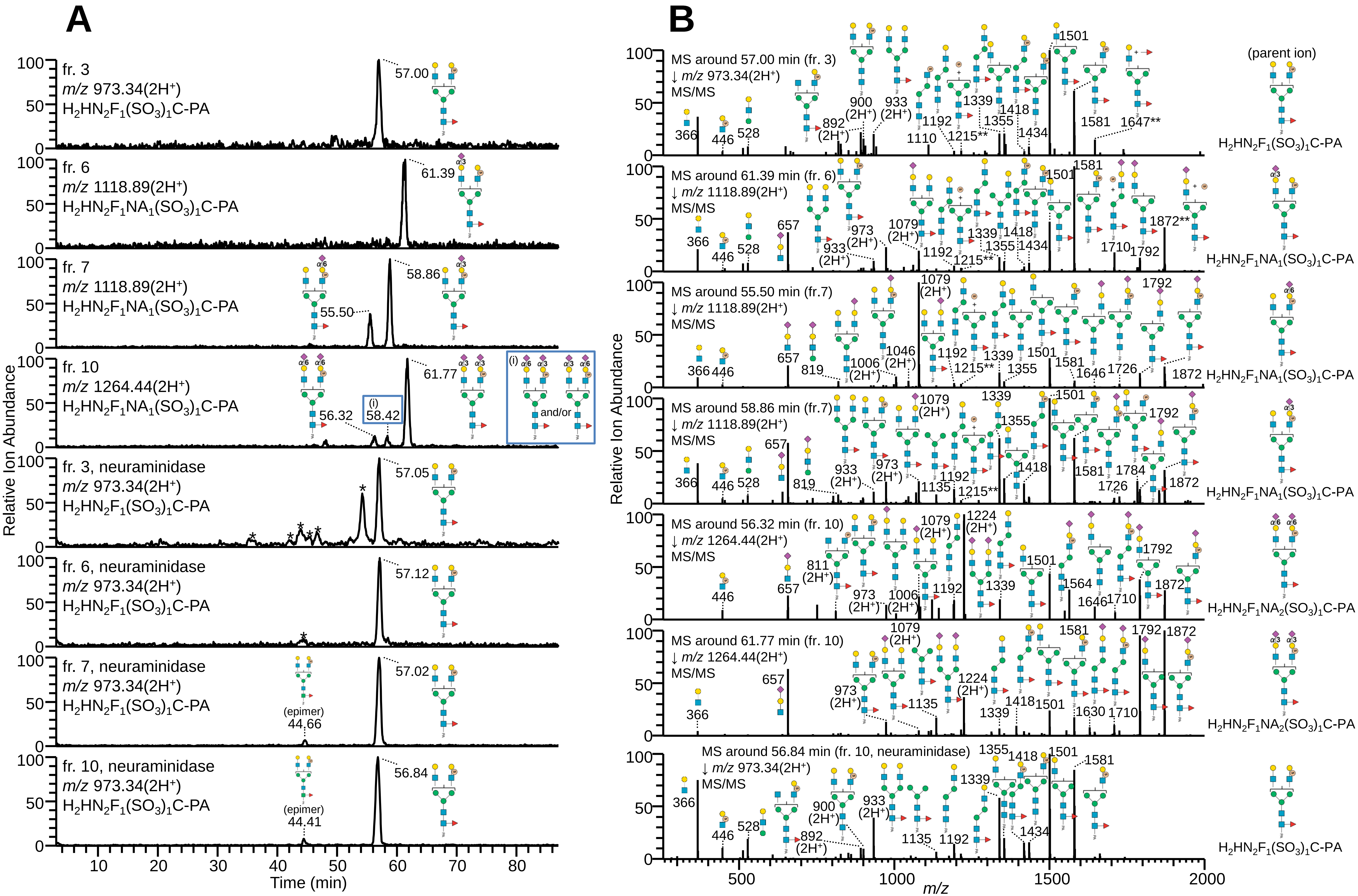

Figure S3-4. Different elution positions among glycan isomers with a sulfate group on reversed-phase LC. (A) EICs at *m/z* 973.34, 1118.89, and 1264.44 of PA-*N*-glycans in fr. 3, 6, 7, and 10 with or without neuraminidase treatments. The peaks indicated by an asterisk (*) are probably artificial ion signals derived from large amounts of PA-*N*-glycans eluted around the corresponding times. Positions and linkages of NeuAc were determined by SALSA and permethylation (Figure 6). (B) Comparison of MS/MS spectra of glycan isomers at *m/z* 973.34 (fr. 3, neuraminidase-treated fr. 10), 1118.89 (fr. 6, 7), and 1264.44 (fr. 10) eluted at different times, as shown in Supplementary Figure S3-4A. The structures of fragments shown in the figures are representative examples, and other isomeric ions can also be generated. The peaks indicated by double asterisks (**) are probably artificial ion signals generated by ion rearrangements.

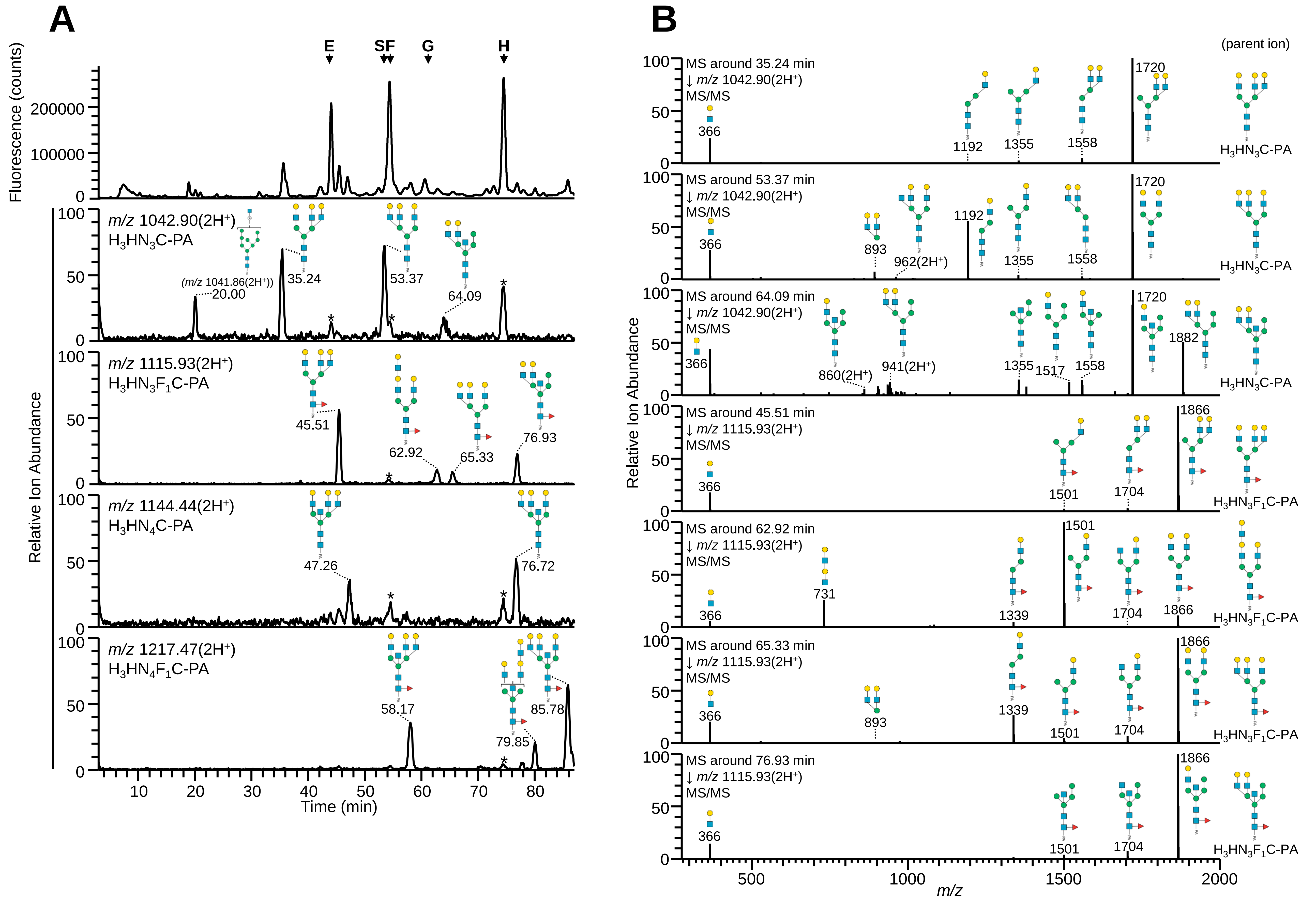

Figure S3-5. Different elution positions among glycan isomers on reversed-phase LC. (A) EICs at *m/z* 1042.90, 1115.93, 1144.44, and 1217.47 of triantennary PA-*N*-glycans in neuraminidase/α1-3,4 fucosidase-treated fr. 3. Arrows with alphabetical characters indicate the elution positions of the standard PA-*N*-glycans (Supplementary Figure S2). The peaks indicated by an asterisk (*) are probably artificial ion signals derived from large amounts of PA-*N*-glycans eluted around the corresponding times. (B) Comparison of MS/MS spectra of glycan isomers at *m/z* 1042.90 and 1115.93 eluted at different times, as shown in Supplementary Figure S3-5A. The structures of fragments shown in the figures are representative examples, and other isomeric ions can also be generated.

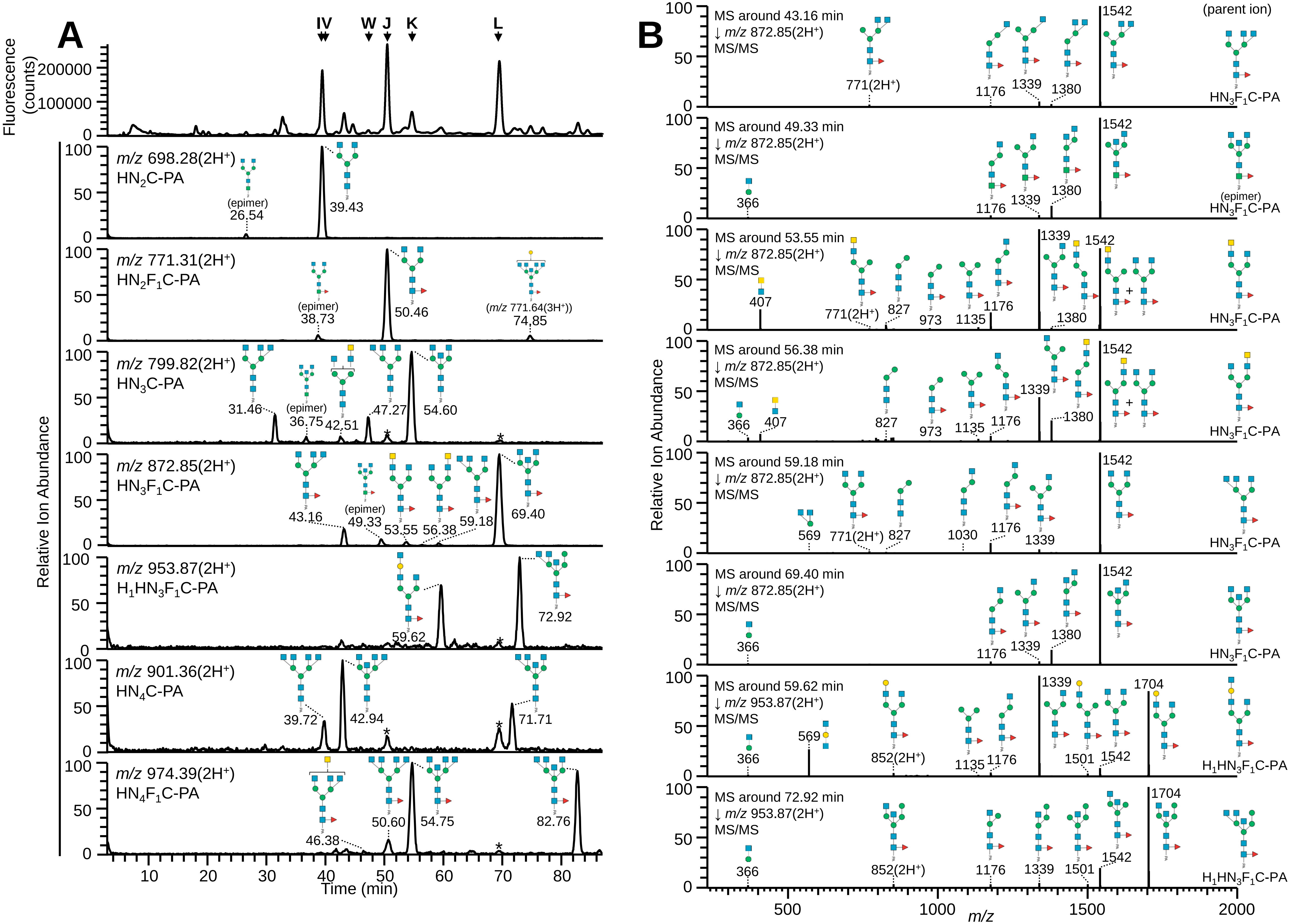

Figure S3-6. Different elution positions among glycan isomers on reversed-phase LC. (A) EICs at *m/z* 698.28, 771.31, 799.82, 872.85, 953.87, 901.36, and 974.39 of PA-*N*-glycans in neuraminidase/α1-3,4 fucosidase/β1-4 galactosidase-treated fr. 3. Arrows with alphabetical characters indicate the elution positions of the standard PA-*N*-glycans (Supplementary Figure S2). The peaks indicated by an asterisk (*) are probably artificial ion signals derived from large amounts of PA-*N*-glycans eluted around the corresponding times. (B) Comparison of MS/MS spectra of glycan isomers at *m/z* 872.85 and 953.87 of PA-*N*-glycans eluted at different times, as shown in Supplementary Figure S3-6A. The structures of fragments shown in the figures are representative examples, and other isomeric ions can also be generated.

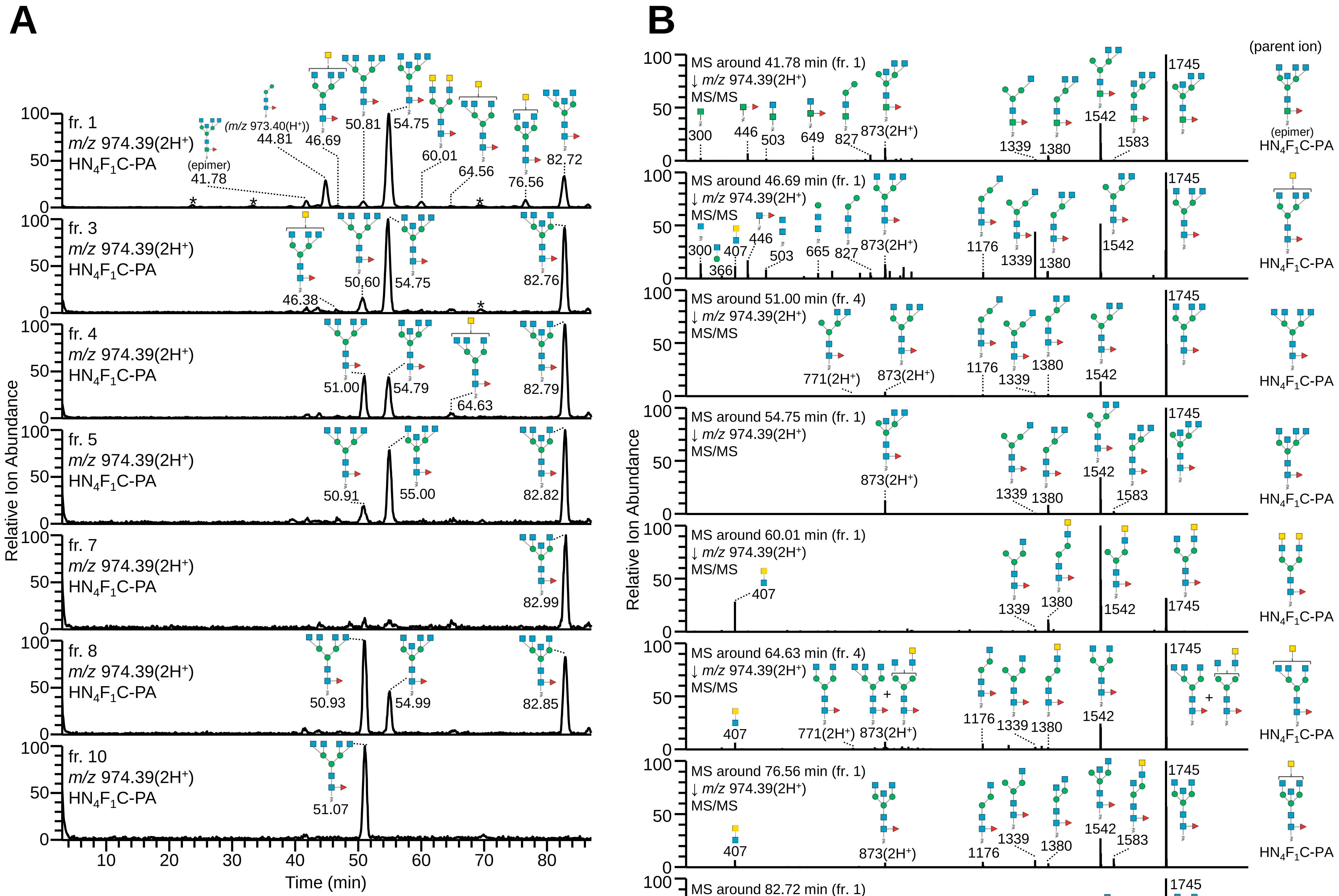

Figure S3-7. Different elution positions among glycan isomers on reversed-phase LC. (A) EICs at *m/z* 974.39 of PA-*N*-glycans in neuraminidase/α1-3,4 fucosidase/β1-4 galactosidase-treated fr. 1, 3, 4, 5, 7, 8, and 10. The peaks indicated by an asterisk (*) are probably artificial ion signals derived from large amounts of PA-*N*-glycans eluted around the corresponding times. (B) Comparison of MS/MS spectra of glycan isomers at *m/z* 974.39 of PA-*N*-glycans (fr. 1 or fr. 4) eluted at different times, as shown in Supplementary Figure S3-7A. The structures of fragments shown in the figures are representative examples, and other isomeric ions can also be generated.

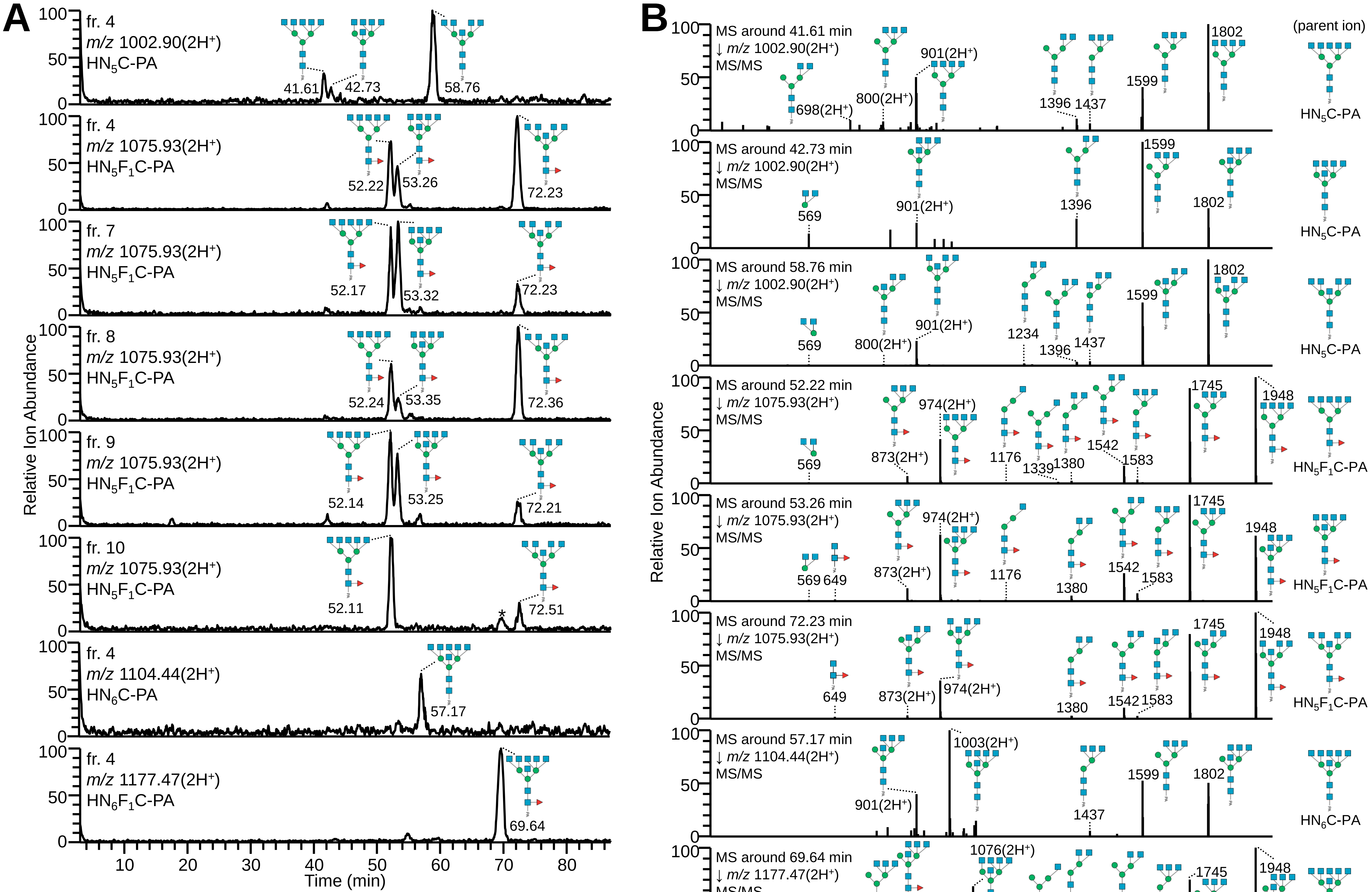

Figure S3-8. Different elution positions among glycan isomers on reversed-phase LC. (A) EICs at *m/z* 1002.90, 1075.93, 1104.44, and 1177.47 of PA-*N*-glycans in neuraminidase/α1-3,4 fucosidase/β1-4 galactosidase-treated fr. 4, and 7–10. The peaks indicated by an asterisk (*) are probably artificial ion signals derived from large amounts of PA-*N*-glycans eluted around the corresponding times. (B) Comparison of MS/MS spectra of glycan isomers at *m/z* 1002.90, 1075.93, 1104.44, and 1177.47 of PA-*N*-glycans (fr. 4) eluted at different times, as shown in Supplementary Figure S3-8A. The structures of fragments shown in the figures are representative examples, and other isomeric ions can also be generated.

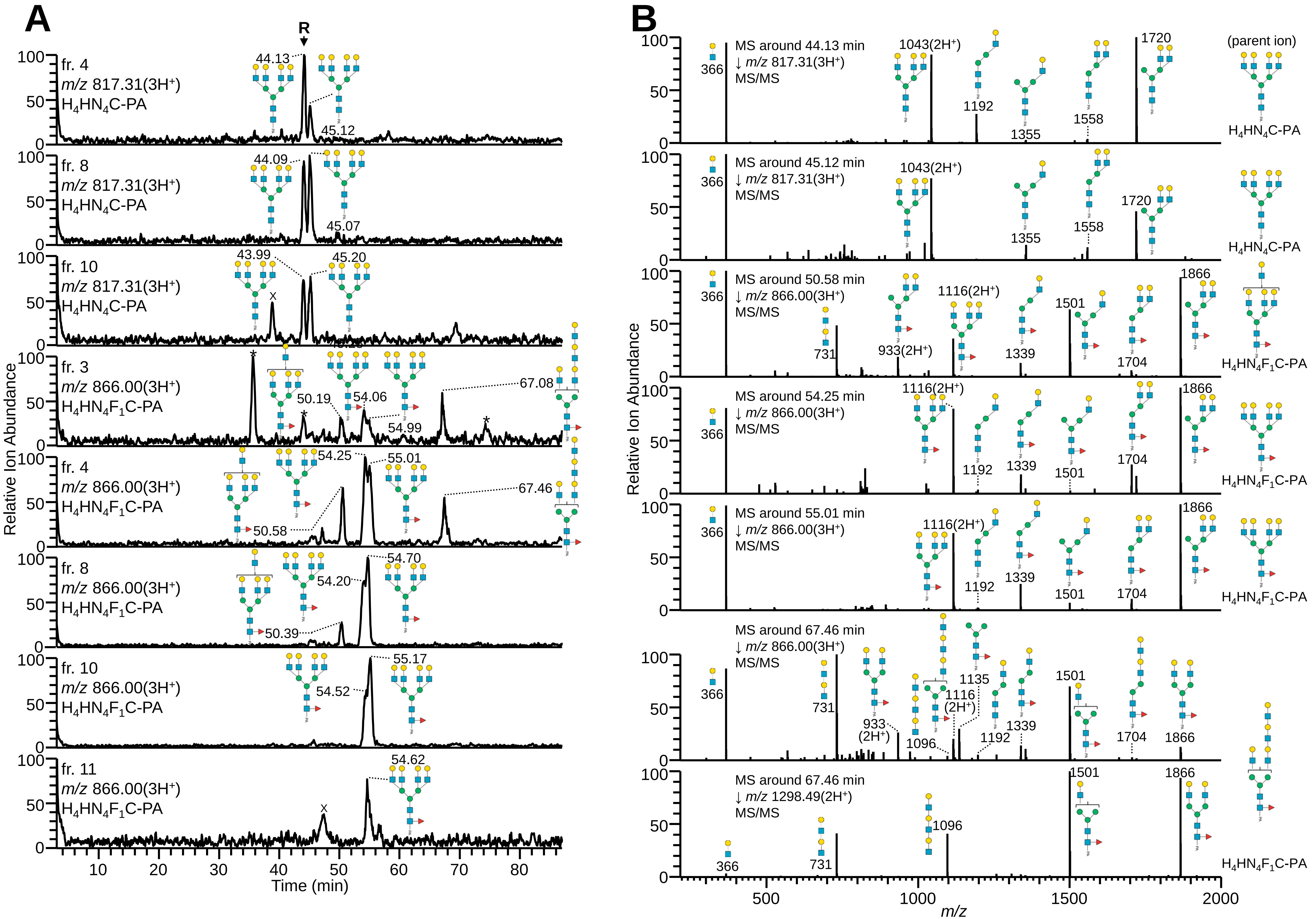

Figure S3-9. Different elution positions among glycan isomers on reversed-phase LC. (A) EICs at *m/z* 817.31 and 866.00 of PA-*N*-glycans in neuraminidase/α1-3,4 fucosidase-treated fr. 3, 4, 8, 10, and 11. An arrow indicates the elution positions of the standard PA-*N*-glycan R (Supplementary Figure S1). The peaks indicated by an asterisk (*) are probably artificial ion signals derived from large amounts of PA-*N*-glycans eluted around the corresponding times. The peaks indicated by an X are derived from non-glycan contaminants. (B) Comparison of MS/MS spectra of glycan isomers at *m/z* 817.31, 866.00 ($3H^+$), and 1298.49 ($2H^+$) of PA-*N*-glycans (fr. 4) eluted at different times, as shown in Supplementary Figure S3-9A. The structures of fragments shown in the figures are representative examples, and other isomeric ions can also be generated.

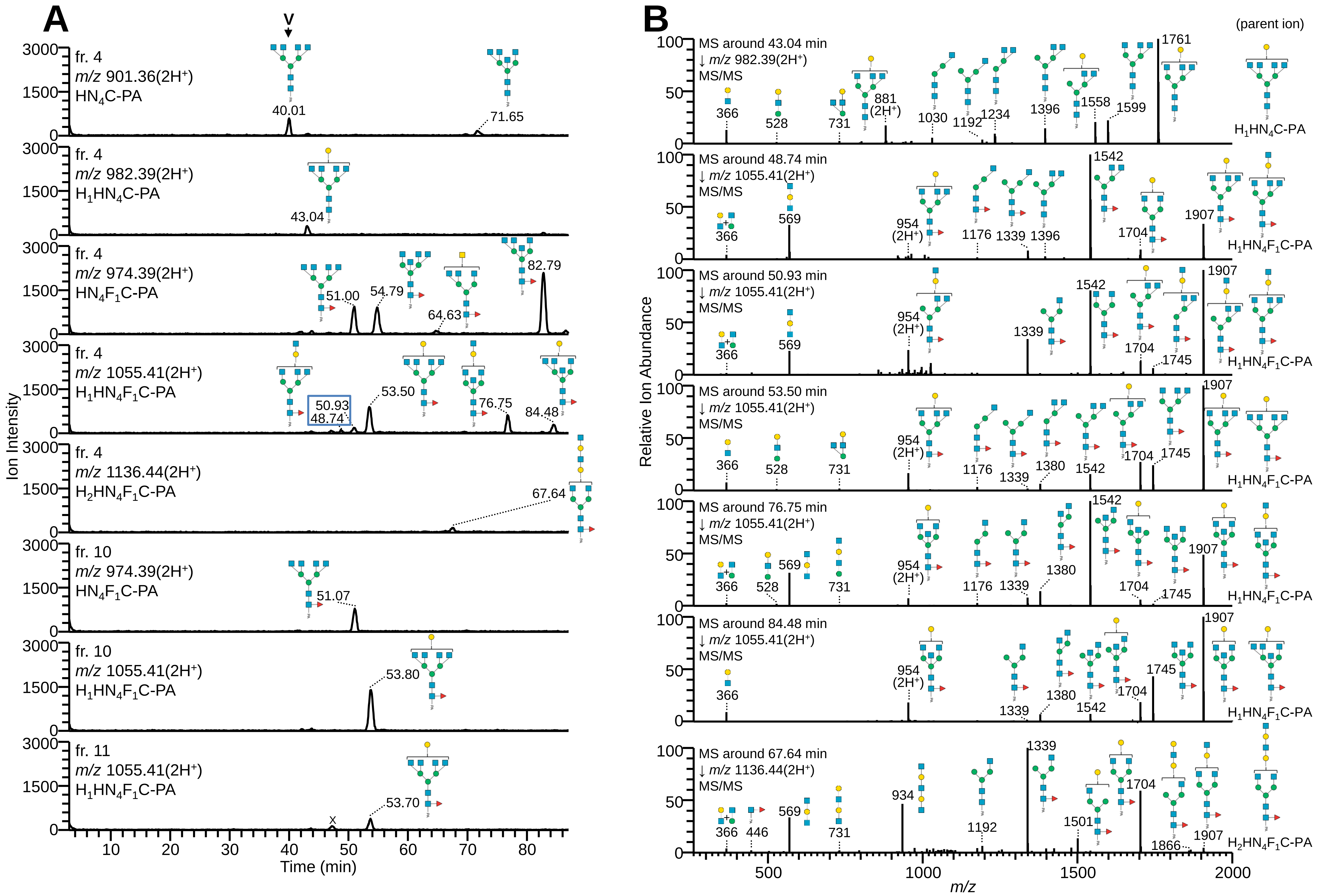

Figure S3-10. Different elution positions among glycan isomers on reversed-phase LC. (A) EICs at *m/z* 901.36, 982.39, 974.39, 1055.41, and 1136.44 of PA-*N*-glycans in neuraminidase/α1-3,4 fucosidase/β1-4 galactosidase-treated fr. 4, 10, and 11. An arrow indicates the elution positions of the standard PA-*N*-glycan V (Supplementary Figure S2). The peaks indicated by an X are derived from non-glycan contaminants. The *y*-axis represents absolute ion intensity to allow inter-spectrum comparison of the ion signals actually detected. (B) Comparison of MS/MS spectra of glycan isomers at *m/z* 982.39, 1055.41, and 1136.44 of PA-*N*-glycans (fr. 4) eluted at different times, as shown in Supplementary Figure S3-10A. The structures of fragments shown in the figures are representative examples, and other isomeric ions can also be generated.

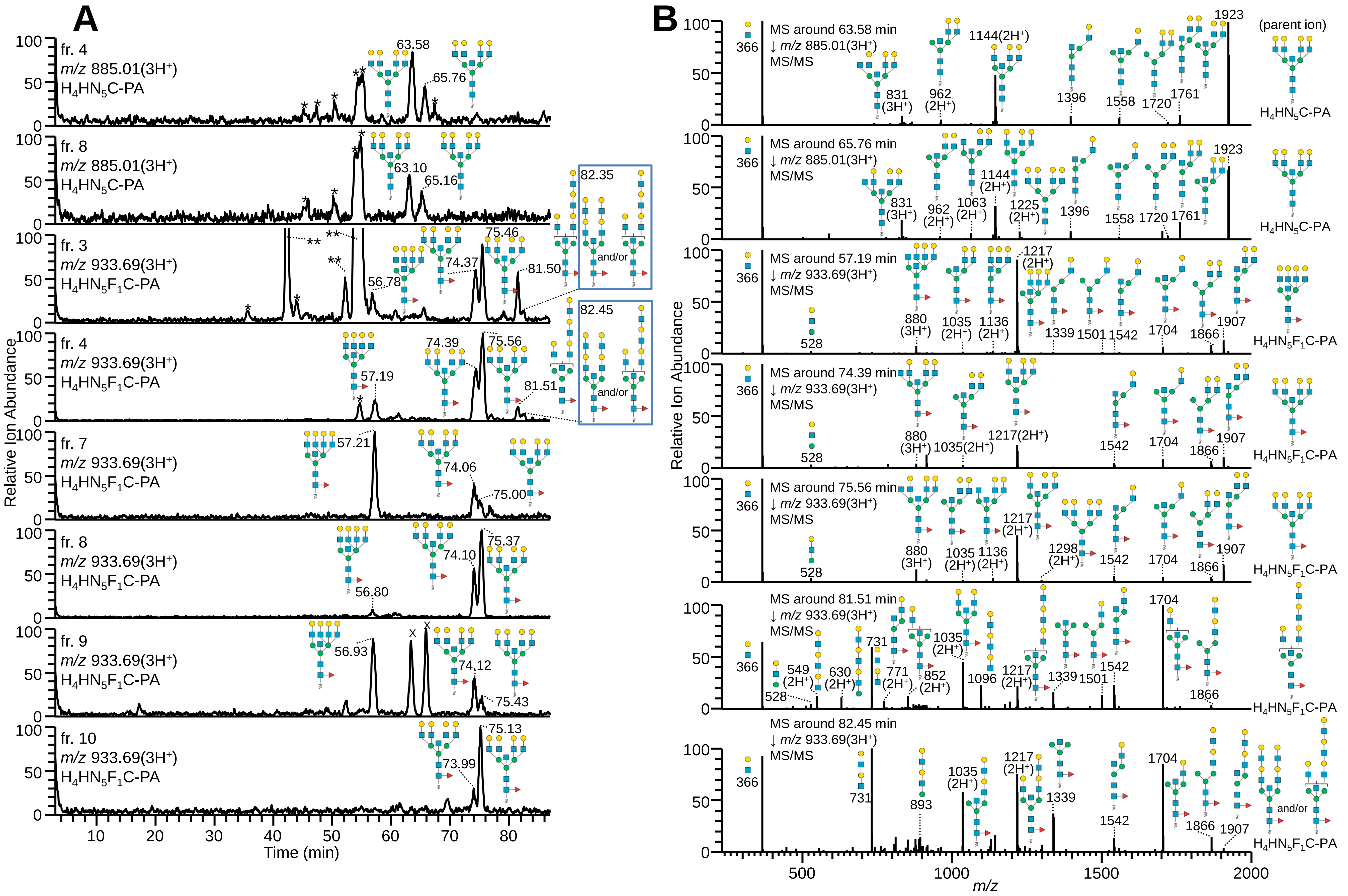

Figure S3-11. Different elution positions among glycan isomers on reversed-phase LC. (A) EICs at *m/z* 885.01 and 933.69 of PA-*N*-glycans in neuraminidase/α1-3,4 fucosidase-treated fr. 3, 4, and 7–10. The peaks indicated by an asterisk (*) are probably artificial ion signals derived from large amounts of PA-*N*-glycans eluted around the corresponding times. The peaks indicated by double asterisks (**) are signals derived from PA-*N*-glycans at *m/z* 933.36 [$Hex_2HexNAc_2Fuc_1C$-PA($2H^+$)] eluted around the corresponding times. The peaks indicated by an X are derived from non-glycan contaminants. (B) Comparison of MS/MS spectra of glycan isomers at *m/z* 885.01 and 933.69 of PA-*N*-glycans (fr. 4) eluted at different times, as shown in Supplementary Figure S3-11A. The structures of fragments shown in the figures are representative examples, and other isomeric ions can also be generated.

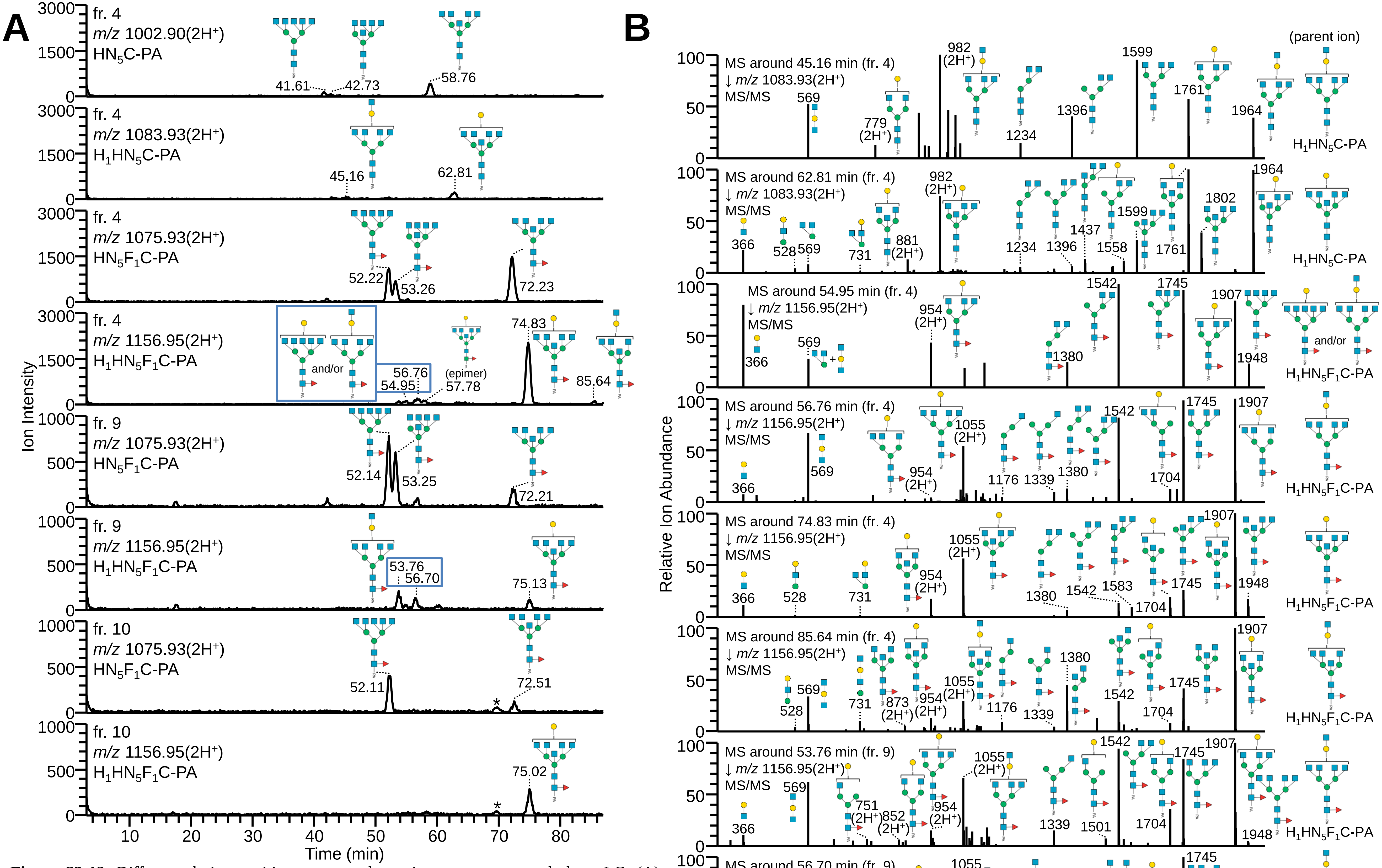

Figure S3-12. Different elution positions among glycan isomers on reversed-phase LC. (A) EICs at *m/z* 1002.90, 1083.93, 1075.93, and 1156.95 of PA-*N*-glycans in neuraminidase/α1-3,4 fucosidase/β1-4 galactosidase-treated fr. 4, 9, and 10. The peaks indicated by an asterisk (*) are probably artificial ion signals derived from large amounts of PA-*N*-glycans eluted around the corresponding times. The *y*-axis represents absolute ion intensity to allow inter-spectrum comparison of the ion signals actually detected. (B) Comparison of MS/MS spectra of glycan isomers at *m/z* 1083.93 and 1156.95 of PA-*N*-glycans (fr. 4 and 9) eluted at different times, as shown in Supplementary Figure S3-12A. The structures of fragments shown in the figures are representative examples, and other isomeric ions can also be generated.

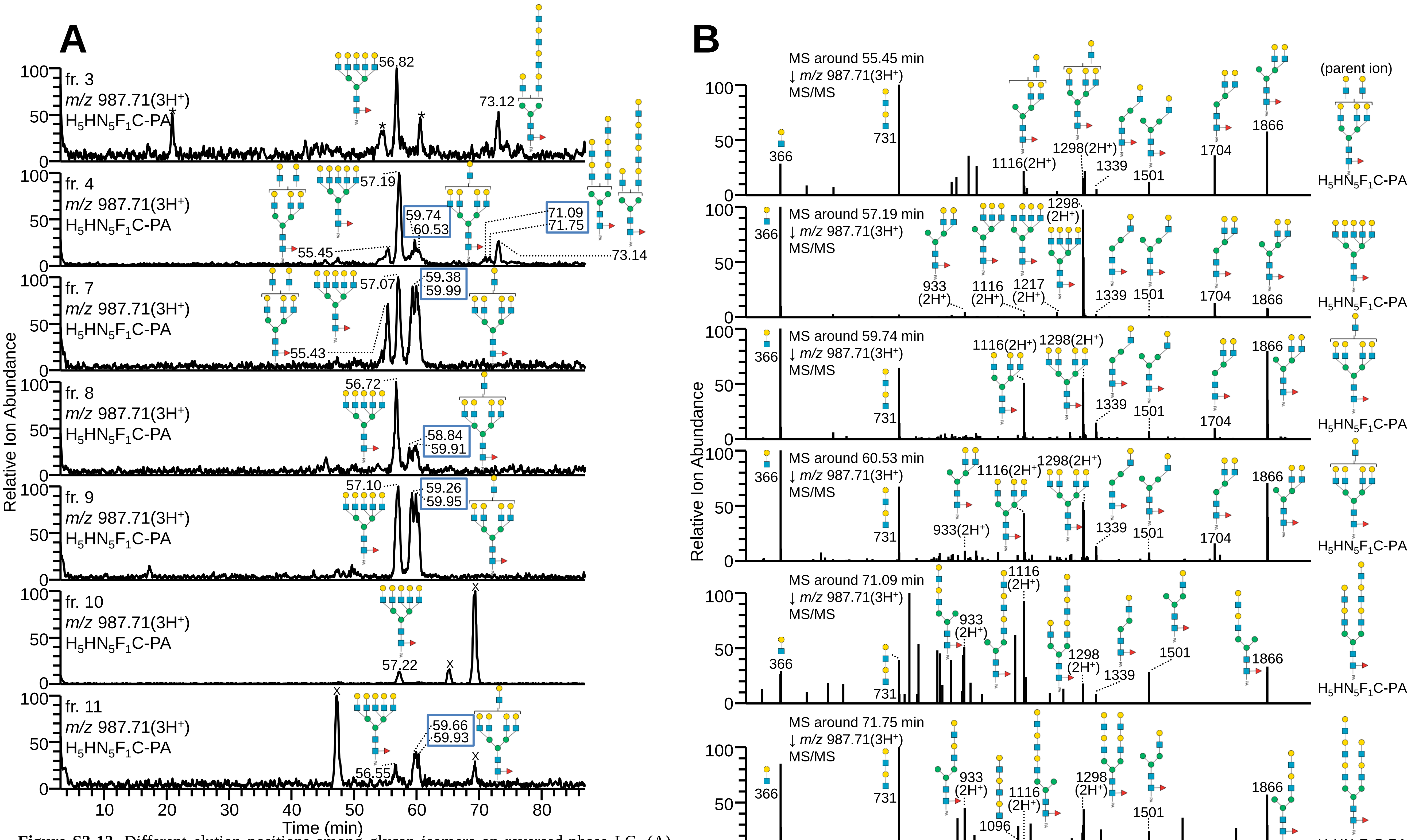

Figure S3-13. Different elution positions among glycan isomers on reversed-phase LC. (A) EICs at *m/z* 987.71 of PA-*N*-glycans in neuraminidase/α1-3,4 fucosidase-treated fr. 3, 4, and 7–11. The peaks indicated by an asterisk (*) are probably artificial ion signals derived from large amounts of PA-*N*-glycans eluted around the corresponding times. The peaks indicated by an X are derived from non-glycan contaminants. (B) Comparison of MS/MS spectra of glycan isomers at *m/z* 987.71 of PA-*N*-glycans (fr. 4) eluted at different times, as shown in Supplementary Figure S3-13A. The structures of fragments shown in the figures are representative examples, and other isomeric ions can also be generated. The MS and MS/MS signals at *m/z* 987.71 of the PA-*N*-glycans that eluted around 71.09 and 71.75 min were very weak, and many noise signals that could not be properly assigned were detected. Although the structural assignments remained ambiguous, some hallmark B ion signals, such as 731 and 1096, indicated the presence of LacNAc repeats.

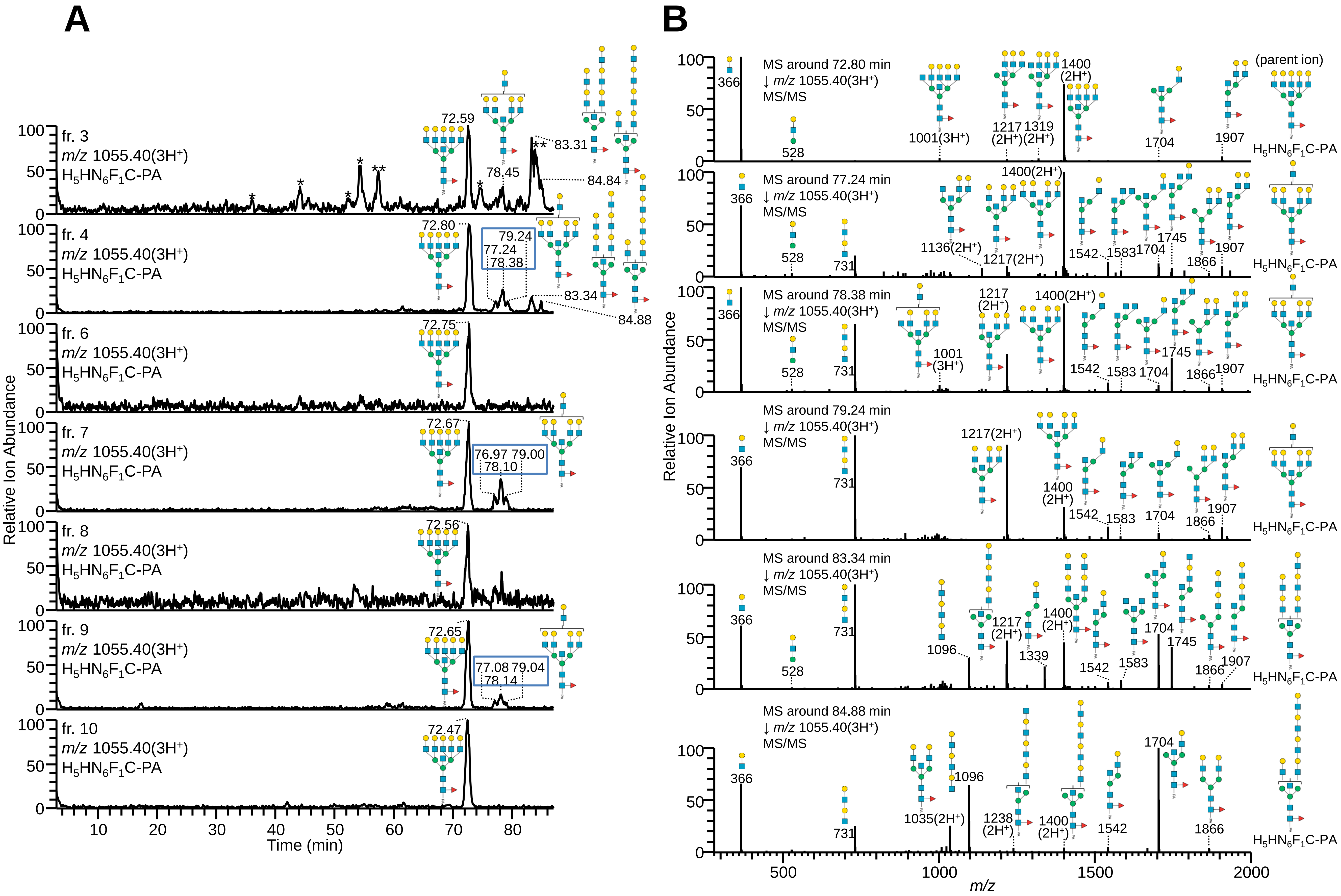

Figure S3-14. Different elution positions among glycan isomers on reversed-phase LC. (A) EICs at *m/z* 1055.40 of PA-*N*-glycans in neuraminidase/α1-3,4 fucosidase-treated fr. 3, 4, and 6–10. The peaks indicated by an asterisk (*) are probably artificial ion signals derived from large amounts of PA-*N*-glycans eluted around the corresponding times. The peaks indicated by double asterisks (**) are signals derived from PA-*N*-glycans at *m/z* 1055.41 [$Hex_1HexNAc_4Fuc_1$C-PA(2H$^+$)] eluted around the corresponding times. (B) Comparison of MS/MS spectra of glycan isomers at *m/z* 1055.40 of PA-*N*-glycans (fr. 4) eluted at different times, as shown in Supplementary Figure S3-14A. The structures of fragments shown in the figures are representative examples, and other isomeric ions can also be generated.

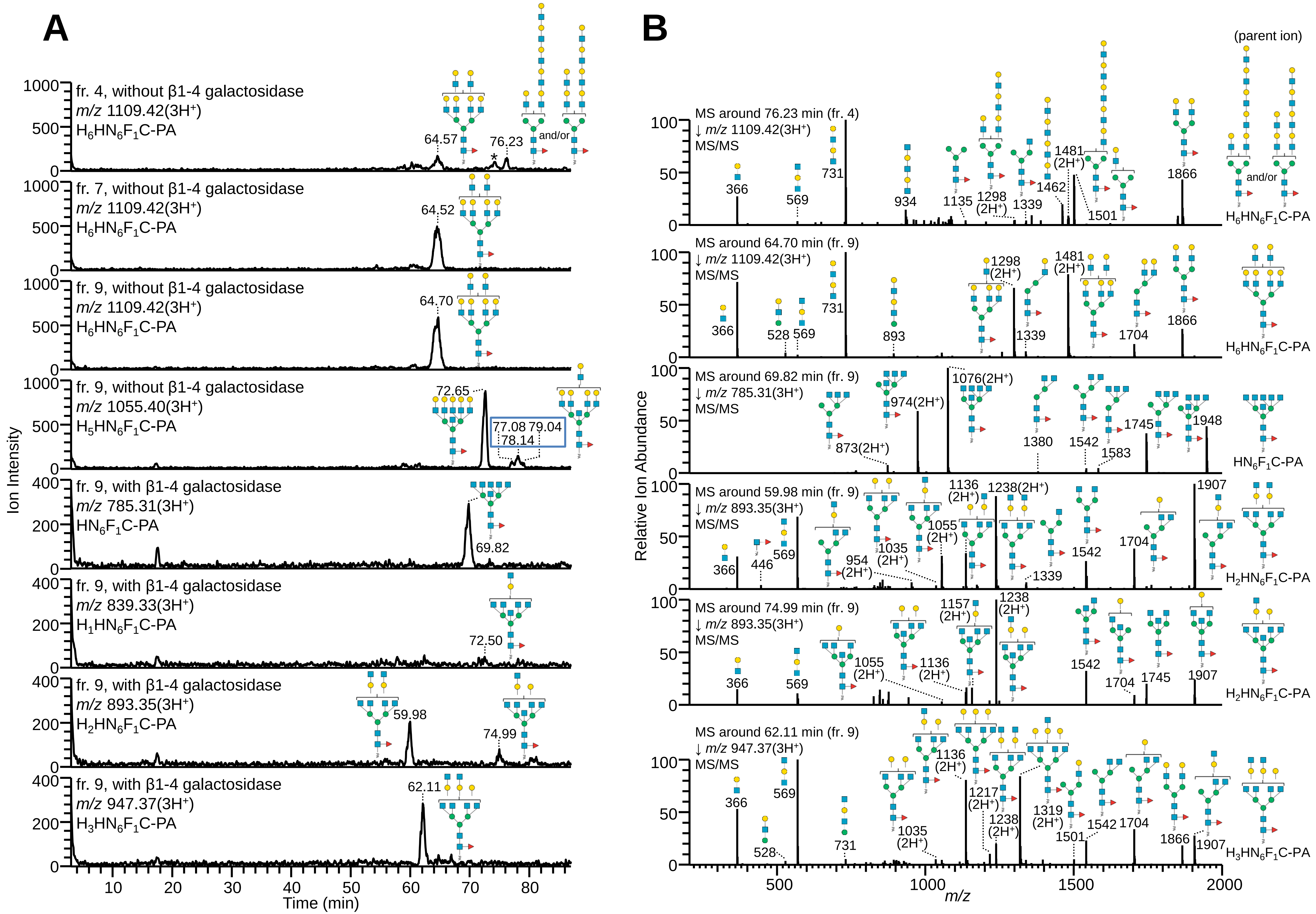

Figure S3-15. Different elution positions among glycan isomers on reversed-phase LC. (A) EICs at *m/z* 1109.42 and 1055.40 of PA-*N*-glycans in neuraminidase/α1-3,4 fucosidase-treated fr. 4, 7, and 9, and EICs at *m/z* 785.31, 839.33, 893.35, and 947.37 of PA-*N*-glycans in neuraminidase/α1-3,4 fucosidase/β1-4 galactosidase-treated fr. 9. The peaks indicated by an asterisk (*) are probably artificial ion signals derived from large amounts of PA-*N*-glycans eluted around the corresponding times. The *y*-axis represents absolute ion intensity to allow inter-spectrum comparison of the ion signals actually detected. (B) Comparison of MS/MS spectra of glycan isomers at *m/z* 1109.42, 785.31, 893.35, and 947.37 of PA-*N*-glycans (fr. 4 and 9) eluted at different times, as shown in Supplementary Figure S3-15A. The structures of fragments shown in the figures are representative examples, and other isomeric ions can also be generated.

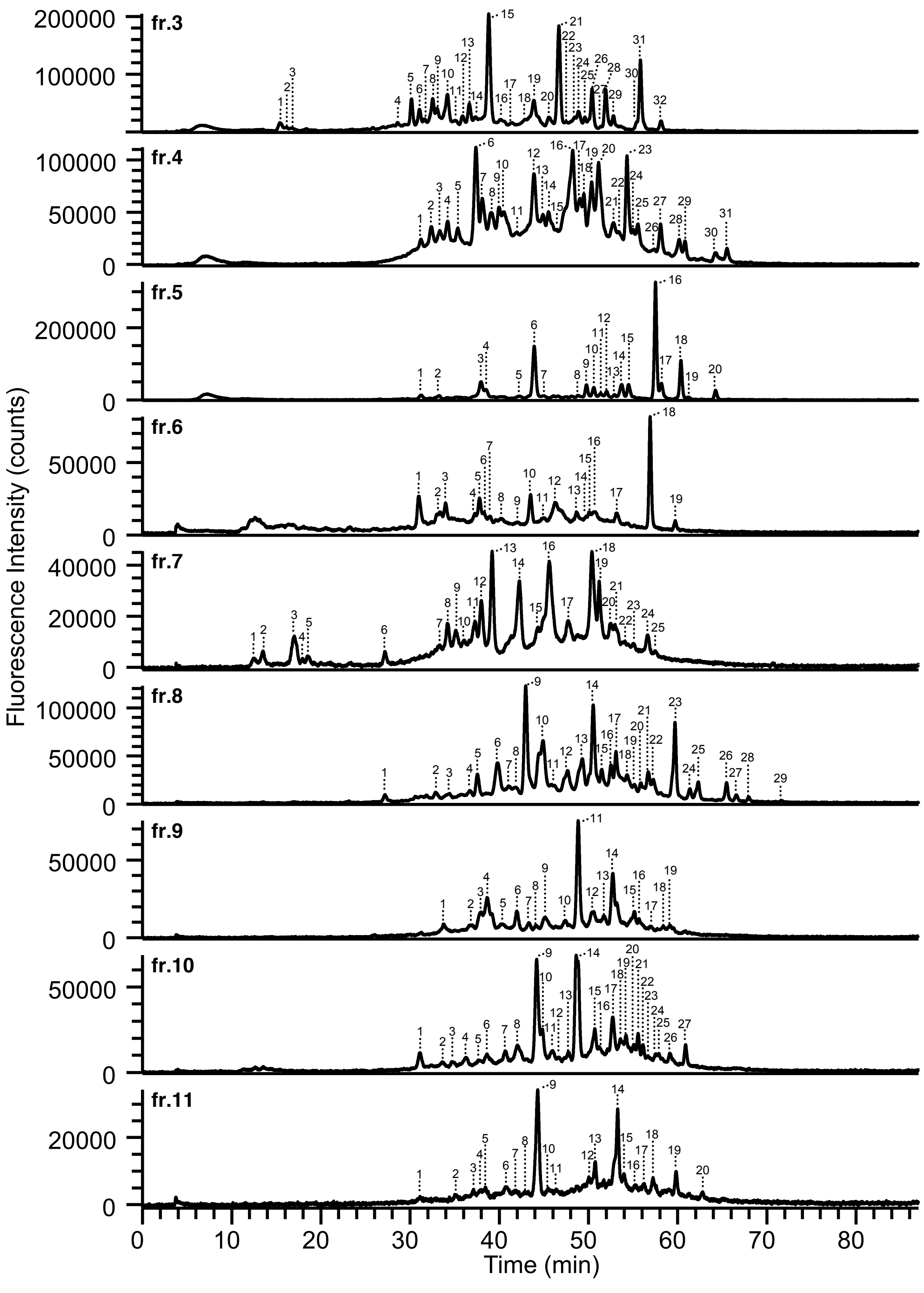

Figure S4. Elution profiles of alkylamidated PA-*N*-glycans from chicken colon on reversed-phase LC. Each fraction of PA-*N*-glycans from the DEAE column, except for the neutral fraction (fr.1) and non-glycan fraction (fr.2), was analyzed by LC-MS and MS/MS following the SALSA method. Most of the detectable peaks eluted in 10–87 min were numbered, regardless of whether they contain PA-*N*-glycans. The results of LC-MS and MS/MS are summarized in Supplementary Table S2.

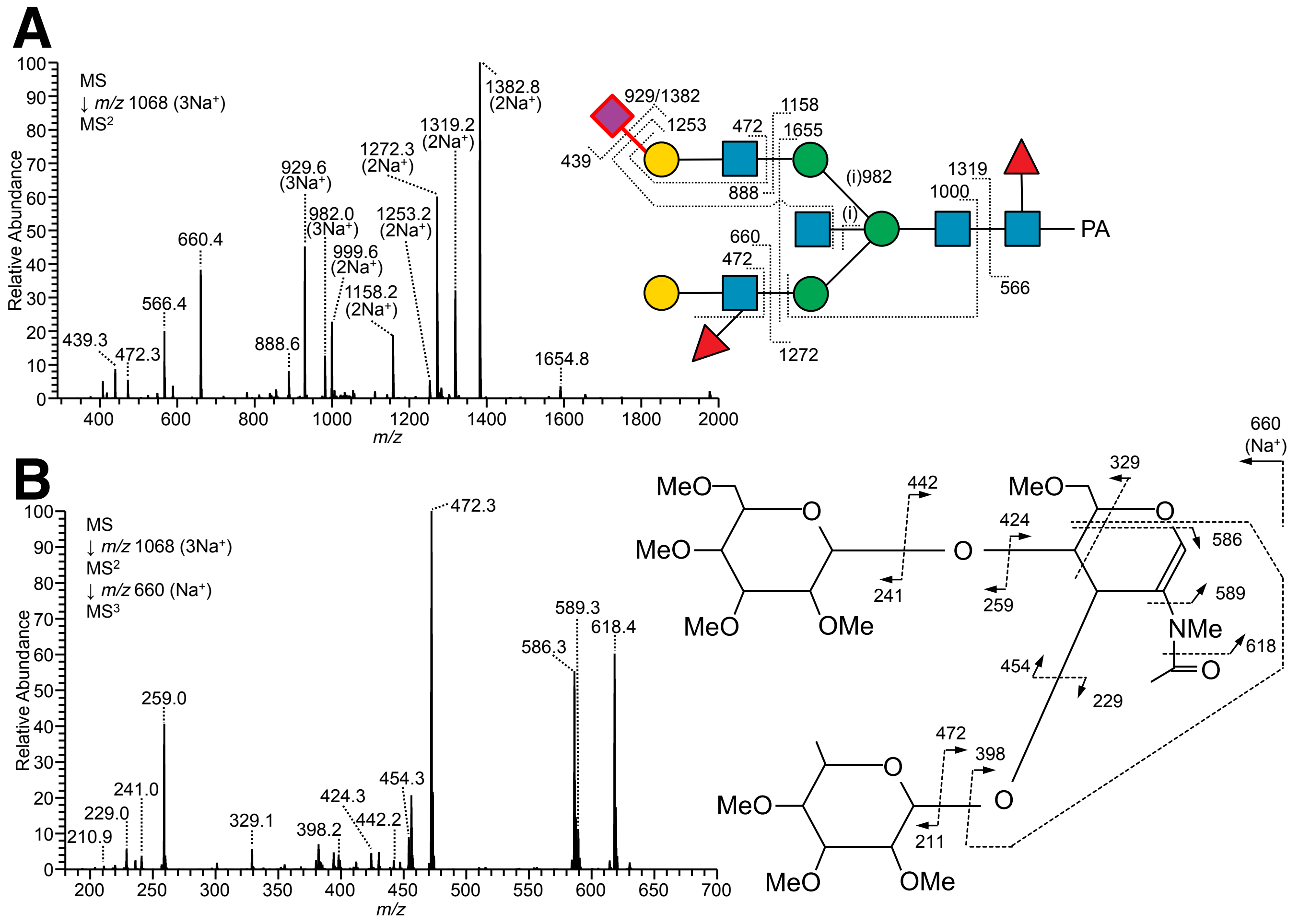

Figure S5-1. MS^n analysis of alkylamidated and permethylated PA-*N*-glycans from chicken colon. (A) MS^2 spectrum of PA-*N*-glycan with Le^x from the triply sodiated precursor ion at *m/z* 1068, corresponding to $Hex_2HexNAc_3Fuc_2(NA+iPA)_1C$-PA. The branch positions of each glycan sequence linked to α3- or α6-Man have not been determined. (B) MS^3 spectrum of the B ion fragments $Hex_1HexNAc_1Fuc_1$ at *m/z* 660.

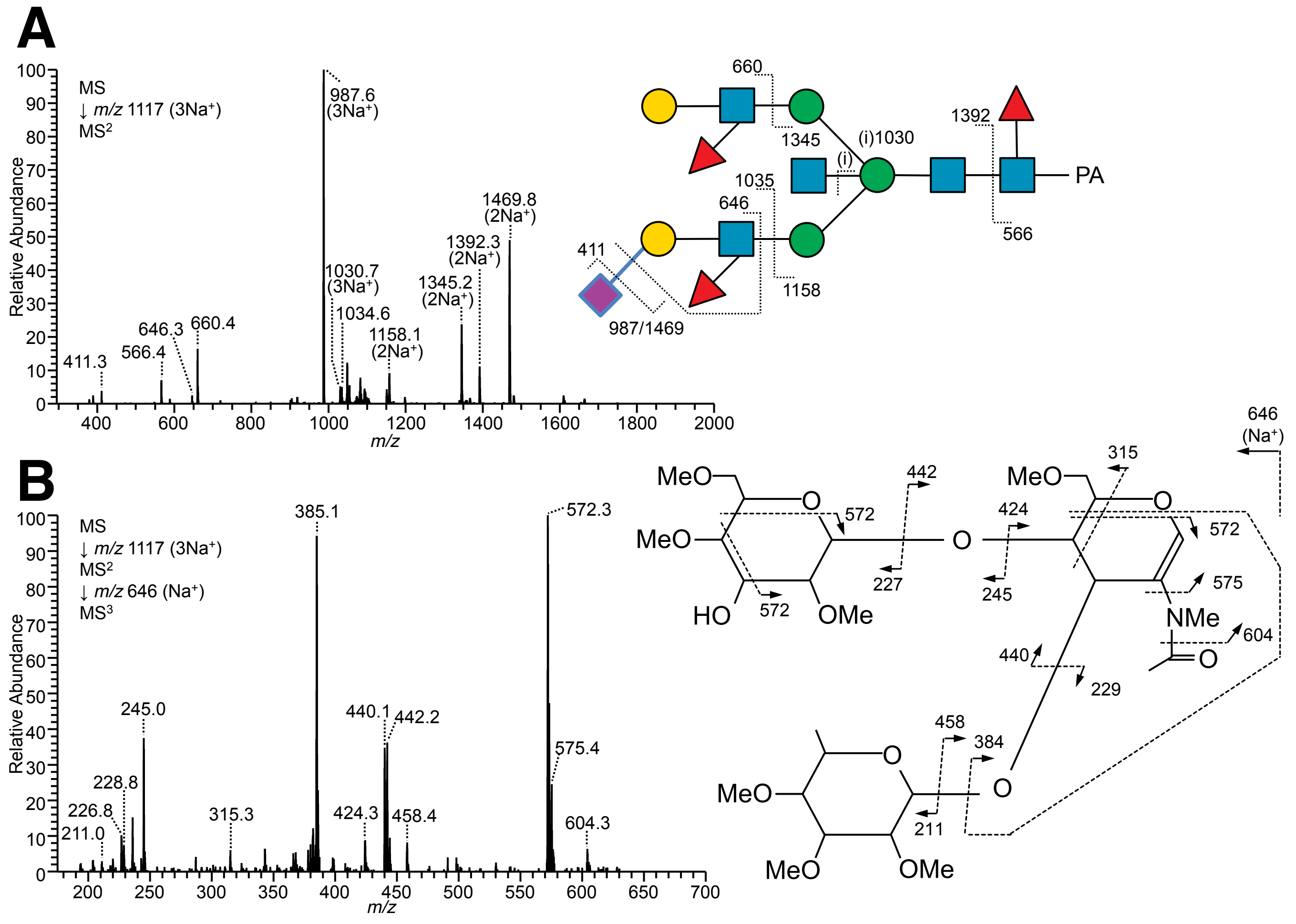

Figure S5-2. MS^n analysis of alkylamidated and permethylated PA-*N*-glycans from chicken colon. (A) MS^2 spectrum of PA-*N*-glycan with sLe^x from the triply sodiated precursor ion at *m/z* 1117, corresponding to $Hex_2HexNAc_3Fuc_3(NA+MA)_1C$-PA. The branch positions of each glycan sequence linked to α3- or α6-Man have not been determined. (B) MS^3 spectrum of the B/Y ion fragments $Hex_1HexNAc_1Fuc_1$ at *m/z* 646.

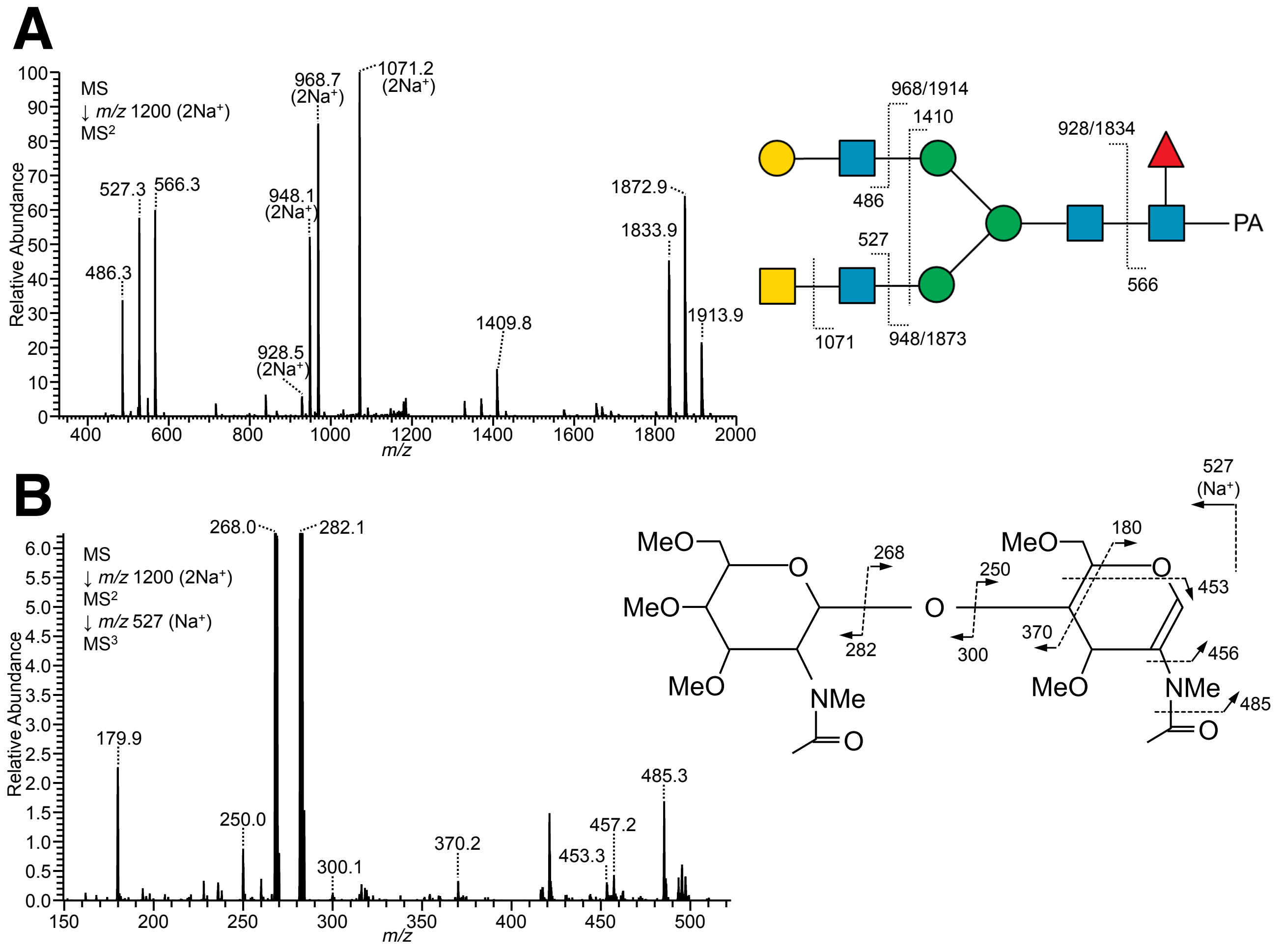

Figure S5-3. MS^n analysis of alkylamidated and permethylated PA-*N*-glycans from chicken colon. (A) MS^2 spectrum of PA-*N*-glycan with LacdiNAc from the doubly sodiated precursor ion at *m/z* 1200, corresponding to $Hex_1HexNAc_3Fuc_1C$-PA. The branch positions of each glycan sequence linked to α3- or α6-Man have not been determined. (B) MS^3 spectrum of the B ion fragments $HexNAc_2$ at *m/z* 527.

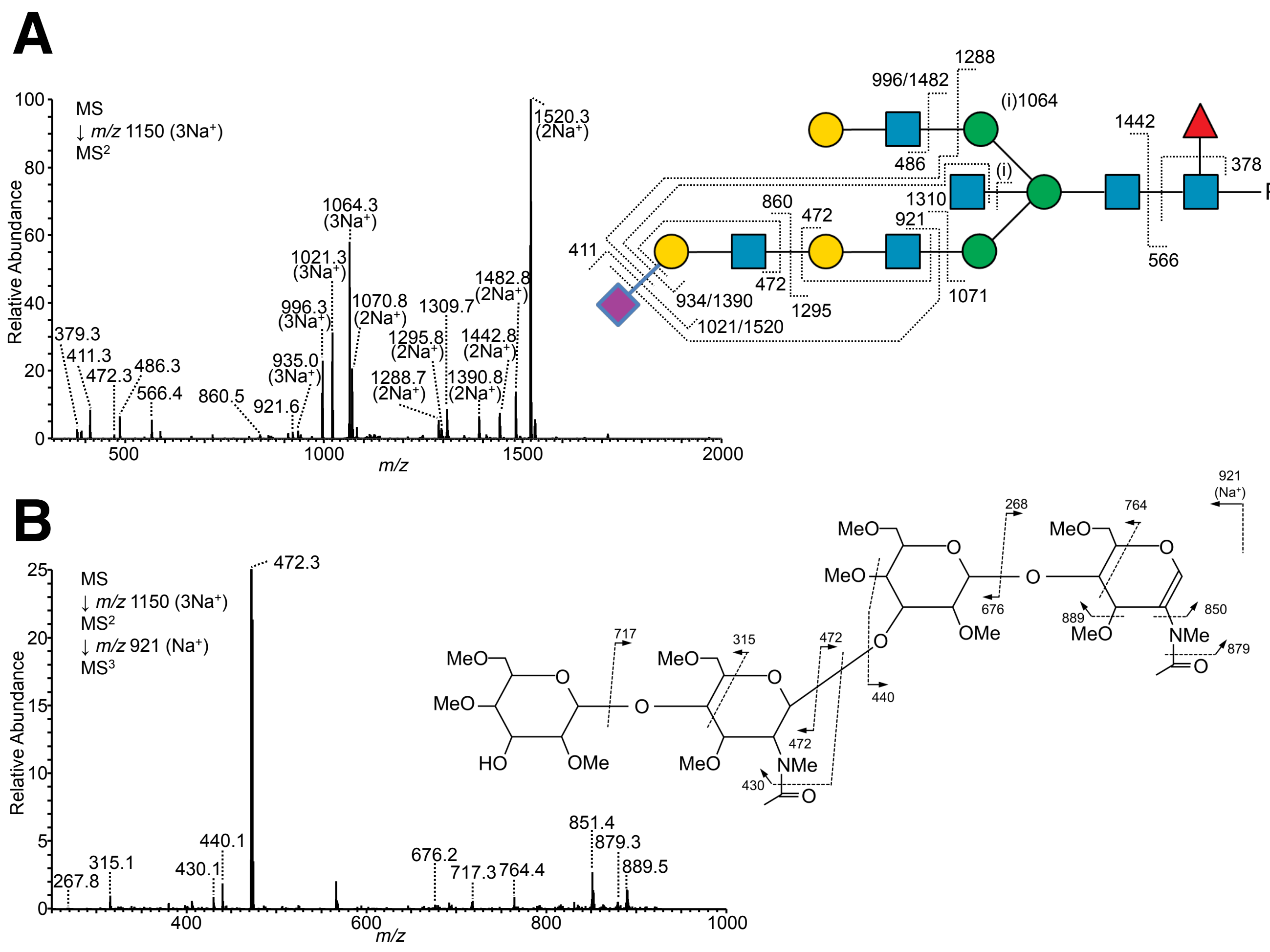

Figure S5-4. MS[n] analysis of alkylamidated and permethylated PA-*N*-glycans from chicken colon. (A) MS[2] spectrum of PA-*N*-glycan with α2,3-sialylated LacNAc repeat from the triply sodiated precursor ion at *m/z* 1150, corresponding to $Hex_3HexNAc_4Fuc_1(NA+MA)_1C$-PA. The branch positions of each glycan sequence linked to α3- or α6-Man have not been determined. (B) MS[3] spectrum of the B/Y ion fragments $Hex_2HexNAc_2$ at *m/z* 921. Although the linkage between two LacNAc residues is predicted to be -GalNAc1-3Gal- based on the known biosynthetic pathway for mammalian glycans, the actual linkage was not confirmed in this study.

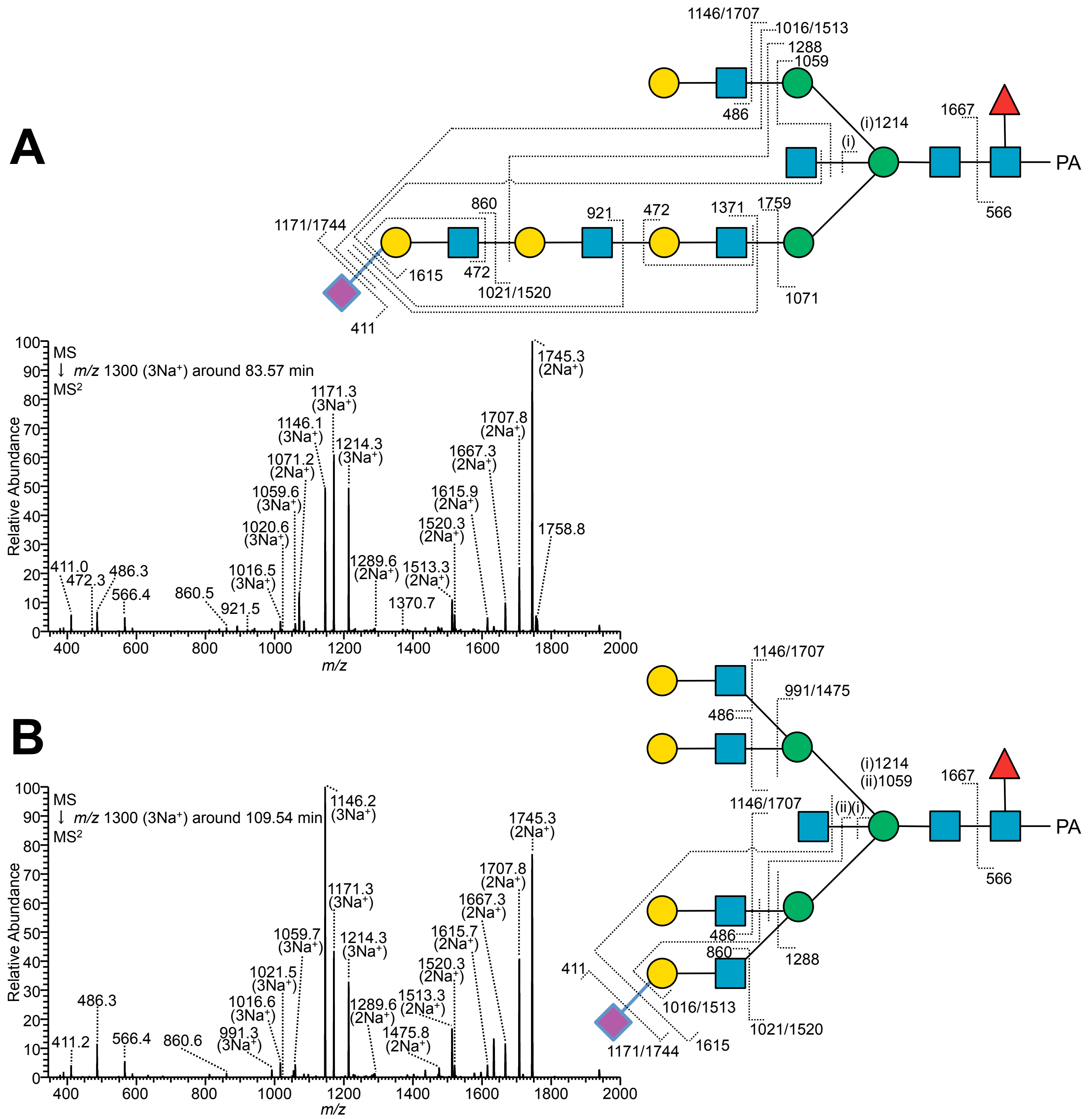

Figure S5-5. MS/MS analysis of alkylamidated and permethylated PA-*N*-glycans from chicken colon. MS/MS spectrum of PA-*N*-glycans from the triply sodiated precursor ion at *m/z* 1300, corresponding to $Hex_4HexNAc_5Fuc_1(NA+MA)_1C$-PA, eluted around 83.57 min (A) or 109.54 min (B) on reversed-phase LC. The branch positions of each glycan sequence linked to α3- or α6-Man have not been determined.
